# Genome-wide identification and expression pattern analysis of the SABATH gene family in *Neolamarckia cadamba*

**DOI:** 10.48130/FR-2023-0013

**Published:** 2023-05-29

**Authors:** Rongrong Ren, Suxia Zhang, Ting Guo, Jianmei Long, Changcao Peng

**Affiliations:** 1 Guangdong Key Laboratory for Innovative Development and Utilization of Forest Plant Germplasm, College of Forestry and Landscape Architecture, South China Agricultural University, Guangzhou 510642, China; 2 State Key Laboratory for Conservation and Utilization of Subtropical Agro-bioresources, South China Agricultural University, Guangzhou 510642, China

**Keywords:** SABATH gene family, Methyltransferase, Expression pattern, Neolamarckia cadamba, Cadambine

## Abstract

Plant SABATH methyltransferases are a class of enzymes that catalyze the transfer of the methyl group from S-adenosyl-L-methionine (SAM) to the carboxyl group or the nitrogen group of the substrate to form small molecule methyl esters or N-methylated compounds, which are involved in various secondary metabolite biosynthesis and have important impacts on plant growth, development, and defense reactions. We previously reported the monoterpenoid indole alkaloids (MIAs) cadambine biosynthetic pathway in *Neolamarckia cadamba*, a woody tree species that provides an important traditional medicine widely used to treat diseases such as diabetes, leprosy, and cancer in Southeast Asia. However, the functions of NcSABATHs in cadambine biosynthesis remain unclear. In this study, 23 *NcSABATHs* were identified and found to be distributed on 12 of the total 22 chromosomes. Gene structure, conserved motifs, and phylogenetic analysis showed that NcSABATHs could be divided into three groups. According to *cis*-element analysis, the *NcSABATH* promoters contained a large number of elements involved in light, plant hormone, and environmental stress responses, as well as binding sites for the BBR-BPC, DOF, and MYB transcription factor families. Based on RNA-seq data and qRT-PCR analysis, the *NcSABATH* genes exhibited diverse tissue expression patterns. Furthermore, *NcSABATH7*/*22*, which clustered with *LAMT* in the same clade, were both up-regulated under MeJA treatment. The correlation analysis between gene expression and cadambine content showed that NcSABATH7 potentially participated in cadambine biosynthesis. Taken together, our study not only enhanced our understanding of SABATH in *N. cadamba* but also identified potential candidate genes involved in cadambine biosynthesis.

## Introduction

Methylation occurs frequently in organisms. The production process of secondary metabolites such as alkaloids, flavonoids, phenylpropane, stilbene, anthraquinone, and lignin mostly requires methylation modifications. Plant *O*-methyltransferases (OMTs) can methylate oxygen atoms in secondary metabolites^[1]^, and methylation modification can improve lipid solubility and stability for better biological activity^[2]^. Plant SABATH methyltransferases are the most studied type III OMTs, which are named after the three first identified enzymes: salicylic acid carboxyl methyltransferases (SAMT)^[3]^, benzoic acid carboxyl methyltransferases (BAMT)^[4]^ and theobromine synthase (TCS)^[5]^. Plant SABATH methyltransferases use SAM as a methyl donor to catalyze the methylation of oxygen atoms on carboxyl or hydroxyl groups and nitrogen atoms of hormones and small molecule compounds, and the resulting reaction products are involved in the biosynthesis of various secondary metabolites and play important roles in plant growth and development and defense processes^[6]^. To date, SABATH proteins have been identified in a variety of species, such as *Arabidopsis thaliana* (24), *Oryza sativa* (21), *Populus trichocarpa* (28), *Salvia miltiorrhiza* (30), *Solanum lycopersicum* (20), *Camellia sinensis* (32) and others^[7−11]^.

In addition to the three first identified SAMT, BAMT, and TCS methyltransferases, other functional members of the SABATH family have gradually been identified in different species. In *A. thaliana*, jasmonic acid carboxyl methyltransferase (JMT)^[12]^, paraxanthine methyltransferase (PXMT)^[13]^, indoe-acetic acid carboxyl methyltransferase (IAMT)^[14]^, farnesoic acid carboxyl methyltransferase (FAMT)^[15]^, gibberellic acid carboxy methyltransferase (GAMT)^[16]^, benzoic/salicylic acid carboxyl methyltransferase (BSMT)^[17]^, nicotinate methyltransferase (NAMT)^[18]^ and carlactonoic acid methyltransferase (CLAMT)^[19]^ were identified. SA, JA, IAA, and other phytohormones are converted into methyl esters under different environmental conditions through the catalysis of methyltransferases, which improve plant resistance and participate in various plant developmental processes such as seed germination, flower and fruit development, leaf senescence and abscission by regulating the content of endogenous hormones in plants^[20−23]^. Then, two methyltransferases necessary for the synthesis of theobromine were discovered in coffee: xanthosine methyltransferase (XMT) and 3,7-dimethylxanthine methyltransferase (DXMT)^[24]^. The cinnamate/p-coumarate carboxyl methyltransferase (CCMT) was identified in *Ocimum basilicum*^[25]^. The loganic acid methyltransferase (LAMT) was first identified in *Catharanthus roseus* and was able to regulate the upstream synthesis pathway of the MIAs catharanthine and vindoline by specifically catalyzing loganic acid into loganin^[26,27]^. Anthranilic acid methyltransferase (AAMT), the herbivore-induced SABATH methyltransferase in maize, catalyzes anthranilic acid into methyl anthranilic acid to attract natural enemies of insect herbivores^[28]^.

*N. cadamba* belongs to the family of Rubiaceae, an evergreen tree with straight trunks and broad leaves, and is an excellent fast-growing tree for wood in South China. It is known as a 'miracle tree' due to its rapid growth. In addition, it is a traditional medicinal plant in Southeast Asia. Its roots, bark, leaves, and fruits are used to treat a variety of diseases such as diabetes, anemia, stomatitis, leprosy, diarrhea, cancer, and infectious diseases because of the abundance of secondary metabolites such as steroids, tannins, flavonoids, cadambine acid, quinovic acid and MIAs^[29]^. Recently, growing research suggests that the primary active compounds in *N. cadamba* are the glycosidic MIAs cadambine and its derivatives 3β-isodihydrocadambine and 3β-dihydrocadambine, which exhibit anti-inflammatory, analgesic, antidiabetic, antitumor and antimalarial activities^[30−32]^. We previously predicted the biosynthetic pathway of cadambine. However, it is still unknown whether NcSABATHs play a role in cadambine biosynthesis.

In this study, members of the SABATH gene family of *N. cadamba* were screened and identified using bioinformatics methods, and their gene structure, physicochemical properties, conserved domains and motifs, chromosomal locations, evolutionary relationships, and promoter *cis*-acting elements were analyzed. In addition, based on published transcriptome data and quantitative real-time polymerase chain reaction (qRT-PCR) analysis, the expression patterns of the *NcSABATH* gene family were analyzed, which provided a preliminary understanding of the potential functions of *NcSABATH* genes. Currently, an efficient protocol of plant regeneration and *Agrobacterium*-mediated genetic transformation for *N. cadamba* has been established^[33,34]^, laying the foundation for further verification of *NcSABATH* gene functions using molecular biotechnologies.

## Materials and methods

### Identification of SABATH gene family members in *N. cadamba*

The whole genome sequence and annotation files of *N. cadamba* were downloaded from the webpage (https://figshare.com/s/ed20e0e82a4e7474396b)^[35]^. The hidden Markov model (HMM) of the SABATH domain (PF03492) was downloaded in the Pfam database^[36]^, and the *NcSABATH* genes were searched in the *N. cadamba* protein database *via* hmm search using HMM files (E-value < 1e^−10^). The candidate protein sequences were then submitted to SMART (http://smart.embl-heidelberg.de/)^[37]^ and NCBI-CDD (https://www.ncbi.nlm.nih.gov/cdd/) databases to confirm the existence of the Methyltransf_7 domain. Then we further manually examined whether each candidate gene contained the conserved S-adenosyl-L-methionine (SAM) binding motifs^[38]^. The online tool ExPASy (https://web.expasy.org/protparam/) was used to analyze the amino acid number, isoelectric point (pI), and molecular weight (MW) of SABATH proteins. The online website Plant-mPLoc (http://www.csbio.sjtu.edu.cn/bioinf/plant-multi/) was used to predict subcellular location.

### Multiple sequence alignment and phylogenetic analysis

SABATH protein accession numbers belonging to *Atropa belladonna*, *Antirrhinum majus*, *A. thaliana*, *Coffea arabica*, *Clarkia breweri*, *Coffea canephora*, *C. roseus*, *Camellia sinensis*, *Nicotiana suaveolens*, *O. basilicum*, *Ophiorrhiza pumila*, *Oryza sativa*, *Picea abies*, *Picea glauca*, *Petunia×hybrida*, *Physcomitrella patens*, *Populus trichocarpa*, *Stephanotis floribunda*, *Selaginella moellendorffii*, *Zea mays*, *Nicotiana gossei* were listed in Supplemental Table S1^[8,11]^. Multiple sequence alignments of NcSABATH proteins were performed using the program ClustalW with default parameters and a neighbor-joining (NJ) phylogenetic tree was constructed using MEGA X software with 1,000 bootstrap replicates^[39]^. A maximum likelihood (ML) phylogenetic tree from different plants was constructed with 1,000 bootstrap replicates using the One Step Build a ML Tree function in the TBtools toolkit^[40]^, and then the tree was visualized using the iTOL (https://itol.embl.de/) online tool.

### Gene structure and conserved motifs analysis

The analysis and visualization of gene structure and conserved domains of the NcSABATHs were realized through TBtools. The conserved motifs in NcSABATH proteins were identified using Multiple Expectation Maximization for Motif Elicitation (MEME) v. 4.12.0 (http://meme-suite.org/). The parameters were set as follows: the number of repetitions was set to zero or one, and the maximum number of motifs was set to 10.

### Chromosomal locations and synteny analysis

TBtools was used to plot the location of the NcSABATH family on the chromosomes. Gene duplication and synteny relationship analysis of *N. cadamba* with *A. thaliana* and *P. trichocarpa* were performed using the One-Step MCScanX function in TBtools software, and the results were further visualized by the Advanced Circos and Multiple Synteny Plot functions. The ratios of the non-synonymous (*K*_*a*_) substitution rate and synonymous substitution rate (*K*_*s*_) of duplicated gene pairs were calculated using the Simple *K*_*a*_/*K*_*s*_ Calculator function of TBtools.

### Prediction of *cis*-acting elements and transcription factor binding sites in the proximal promoters of *NcSABATH* genes

The upstream 2,000 bp sequence from the start codon of *NcSABATH* genes was extracted from the *N. cadamba* genomic database. Promoter *cis*-acting elements and transcription factor binding sites prediction were analyzed using the online software PlantCARE (http://bioinformatics.psb.ugent.be/webtools/plantcare/html/)^[41]^ and PlantTFDB (http://planttfdb.gao-lab.org/blast.php)^[42]^, respectively. The results were visualized through TBtools.

### Expression pattern analysis based on the transcriptome data

The expression levels of the *NcSABATH* genes in 16 tissues including bark, bud, cambium, young fruit, old leaves, phloem, root, young leaves, as well as xylem (primary xylem, PX; transitional xylem, TX; secondary xylem, SX), cambium (transitional cambium, TCA; secondary cambium, SCA) and phloem (primary phloem, PPH; transitional phloem, TPH; secondary phloem, SPH ) from the first, second and fourth internodes were analyzed using RNA-seq data previously published by our group in the NCBI BioSample database under the accession number SAMN15700859 (Supplemental Table S2)^[35]^. Each sample was collected for three biological replicates. Transcript abundance was expressed as fragments per kilobase million (FRKM). The heat map is constructed by TBtools software and uses Log_2_-based FRKM.

### Plant materials, RNA extraction, and qRT-PCR

Roots, buds, phloem, old and young leaves were collected from five-year-old and healthy *N. cadamba* with a consistent genetic background on the campus of South China Agricultural University, Guangzhou, Guangdong, China. Each sample contained tissues of three biological replicates, with each replicate comprising mixed tissues from three individual trees. They were stored in liquid nitrogen until used. Before the MeJA (Methyl Jasmonic Acid) treatment, mature seeds of *N. cadamba* were collected on the campus of South China Agricultural University, soaked in water for 24 h, sterilized with 25% sodium hypochlorite solution for 10 min, followed by five times washing with sterile double-distilled water. Finally, they were sown on MS (Murashige and Skoog) solid medium and generated sterile seedlings. 7-week-old sterile seedlings were transplanted to sterile and clear glass containers (height, 10 cm; diameter, 6 cm) containing 40 mL of liquid rooting medium for growth and a paper bridge made out of a filter paper was placed to hold the seedlings in place (1 plant/container). MeJA (Sigma-Aldrich) was added to the liquid medium at a concentration of 100 μmol/L. The light intensity in the growth room was set at 90 μmol·m^−2^·s^−1^, the temperature was 22/18 ^◦^C (light/dark), photoperiod was 16/8 h. The composition of the liquid medium was: MS (4.74 g/L), sucrose (30 g/L), pH = 5.8. The MS solid medium was supplemented with agar (5.3 g/L). The roots, stems, and leaves of seedlings were taken at 0 h, 6 h, 12 h, 24 h, and 36 h after MeJA treatment, respectively. Each sample was collected for three biological replicates.

Total RNA was extracted from different tissue samples of *N. cadamba* using Protocol II of the E.Z.N.A.® Plant RNA Kit (Omega Bio-tek, Inc., Norcross, GA, USA). The HiScript® Ⅲ RT SuperMix for qPCR Kit (R323, Vazyme Biotech, Nanjing, China) was used for cDNA synthesis. Quantitative real-time PCR (qRT-PCR) was performed in LightCycler 480 (Roche Molecular Biochemicals, Mannheim, Germany) with the ChamQ Universal SYBR qPCR Master Mix (Q711, Vazyme Biotech, Nanjing, China). Three biological replicates and three technical replicates were performed. Relative expression levels were calculated by the 2^−ΔΔCᴛ^ method and visualized by GraphPad Prism 8. The *SAMDC* gene from *N. cadamba* was used as the internal reference gene^[43]^. The gene primers for qRT-PCR (Supplemental Table S3) were designed using NCBI-Primer Blast (https://www.ncbi.nlm.nih.gov/tools/primer-blast/) and the specificity of the primers was detected by PCR and gel electrophoresis.

### Determination of cadambine by LC-QQQ-MS/MS

The fresh tissue materials of different tissues were air dried at 55 °C until their weight was no longer changed, followed by crushing and sieving through 60 mesh. Powder (0.02 g) was dissolved in 1 mL of ethanol: water (7:3, v/v) and sonicated for 30 min, and then filtered through a 0.22 μm filter. Quantitative determination of cadambine content was performed by liquid chromatography tandem triple quadrupole mass spectrometry (LC-QQQ-MS/MS, Agilent1290-6470). The mobile phase comprised 0.2% formic acid (A) and methanol (B). A binary gradient elution was performed as follows: 3 min, 60% methanol; 5 min, 90% methanol; 6 min, 90% methanol; 6.5 min, 10% methanol, and 10 min, 10% methanol, column temperature, 40 °C.

## Results

### Genome-wide identification and physicochemical property analysis of the *SABATH* gene family in *N. cadamba*

To identify the *SABATH* gene family members in *N. cadamba*, we used the methyltransf_7 domain of SABATH as the query to perform the hidden Markov model (HMM) searching in *N. cadamba* genome with a parameter of 1e^−10^, and the candidate genes were submitted to the Pfam and NCBI-CDD databases to verify the presence of the methyltransf_7 domain. Finally, a total of 23 *NcSABATH* genes were identified and named *NcSABATH1*-*NcSABATH23* according to their chromosomal locations in *N. cadamba* (Table 1). The gene lengths of *NcSABATH* varied from 761 bp (*NcSABATH17*) to 29372 bp (*NcSABATH16*), and the lengths of the *NcSABATH* cDNAs varied from 405 bp (*NcSABATH17*) to 2181 bp (*NcSABATH2*), which encoded proteins varying from 134 aa (NcSABATH17) to 726 aa (NcSABATH2). The molecular weight of the proteins ranged from 15.2 kDa (NcSABATH6) to 82.6 kDa (NcSABATH2). The theoretical isoelectric points (pI) ranged from 4.71 (NcSABATH16) to 8.58 (NcSABATH17). Subcellular localization prediction analysis indicated that most of the NcSABATHs were located in the cytoplasm, while some of them might also be located in the nucleus.

**Table 1:** Molecular characteristics of *NcSABATH* genes in *N. cadamba*.

Gene name	Gene ID	Strand	Gene	CDS	Protein	pI	MW (kDa)	Predict
			Length (bp)	Length (bp)	Length (aa)			Subcellular localization
NcSABATH1	evm.model.Contig81.1046	−	2,611	1,119	372	5.44	41.6	Cytoplasm/Nucleus
NcSABATH2	evm.model.Contig54.5	+	6,169	2,181	726	7.05	82.6	Chloroplast/Nucleus
NcSABATH3	evm.model.Contig394.265	+	1,806	1,098	365	5.42	41.4	Cytoplasm/Nucleus
NcSABATH4	evm.model.Contig52.38	−	1,994	1,113	370	5.43	41.5	Cytoplasm
NcSABATH5	evm.model.Contig52.39	−	5,955	1,332	443	5.57	50	Cytoplasm/Nucleus
NcSABATH6	evm.model.Contig481.103	−	2,529	408	135	5.64	15.2	Cytoplasm
NcSABATH7	evm.model.Contig267.36	+	3,483	1,125	374	5.96	42	Cytoplasm
NcSABATH8	evm.model.Contig480.228	+	3,357	1,152	383	5.16	42.2	CytoplasmNucleus
NcSABATH9	evm.model.Contig69.51	−	1,574	576	191	4.78	21.7	Cytoplasm/Nucleus
NcSABATH10	evm.model.Contig69.50	−	1,574	576	191	4.78	21.7	Cytoplasm/Nucleus
NcSABATH11	evm.model.Contig21.35	−	1,968	1,095	364	5.16	40.9	Cytoplasm/Nucleus
NcSABATH12	evm.model.Contig45.442	+	1,953	1,149	382	5.97	42.9	Cytoplasm/Nucleus
NcSABATH13	evm.model.Contig66.900	−	828	735	244	6.83	27.2	Cytoplasm
NcSABATH14	evm.model.Contig154.585	−	2,437	729	242	5.4	27.3	Cytoplasm
NcSABATH15	evm.model.Contig555.236	+	2,570	1,062	353	5.43	39.6	Cytoplasm/Nucleus
NcSABATH16	evm.model.Contig437.21	−	29,372	576	191	4.71	21.7	Cytoplasm/Nucleus
NcSABATH17	evm.model.Contig371.14	−	761	405	134	8.58	15.3	Cytoplasm
NcSABATH18	evm.model.Contig371.16	−	3,054	1,065	354	5.98	40.6	Cytoplasm/Nucleus
NcSABATH19	evm.model.Contig892.11	+	2,354	1,050	349	5.2	39.2	Cytoplasm
NcSABATH20	evm.model.Contig139.120	+	8,367	789	262	6.46	29.9	Cytoplasm/Nucleus
NcSABATH21	evm.model.Contig139.217	−	4,010	1,155	384	5.49	42.5	Cytoplasm
NcSABATH22	evm.model.Contig625.59	+	3,023	1,128	375	6.07	42.3	Cytoplasm
NcSABATH23	evm.model.Contig1.15	−	2,354	1,050	349	5.28	39.2	Cytoplasm

### Phylogenetic analysis and classification of *NcSABATH* proteins

**Figure 1:**
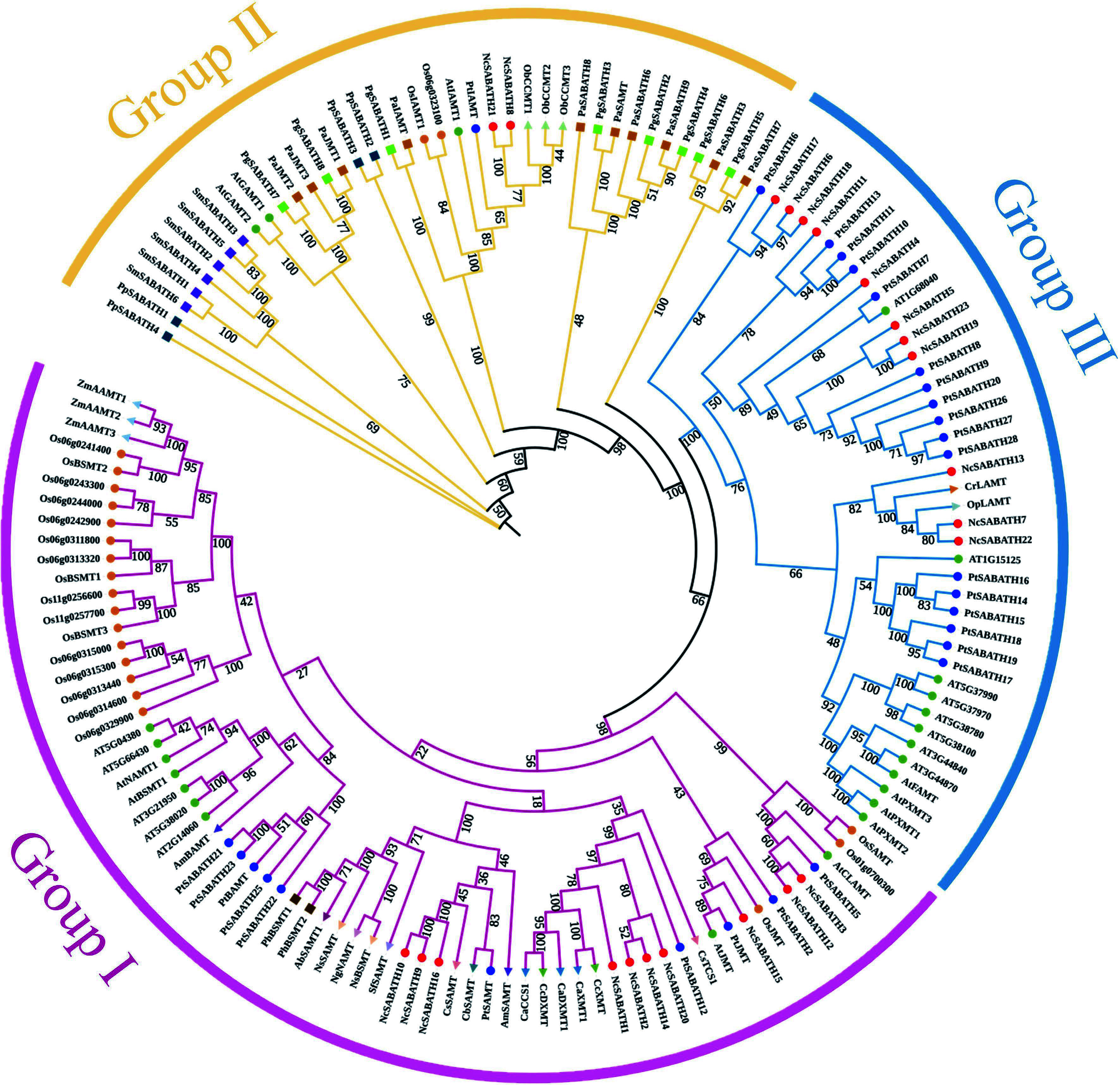
Phylogenetic analysis of SABATH proteins among 25 different plant species using the maximum likelihood method with 1,000 bootstrap replicates. The subfamilies of NcSABATH, group I, II, and III are marked with red, yellow, and blue, respectively. Details of all SABATH proteins are listed in Supplemental Table S2.

To clarify the evolutionary relationship and predict the putative function of the SABATH proteins in *N. cadamba*, a phylogenetic tree was constructed using 23 NcSABATH amino acid sequences and 126 sequences of SABATH protein members from 24 species of plants (Fig. 1, Supplemental Table S1). The results showed that all SABATHs were divided into three major groups (group I, group II, and group III). Group I contained 10 NcSABATH members, and they were clustered together with JMT, SAMT, XMT, DXMT, and CCS (bifunctional coffee caffeine synthase) from other species. NcSABATH9, NcSABATH10, and NcSABATH16 were clustered with CbSAMT, CsSAMT, and PtSAMT. NcSABATH15 was clustered with AtJMT and PtJMT. NcSABATH1, NcSABATH2, NcSABATH14, and NcSABATH20 were clustered with CcXMT/DXMT and CaXMT/DXMT. Group II is a highly conserved clade that contains IAMT, GAMT, and CCMT. The SABATH proteins of *P. patens*, *S. moellendorffii*, and gymnosperms (*P. glauca*, *P. abies*) were only clustered in this group. There are two NcSABATH members, NcSABATH8 and NcSABATH21, clustered together with AtIAMT and PtIAMT. The functions of the majority of proteins in Group III are unknown, except for FAMT, PXMT, and LAMT which have known functions. Group III contained 11 NcSABATH members, the most among the three groups. NcSABATH7, NcSABATH13, and NcSABATH22 were clustered with LAMT which is highly specialized for substrates and plays an important role in the iridoids branch pathway of alkaloids biosynthesis in *O. pumila* and *C. roseus*^[44,45]^*.* The remaining NcSABATHs were mostly clustered with uncharacterized functional SABATH proteins in *P. trichocarpa*. SABATH proteins with the same features and functions are grouped into the same clade. Thus, we could infer the functions of unknown SABATHs based on the clustering situation. In addition, putative paralogous genes were identified from the phylogenetic relationships. Paralogous genes usually display different functions^[46]^. According to the phylogenetic tree, there were seven pairs of paralogous genes identified among the 23 *NcSABATH* genes: *NcSABATH9* and *NcSABATH10*; *NcSABATH2* and *NcSABATH14*; *NcSABATH3* and *NcSABATH12*; *NcSABATH7* and *NcSABATH22*; *NcSABATH19* and *NcSABATH23*; *NcSABATH6* and *NcSABATH18*; *NcSABATH8* and *NcSABATH21*.

### Gene structure and conserved motifs analysis of *NcSABATH* proteins

**Figure 2:**
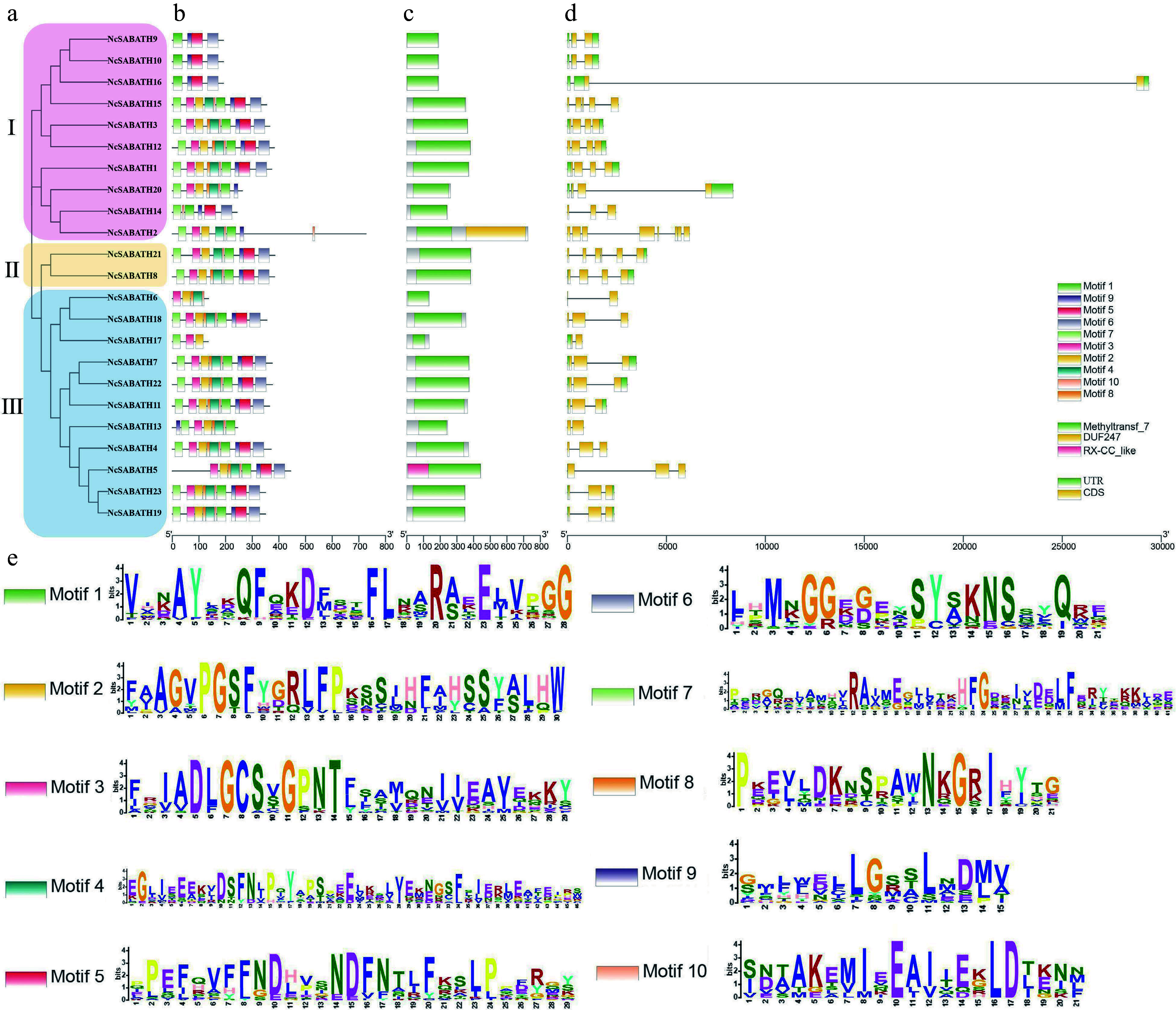
Phylogenetic tree, gene structure, conserved domain, and motif analysis of NcSABATH proteins from *N. cadamba*. (a) Phylogenetic tree of all NcSABATH proteins was constructed using the neighbor-joining method with 1000 bootstraps. (b) Conserved motifs in the NcSABATH proteins were identified by the MEME program. Different motifs numbered 1-10 have different colors, of which sequence logos are shown in (e). (c) The conserved domain of NcSABATH proteins. (d) The UTR, CDS, and intron organization of *NcSABATH* genes.

To investigate the structural diversity of *NcSABATH* genes in *N. cadamba*, the intron-exon structure of each gene was analyzed through its DNA sequence and CDS, which revealed that all coding sequences were interrupted by one or more introns (Fig. 2b). The intron numbers of the *NcSABATH* genes varied from 1 to 7. The number of exons varied from 2 to 8. The *NcSABATH* genes clustered on the same branch generally displayed similar intron-exon structures.

Online software MEME was used to predict the conserved motifs of *NcSABATH* genes (Fig. 2d). Finally, 10 motifs were identified. Each *NcSABATH* gene contained 4 to 10 motifs. The NcSABATH proteins in the same group also shared similar motif types and distribution patterns. Among the 10 conserved motifs, motif 1, 2, 3, 5, and 8 were located in the NcSABATH conserved domain, while the other 5 motifs were located outside the conserved domain. Most of the SABATH members in *N. cadamba* contained at least one functional motif (motif I and motif III), which is conserved in OMTs for binding to SAM. Motif 3 matched motif I while motif 1 matched motif III (Supplemental Fig. S1).

### Chromosomal distribution and collinearity analysis of NcSABATH proteins

A total of 22 *NcSABATH* gene family members were unevenly distributed across the 12 chromosomes, except *NcSABATH23* localized on the scaffold (Supplemental Fig. S2). There were four genes on chromosome 19, which contained relatively more *NcSABATH* members. Chromosomes 9 and 13 contained three genes. Chromosomes 10, 12 and 22 contained two genes, and Chromosomes 5, 6, 7, 14, 16, and 17 only contained one gene.

Gene families are generally formed by many processes including whole genome duplication, segmental duplication, tandem duplication, and transposable elements^[47]^. The expansion mechanism of the *NcSABATH* gene family was investigated by collinearity analysis. Therefore, we analyzed the collinearity of the SABATH genes within *N. cadamba* and the colinear relationship of the SABATH genes by comparing them with four other species. The results of intraspecies collinearity analysis showed that there were 12 duplicated gene pairs in the *NcSABATH* gene family. Only two tandem duplicated gene pairs of *NcSABATH* genes were detected (*NcSABATH4*-*NcSABATH5*, *NcSABATH9*-*NcSABATH10*) (Supplemental Fig. S2), and they were located on chromosomes 9 and 12 of *N. cadamba*, respectively. In addition, 69.57% (16/23) of the NcSABATH members underwent segmental duplication, which formed 10 segmental duplicated gene pairs and were found to be distributed on nine of the 22 chromosomes, indicating that segmental duplication probably played a leading role in the expansion of the *SABATH* gene family in *N. cadamba*. Among 10 segmental duplicated gene pairs, five pairs clustered in group III of the phylogenetic tree, followed by four pairs clustered in group I, and one pair clustered in group II. To further detect whether Darwinian positive selection participated in the driving of gene divergence after replication, *K*_*a*_/*K*_*s*_ ratios were calculated using the CDS of duplicated genes (Supplemental Table S6). The results showed that the ratios of *K*_*a*_/*K*_*s*_ for all duplicated genes were less than 1, implying that they were mainly subject to purifying selection. In other words, Darwinian positive selection was not involved in driving gene divergence after *NcSABATH* gene replication, and these duplicated genes might retain ancestral function.

**Figure 3:**
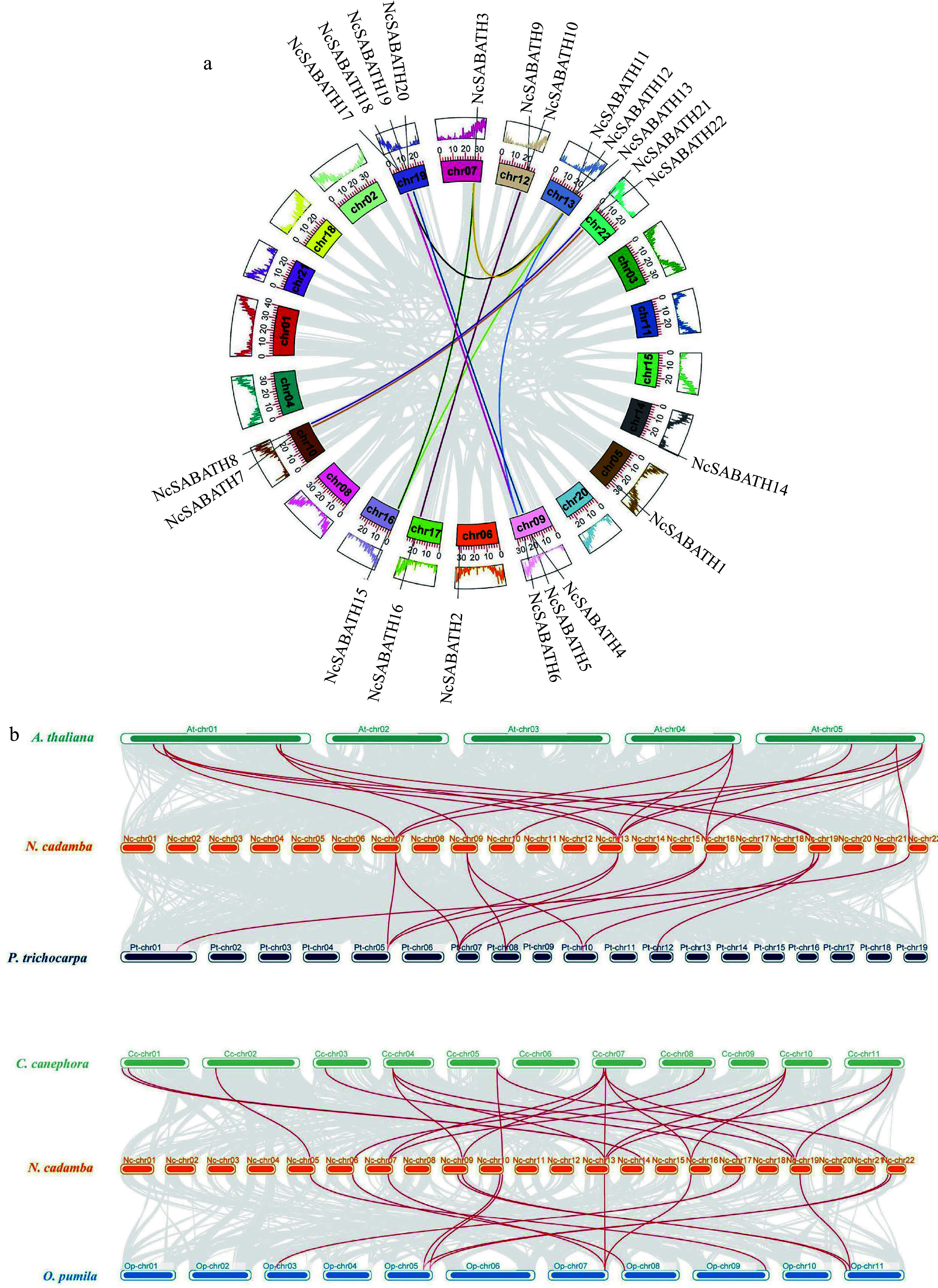
Collinearity analysis of *NcSABATH* genes. (a) Intraspecies collinearity analysis of *NcSABATHs*. Each segmental duplication gene pair was connected with the same color lines. (b) Collinearity analysis of *SABATHs* between *N. cadamba* and the other four species. *Arabidopsis thaliana* (At), *Populus trichocarpa* (Pt), *Coffea canephora* (Cc), *Ophiorrhiza pumila* (Op). Synteny blocks were represented by gray lines, and duplicated gene pairs of *SABATH* are represented by red lines.

To further analyze the inter-species colinear relationship of the *SABATH* genes, we constructed comparative colinear maps of *N. cadamba* with four representative species including *A. thaliana*, *P. trichocarpa*, *C. canephora*, and *O. pumila* at the whole genome level (Fig. 3b). The results showed that *NcSABATH* genes had collinearity with genes in the genomes of *A. thaliana*, *P. trichocarpa*, *C. canephora*, and *O. pumila*, and the number of collinear gene pairs among the five species was maintained differently. Thirteen *NcSABATH* genes showed collinear relationships with those in *O. pumila*, followed by *A. thaliana* (9) and *P. trichocarpa* (8). A total of 20 SABATH collinear gene pairs were identified between *N. cadamba* and *C. canephora*, followed by *N. cadamba* and *A. thaliana* (16), *N. cadamba* and *P. trichocarpa* (13), and *N. cadamba* and *O. pumila* (13). Among these collinear gene pairs, *NcSABATH3*, *12*, *15*, and *21* were detected in the collinear gene pairs between *N. cadamba* and all of the other four species, each of the three genes was collinear with three *AtSABATH* genes, as well as two *SABATH* genes in *C. canephora* and *P. trichocarpa*, which suggested that they might have a significant effect on the evolution of *SABATH* genes. In addition, only five *NcSABATH* genes (*NcSABATH5*, *9*, *10*, *11*, and *20*) shared no collinear relationship with *SABATH* genes of the other four species, implying that these genes may have unique functions in the evolution of *N. cadamba*. *N. cadamba*, *C. canephora*, and *O. pumila*, both belong to the Rubiaceae family and are close relatives, however, these plants produce three distinct secondary metabolites of cadambine, caffeine (purine alkaloids), and camptothecin (MIA), respectively. More than 55% (13/23) of *NcSABATH* genes were colinear with those in *C. canephora* and *O. pumila*, and one *NcSABATH* gene was associated with only one colinear gene pair between *N. cadamba* and *O. pumila* *SABATH* genes, while some *NcSABATH* genes had at least two colinear gene pairs with *C. canephora* *SABATH* genes, indicating that these *NcSABATH* genes were likely to be essential in the growth and development of these three species. It was notable that *NcSABATH7* and *NcSABATH22* were only colinear with *OpLAMT*, the key enzyme-encoding gene involved in camptothecin biosynthesis in *O. pumila* and no *SABATH* genes associated with *NcSABATH7/22* were discovered in *C. canephora*. Then, we compared the protein sequences of NcSABATH7 and NcSABATH22 with those of OpLAMT, CrLAMT and other reported LAMTs (Supplemental Fig. S3). The results showed that NcSABATH7 and NcSABATH22 shared more than 80% aa identity with OpLAMT and CrLAMT, and both NcSABTH proteins contained active sites that bind specifically to the substrates SAM and loganic acid (Y159, H162, W163, P227, A241, H245, Q273, H275, P302, Q316, I320, D359)^[48,49]^. Taken together, these results suggested that NcSABATH7 and NcSABATH22 might have conserved functions and were critical for *N. cadamba* to produce the specialized MIA cadambine.

### Prediction of *cis*-acting elements and transcription factor binding sites in the proximal promoters of *NcSABATH* genes

**Figure 4:**
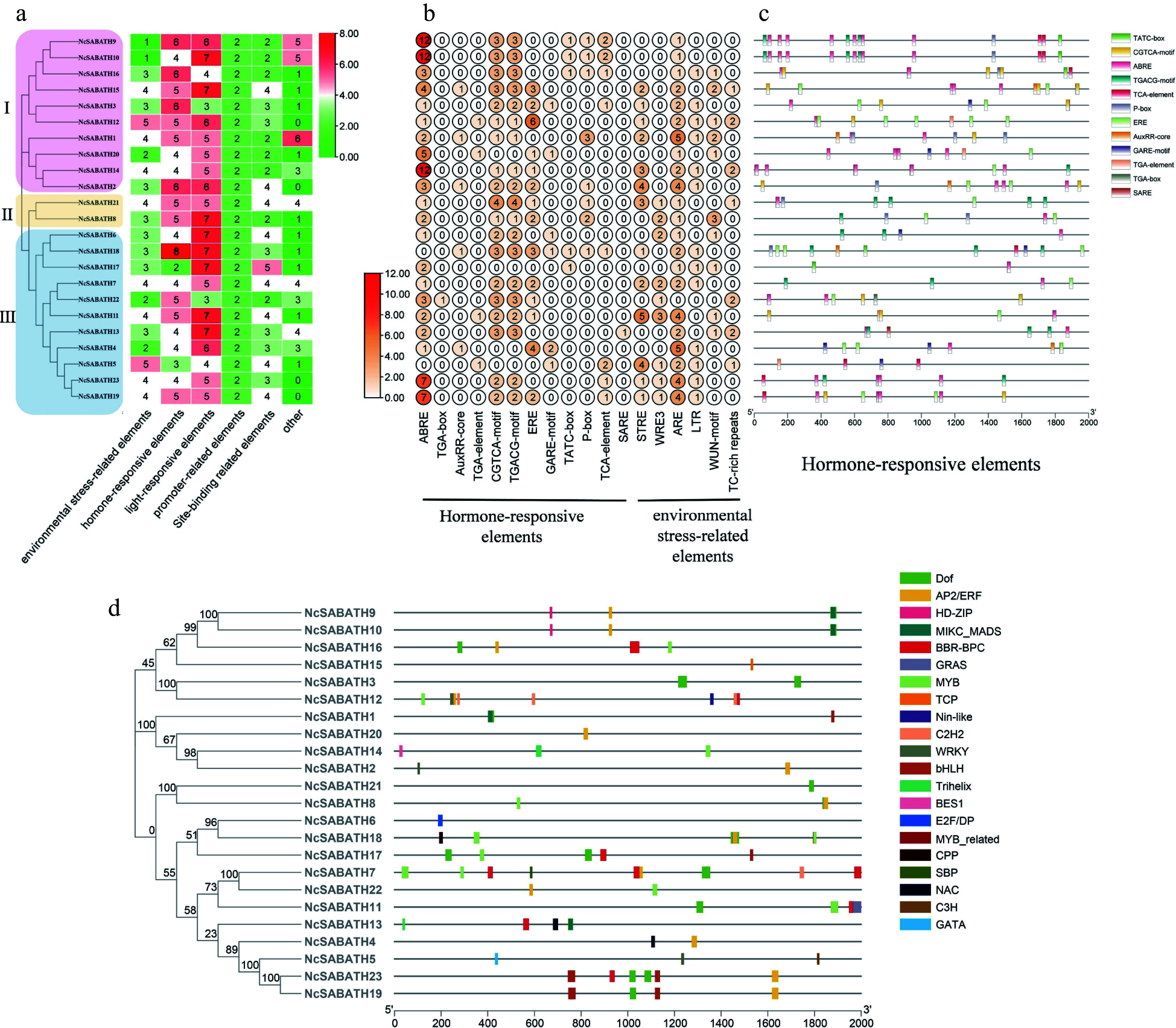
Prediction of *cis*-acting elements and transcription factor binding sites in the promoters of *NcSABATH* genes. (a) Each *NcSABATH* promoter contains the number of *cis*-acting elements detected which were divided into six types. (b) The number of different elements in hormone-responsive and environmental stress-related elements. (c) Visualization of hormone-responsive elements in *NcSABATH* promoters by TBtools, including position, kind, and quantity of elements. (d) Visualization of the number, type, and position of transcription factor binding sites in *NcSABATH* promoters by TBtools.

*Cis*-acting elements in the promoter region play an important role in regulating the biological functions of specific genes. In this study, the upstream 2000 bp sequence from the start codon of 23 *NcSABATH* genes was extracted from the *N. cadamba* genome information and analyzed using PlantCARE (Supplemental Table S4). In total, 62 different *cis*-acting elements were observed in all *NcSABATH* gene promoter regions. Among these elements, in addition to the typical TATA boxes and CAAT boxes that each *NcSABATH* gene possessed, other various *cis*-acting elements are mainly involved in light response, hormone response, environmental stress response, site binding, and other functions (Fig. 4a). Light-responsive elements include 20 types of elements, such as G-box, GT1-motif, Box 4, GATA-motif, and so on, among which G-box was the most abundant. Twelve kinds of hormone-responsive elements were detected, including abscisic acid-responsive elements (ABRE), auxin-responsive elements (TGA-element, TGA-box, and AuxRR-core), ethylene-responsive elements (ERE), gibberellin-responsive elements (GARE-motif, P-box, and ATC-box), MeJA-responsive elements (CGTCA-motif and TGACG-motif), salicylic acid-responsive elements (TCA-element and SARE), among which ABRE elements related to ABA were the most abundant, followed by CGTCA-motif and TGACG-motif, which respond to MeJA (Fig. 4c). There are six elements related to environmental stress, among which ARE element was the most widely distributed, almost all *NcSABATH* gene promoters contained at least one ARE element (Fig. 4b).

Promoter *cis*-elements combine with transcription factors (TFs) to regulate gene transcription and expression. Therefore, potential TFs binding to the *NcSABATHs* promoter were predicted by PlantTFDB (Supplemental Table S5). 21 TF family binding sites were identified in the promoters of the NcSABATH family including AP2/ERF, BBR-BPC, BES1, bHLH, C2H2, C3H, CPP, DOF, E2F/DP, GATA, GRAS, HD-ZIP, MIKC_MADS, MYB, MYB_related, NAC, Nin-like, SBP, TCP and WRKY (Fig. 4d). Among them, the number of BBR-BPC binding sites was the largest (67), followed by DOF and MYB (59 and 52, respectively), and the number of binding sites for BES1, C3H, CPP, E2F/DP, GATA, MYB_related, Nin-like, SBP, and TCP was the least with only one. Moreover, the TFs involved in the regulation of *NcSABATH7* were the most abundant, while *NcSABATH6*/*15*/*21* were the least abundant.

### Expression patterns of *NcSABATH* genes in different tissues

**Figure 5:**
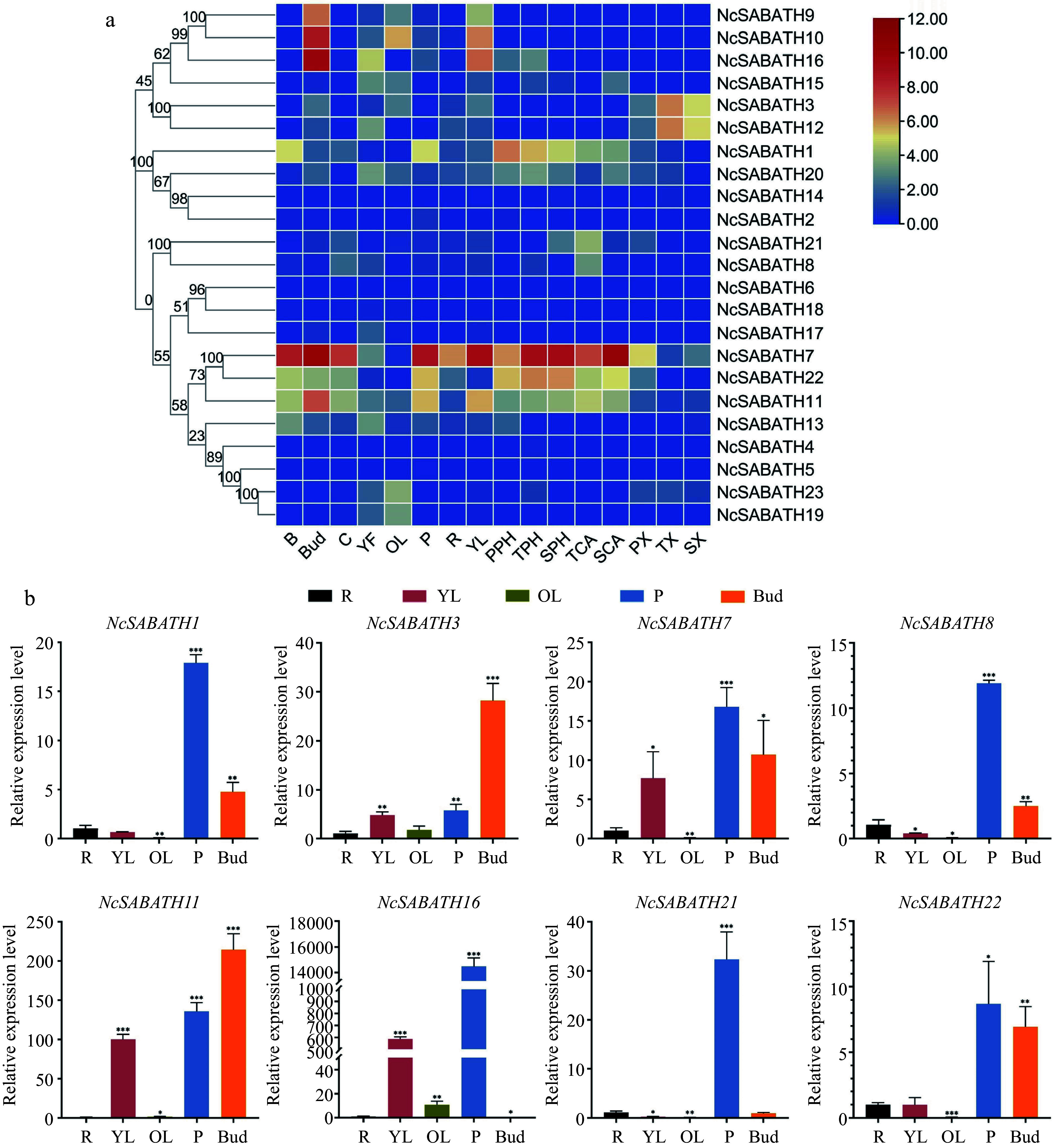
The expression patterns of *NcSABATH* genes in different tissues. (a) The expression patterns of *NcSABATH* genes in 16 tissues based on transcriptome data. Bark (B), cambium (C), bud (Bud), young fruit (YF), old leaves (OL), phloem (P), root (R), young leaves (YL), xylem (primary xylem, PX; transitional xylem, TX; secondary xylem, SX), cambium (transitional cambium, TCA; secondary, SCA) and phloem (primary phloem, PPH; transitional phloem, TPH; secondary phloem, SPH) from the first, second and fourth internodes. The second internode of a 1-year-old seedling was identified as the transition. The color scale represents relative expression levels from high (red color) to low (blue color). (b) QRT-PCR results of the eight selected *NcSABATHs* in roots (R), young leaves (YL), old leaves (OL), phloem (P), and bud of *N. cadamba.* Error bars represent ± SD of the means of three biological replicates (p < 0.05).

Based on transcriptome data from previous studies^[35]^, the expression patterns of all 23 *NcSABATH* genes in different tissues of *N. cadamba* were visualized by heatmap analysis (Fig. 5a). The results showed that, in a total of 16 tissues in *N. cadamba*, the expression of 22 *NcSABATH* genes was detected in at least one tissue (FPKM> 0) except for *NcSABATH6*, while *NcSABATH2*, *4*, *5*, *6*, *14* and *18* showed very low expression in all tissues (FPKM< 1). In addition, most of the *NcSABATH* genes showed tissue-specific expression patterns. For example, *NcSABATH9*, *10*, and *16* were mainly expressed in buds and young leaves. *NcSABATH3* and *12* showed high expression in xylem. *NcSABATH15*, *19*, and *23* were mainly expressed in fruit and old leaves. *NcSABATH8* and *NcSABATH21* had high expression in cambium. Notably, among the 23 *NcSABATH* genes, *NcSABATH1*, *7*, *11*, and *22* showed high expression in most of the tested tissues, especially in bark, bud, cambium, and phloem. The diverse expression patterns of *NcSABATH* genes indicated that they might display distinct functions in a variety of physiological processes of plant growth and development. To confirm the tissue expression of *NcSABATH* genes, a total of eight genes were selected from different groups based on phylogenetic relationships for qRT-PCR analysis (Fig. 5b), and the results were generally consistent with those from RNA-seq.

### Correlation analysis of *NcSABATH7*/*22* gene expression levels with the content of cadambine

**Figure 6:**
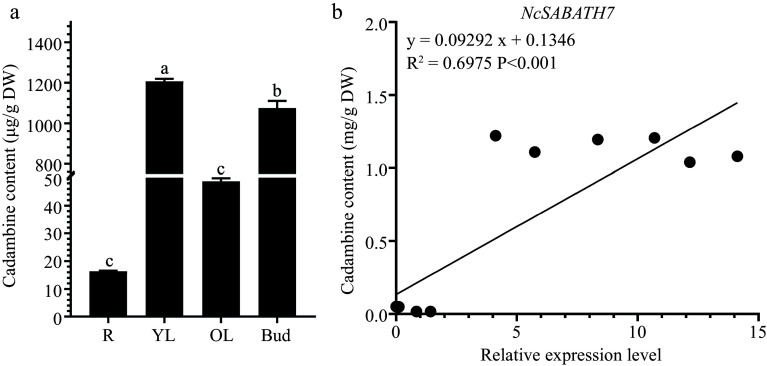
The content of cadambine in different tissues and correlation analysis between *NcSABATH7* gene expression level and cadambine content in different tissues of *N. cadamba*. (a) The content of cadambine in old leaves (OL), young leaves (YL), buds, and roots (R). One-way ANOVA (p < 0.05) was used to test significance, and different columns with the same letter showed no differences. (b) Correlation analysis between the expression level of *NcSABATH7* and cadambine content in different tissues of *N. cadamba*. Error bars represent ± SD of the means of three biological replicates.

LAMT is the only key rate-limiting enzyme that has been reported in the SABATH family to directly participate in the upstream biosynthetic pathway of MIAs and modulate the MIA accumulation in *C. roseus* and *O. pumila* which are famous MIA-producing plants. Among the 23 *NcSABATH* genes in *N. cadamba*, we identified two *LAMT* genes, *NcSABATH7* and *NcSABATH22*, which were orthologous with *CrLAMT* and *OpLAMT*. The content of cadambine in young leaves and buds was up to 1000 μg/g dry weight, while it was less in roots and old leaves (Fig. 6a). The correlation analysis of *NcSABATH7*/*22* gene expression levels with cadambine content in old leaves, young leaves, buds, and roots of *N. cadamba* revealed that *NcSABATH7* was significantly correlated with cadambine (R^2 ^= 0.6975, p < 0.001), whereas *NcSABATH22* was not (Fig. 6b). Considering that only one functional LAMT had been discovered in *C. roseus* and *O. pumila*, we proposed that NcSABATH7 might be involved in cadambine biosynthesis.

### Expression patterns of *NcSABATH* genes in response to MeJA

**Figure 7:**
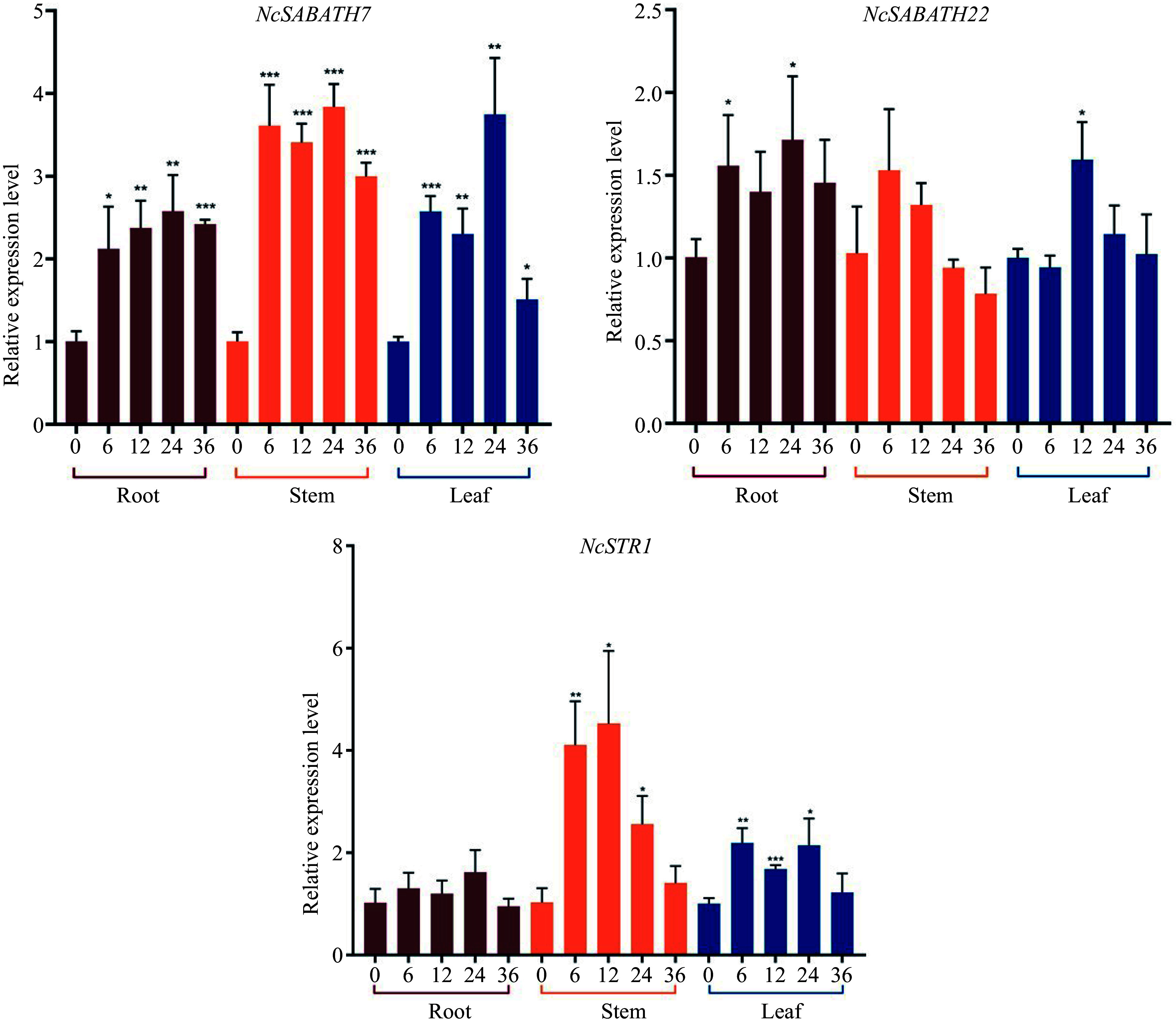
Relative expression levels of *NcSABATH7*/*22* and *NcSTR1* under MeJA (100 μM) stress in 0, 6, 12, 24, and 36 h. The mean expression value was calculated from three independent biological replicates relative to 0 h. Error bars represent ± SD of the means of three biological replicates (p < 0.05).

MeJA, a well-known regulator, can effectively induce over-accumulation of alkaloids by up-regulating the expression of the key genes involved in alkaloids biosynthesis in various medicinal plants such as *Artemisia annua*^[50]^, *A. belladonna*^[51]^, *C. roseus*^[52]^, *Camptotheca acuminata*^[53]^, *Dendrobium officinale*^[54]^ and *Opium Poppy*^[55]^. There are more than two MeJA response elements in the promoters of *NSABATH7* and *NcSABATH22.* Thus, to further explore the MeJA response patterns of these two genes, which are probably involved in the cadambine biosynthesis pathway, the relative expression levels of *NcSABATH7*/*22* in the roots, stems and leaves of *N. cadamba* treated with MeJA at different times were analyzed by qRT-PCR (Fig. 7). The results showed that the expression of *NcSABATH7* and *NcSABATH22* were both up-regulated at different levels under MeJA treatment and displayed distinct responses. *NcSABATH7* was significantly induced by MeJA in roots, stems, and leaves, and the response intensity increased over time. The expression of *NcSABATH22* was only slightly up-regulated in roots and leaves and was transiently elicited, but no significant difference was observed in stems. Moreover, *NcSTR1* (strictosidine synthase 1) is a key downstream gene that has been characterized to be involved in cadambine biosynthesis in *N. cadamba*^[35]^*.* Based on the previous finding that *NcSABATH7* and *22* were highly co-expressed with *NcSTR1 *(Supplemental Fig. S4), we further examined the relative expression level of *NcSTR1* under MeJA treatment. It was obvious that the expression of *NcSTR1* was significantly up-regulated in stems and leaves after MeJA treatment, and the expression pattern of *NcSTR1* was similar to that of *NcSABATH7.* As a result, NcSABATH7 is speculated to be involved in JA signaling-mediated transcriptional regulation of cadambine metabolism.

## Discussion

The SABATH gene family, which is ubiquitous in higher plants, is a group of key enzymes involved in the regulation of various biological processes ranging from plant growth and development to plant interactions with the environment by converting small active molecules into their methyl esters^[6]^. *SABATH* genes have been identified in various species, such as *Arabidopsis*, rice, tomato, and poplar. However, it has not been reported in *N. cadamba*. In this study, a total of 23 SABATH family members were identified from the *N. cadamba* genome. The number of *SABATH* genes in *N. cadamba* was smaller than those of *A. thaliana* (24), *P. trichocarpa* (28), *S. miltiorrhiza* (30), and *C. sinensis* (32), but larger than those of *O. sativa* (21), *S. lycopersicum* (20), *P. abie*s (10), and *Phaseolus vulgaris* (18)^[7−11,56,57]^. The 23 *NcSABATH* genes were divided into three distinct groups based on the phylogenetic trees constructed by the NJ method and ML method (Fig. 1), among which 11 *NcSABATH* genes were assigned to group Ⅲ with the largest proportion and two genes were assigned to group Ⅱ with the smallest proportion. *NcSABATH* genes in each group have similar gene structures and motif types. The *SABATH* genes reported in *A. thaliana* and *P. trichocarpa* were also divided into three groups based on evolutionary relationships, gene structures, and motif types^[7−8]^. In addition, according to the phylogenetic tree constructed by NcSABATH and other species of SABATH proteins, the SABATH proteins catalyzing the same substrate clustered in one branch, except for the SABATH proteins of gymnosperms (*P. abies* and *P. glauca*), which all clustered in Group II. This result also further supports the previous view that the *SABATH* genes evolved independently in angiosperms and were not clustered with gymnosperms^[58]^. Gene duplication events are critical for the proliferation of *SABATH* genes. Collinearity analysis indicated that segmental duplication is the dominant mechanism that promotes the expansion of the SABATH gene family in *N. cadamba.* The number of colinear gene pairs was correlated with the evolutionary distance between species. *N. cadamba* is evolutionarily closer to *C. canephora*^[35]^, so the number of colinear gene pairs was larger than with the other three species.

Previous studies showed that SABATHs may play important roles under biotic and abiotic stresses. For example, the expression levels of most *CsSABATH* genes in *C. sinensis* were up-regulated by *Ectropis oblique*, and *CsSABATHs* displayed distinct expression patterns in response to SA, JA, MeJA, and MeSA treatments, suggesting that CsSABATHs have specific functions in the defense response of the tea plant^[11]^. Drought and salt stress increased the expression levels of *Pvul-SABATH* genes in *Phaseolus vulgaris*^[57]^*.* Some *SlSABATH* genes in *S. lycopersicum* were significantly induced by cold, drought, and *Pseudomonas* *syringae* pv. tomato DC3000 stress treatments^[10]^. We discovered that *NcSABATH7*/*22*, which clustered with *LAMT*, were significantly induced after MeJA treatment, and their roles in the defense response of *N. cadamba* should be further investigated. We also discovered a large number of *cis*-acting elements involved in light response, various plant hormone responses, and environmental stress responses on the promoter of the *NcSABATH* genes, which indicated that *NcSABATH* genes may be essential to hormone signaling and defense responses. In addition, most *NcSABATH* genes showed tissue-specific expression patterns, and their functions can be estimated based on expression patterns and phylogenetic analysis. *NcSABATH9*, *10*, and *16* were clustered with *CbSAMT*, *CsSAMT*, and *PtSAMT* and mainly expressed in buds and young leaves. *CsSAMT* was also highly expressed in young leaves and participated in plant defense responses in tea plants^[11]^. *NcSABATH8* and *NcSABATH21* were specifically expressed in cambium and grouped into a branch with *IAMT*, an ancient member of the SABATH family, which influences IAA homeostasis in plants and regulates plant growth and development^[58]^. Therefore, it is suggested that NcSABATH8/21 may regulate secondary growth by influencing plant IAA content in *N. cadamba*. In summary, *NcSABATH* genes with different expression patterns might exhibit different functions for growth and development in *N. cadamba*. In this study, we focused on SABATH methyltransferases involved in cadambine biosynthesis. Methylation processes catalyzed by methyltransferases are indispensable in cadambine biosynthesis. LAMT has been identified as a critical rate-limiting enzyme in plants that produce MIAs and can efficiently regulate the synthesis and accumulation of MIAs. For example, *CrLAMT* was silenced in the leaves of *C. roseus* using virus-induced gene silencing (VIGS) technology, which resulted in a significant reduction of the amounts of catharanthine and vindoline^[27]^. Overexpression of *OpLAMT1* significantly increased the content of camptothecin in hairy root lines of *O. pumila* but camptothecin was hardly detected in *OpLAMT1* knock-out transgenic hairy roots^[49]^. In previous studies, the comparative genomic analysis revealed that *N. cadamba* diverged around 31.0 million years ago from *C. canephora*, later than *O. pumila* (41.7 million years ago), and underwent a relatively recent genome-wide duplication (WGD) after diverging from *C. canephora*^[35]^. Interestingly, we found that *NcSABATH7* and *NcSABATH22* were only colinear with *OpLAMT*, and no colinear gene pair associated with *NcSABATH7 and NcSABATH22* was discovered in *C. canephora.* This viewpoint is supported further by evolutionary relationships. NcSABATH7 and 22 were clustered with OpLAMT in Group III, while CcXMT/CcDXMT, the N-methyltransferases required for caffeine production, were clustered in Group I and no homologs of CcXMT or CcDXMT were found in the NcSABATH gene family. Therefore, we speculate that LAMT is critical for *N. cadamba* to produce the specialized MIA cadambine. *NcSABATH7* and *22* exhibited similar expression patterns in most tissues, except for young leaves. *NcSABATH7* was highly expressed in young leaves with high cadambine content, while *NcSABATH22* was barely expressed (Fig. 5). The correlation analysis of gene expression and cadambine content in different tissues showed that NcSABATH7 was significantly correlated with cadambine and NcSABATH22 was not (Fig. 7). And *NcSABATH7* responded more strongly to MeJA and showed a similar MeJA-induced expression pattern to *NcSTR1*, the only key gene that has been functionally characterized for cadambine biosynthesis^[35]^. Based on the differences in expression patterns of the two copies of the *LAMT* gene in *N. cadamba*, as well as the fact that there is only one functional LAMT in *C. roseus* and *O. pumila*, it is proposed that NcSABATH7 may be the key enzyme in cadambine biosynthesis, and NcSABATH22 may be inactive or generate novel functions. Additional experiments are needed to confirm this claim.

## Conclusions

In this study, a total of 23 *NcSABATH* genes were identified and found to be distributed on 12 of the 22 chromosomes. Based on their gene structures, conserved motifs, and phylogenetic relationships, they were divided into three groups, and SABATHs catalyzing the same substrate clustered into one clade. In addition, the specific expression patterns of *NcSABATH* genes in different tissues provided clues to explore their functions in these tissues. It is worth noting that *NcSABATH7*/*22* clustered in the same clade with *LAMT* and were significantly induced after MeJA treatment, and *NcSABATH7* exhibited similar MeJA response patterns to the key intermediate gene *NcSTR1*. The correlation analysis between gene expression and cadambine content revealed that NcSABATH7 is potentially involved in cadambine biosynthesis. This study lays the foundations for future functional characterization of NcSABATHs and provides candidate genes for regulating cadambine biosynthesis in *N. cadamba*.

## SUPPLEMENTARY DATA

Supplementary data to this article can be found online.

## References

[1] (2001). Structures of two natural product methyltransferases reveal the basis for substrate specificity in plant O-methyltransferases. Nature Structural Biology.

[2] (2021). Application of methyltransferases in microbial synthesis of natural products. Chinese Journal of Biotechnology.

[3] (1999). S-Adenosyl-L methionine: salicylic acid carboxyl methyltransferase, an enzyme involved in floral scent production and plant defense, represents a new class of plant methyltransferases. Archives of Biochemistry and Biophysics.

[4] (2000). Purification and characterization of S-adenosyl-l-methionine: benzoic acid carboxyl methyltransferase, the enzyme responsible for biosynthesis of the volatile ester methyl benzoate in flowers of *Antirrhinum majus*. Archives of Biochemistry and Biophysics.

[5] (1999). Purification and characterization of caffeine synthase from tea leaves. Plant Physiology.

[6] (2010). Methylation of phytohormones by the SABATH methyltransferases. Chinese Science Bulletin.

[7] (2019). Genome-wide comprehensive analysis of the SABATH gene family in *Arabidopsis* and rice. Evolutionary Bioinformatics.

[8] (2018). Evolution and function of the *Populus* SABATH family reveal that a single amino acid change results in a substrate switch. Plant and Cell Physiology.

[9] (2017). Genome-wide comprehensive analysis the molecular phylogenetic evaluation and tissue-specific expression of SABATH gene family in *Salvia miltiorrhiza*. Genes.

[10] (2021). Identification of *SABATH* family members in *Solanum lycopersicum* and their expression patterns under abiotic/biotic stresses. Plant Molecular Biology Reporter.

[11] (2020). Genome-wide identification and expression analysis of *SABATH* methyltransferases in tea plant (*Camellia sinensis*): insights into their roles in plant defense responses. Plant Signaling & Behavior.

[12] (2001). Jasmonic acid carboxyl methyltransferase: a key enzyme for jasmonate-regulated plant responses. Proceedings of the National Academy of Sciences of the United States of America.

[13] (2016). Light-inducible miR163 targets *PXMT1* transcripts to promote seed germination and primary root elongation in Arabidopsis. Plant Physiology.

[14] (2005). An indole-3-acetic acid carboxyl methyltransferase regulates *Arabidopsis* leaf development. The Plant Cell.

[15] (2006). An *Arabidopsis thaliana* methyltransferase capable of methylating farnesoic acid. Archives of Biochemistry Biophysics.

[16] (2007). Methylation of gibberellins by *Arabidopsis* GAMT1 and GAMT2. The Plant Cell.

[17] (2010). Enzymatic, expression and structural divergences among carboxyl *O*-methyltransferases after gene duplication and speciation in *Nicotiana*. Plant Molecular Biology.

[18] (2018). MeNA, controlled by reversible methylation of Nicotinate, is an NAD precursor that undergoes long-distance transport in *Arabidopsis*. Molecular Plant.

[19] (2022). A carlactonoic acid methyltransferase that contributes to the inhibition of shoot branching in *Arabidopsis*. Proceedings of the National Academy of Sciences of the United States of America.

[20] (2007). Methyl salicylate is a critical mobile signal for plant systemic acquired resistance. Science.

[21] (2008). Methyl jasmonate-elicited herbivore resistance: does MeJA function as a signal without being hydrolyzed to JA?. Planta.

[22] (2014). Jasmonoyl-L-isoleucine coordinates metabolic networks required for anthesis and floral attractant emission in wild tobacco (*Nicotiana attenuata*). The Plant Cell.

[23] (2008). The possible action mechanisms of indole-3-acetic acid methyl ester in *Arabidopsis*. Plant Cell Reports.

[24] (2007). Cloning, expression, crystallization and preliminary X-ray analysis of the XMT and DXMT *N*-methyltransferases from *Coffea canephora* (*robusta*). Acta Crystallographica Section F.

[25] (2007). Evolution of cinnamate/*p*-coumarate carboxyl methyltransferases and their role in the biosynthesis of methylcinnamate. The Plant Cell.

[26] (2008). The leaf epidermome of *Catharanthus roseus* reveals its biochemical specialization. The Plant Cell.

[27] (2013). A 7-deoxyloganetic acid glucosyltransferase contributes a key step in secologanin biosynthesis in Madagascar periwinkle. The Plant Cell.

[28] (2010). Herbivore-induced SABATH methyltransferases of maize that methylate anthranilic acid using s-adenosyl-L-methionine. Plant Physiology.

[29] (2016). Traditional uses, phytochemistry and pharmacological properties of *Neolamarckia cadamba*: a review. Journal of Ethnopharmacology.

[30] (2022). Differences in the components of *Neolamarckia cadamba* from different provenance and the drug resistance reversal activity of characteristic alkaloid. Industrial Crops and Products.

[31] (2020). Anti-inflammatory and analgesic activities of *Neolamarckia cadamba* and its bioactive monoterpenoid indole alkaloids. Journal of Ethnopharmacology.

[32] (2012). Evaluation of antitumor activity and in vivo antioxidant status of *Anthocephalus cadamba* on Ehrlich ascites carcinoma treated mice. Journal of Ethnopharmacology.

[33] (2019). Plant regeneration and *Agrobacterium*-mediated transformation of the miracle tree *Neolamarckia cadamba*. Industrial Crops and Products.

[34] (2020). High frequency regeneration of plants via callus-mediated organogenesis from cotyledon and hypocotyl cultures in a multipurpose tropical tree (*Neolamarkia Cadamba*). Scientific Reports.

[35] (2022). Chromosome-level assembly of the *Neolamarckia cadamba* genome provides insights into the evolution of cadambine biosynthesis. The Plant Journal.

[36] (2021). Pfam: the protein families database in 2021. Nucleic Acids Research.

[37] (2021). SMART: recent updates, new developments and status in 2020. Nucleic Acids Research.

[38] (1998). Conserved sequence motifs in plant S-adenosyl-L-methionine-dependent methyltransferases. Plant Molecular Biology.

[39] (2018). MEGA X: molecular evolutionary genetics analysis across computing platforms. Molecular Biology and evolution.

[40] (2020). TBtools: an integrative toolkit developed for interactive analyses of big biological data. Molecular Plant.

[41] (2002). PlantCARE, a database of plant *cis*-acting regulatory elements and a portal to tools for in silico analysis of promoter sequences. Nucleic Acids Research.

[42] (2017). PlantTFDB 4.0: toward a central hub for transcription factors and regulatory interactions in plants. Nucleic Acids Research.

[43] (2018). Selection and validation of reference genes for mRNA expression by quantitative real-time PCR analysis in *Neolamarckia cadamba*. Scientific Reports.

[44] (2021). Divergent camptothecin biosynthetic pathway in *Ophiorrhiza pumila*. BMC Biology.

[45] (2013). Virus-induced gene silencing identifies *Catharanthus roseus* 7-deoxyloganic acid-7-hydroxylase, a step in iridoid and monoterpene indole alkaloid biosynthesis. The Plant Journal.

[46] (2022). *FLOWERING LOCUS T* paralogs control the annual growth cycle in *Populus* trees. Current Biology.

[47] (2006). Evolution of *Arabidopsis* microRNA families through duplication events. Genome Research.

[48] (2018). Loganic acid methyltransferase: insights into the specificity of methylation on an iridoid glycoside. ChemBioChem.

[49] (2023). OpNAC1 transcription factor regulates the biosynthesis of the anticancer drug camptothecin by targeting loganic acid *O*-methyltransferase in *Ophiorrhiza pumila*. Journal of Integrative Plant Biology.

[50] (2015). Enhancement of artemisinin content and relative expression of genes of artemisinin biosynthesis in *Artemisia annua* by exogenous MeJA treatment. Plant Growth Regulation.

[51] (2018). Effects of methyl jasmonate on metabolism of topical alkaloids and expression of relate genes in *Atropa belladonna*. China Journal of Chinese Materia Medica.

[52] (2018). Metabolomics analysis reveals that ethylene and methyl jasmonate regulate different branch pathways to promote the accumulation of terpenoid indole alkaloids in *Catharanthus roseus*. Journal of Natural Products.

[53] (2022). Integrative analysis of elicitor-induced camptothecin biosynthesis in *Camptotheca acuminata* plantlets through a combined omics approach. Frontiers in Plant Science.

[54] (2019). Comparative transcriptomic analysis reveal the regulation mechanism underlying MeJA-induced accumulation of alkaloids in *Dendrobium officinale*. Journal of Plant Research.

[55] (2015). Transcriptome profiling of alkaloid biosynthesis in elicitor induced Opium Poppy. Plant Molecular Biology Reporter.

[56] (2018). Biochemical characterization in Norway spruce (*Picea abies*) of SABATH methyltransferases that methylate phytohormones. Phytochemistry.

[57] (2022). Genome-wide analysis and characterization of *SABATH* gene family in *Phaseolus vulgaris* genotypes subject to melatonin under drought and salinity stresses. Plant Molecular Biology Reporter.

[58] (2008). Structural, biochemical, and phylogenetic analyses suggest that indole-3-acetic acid methyltransferase is an evolutionarily ancient member of the SABATH family. Plant Physiology.

