# Supplementary material for: Genome-wide identification and expression pattern analysis of the SABATH gene family in Neolamarckia cadamba
Source: For Res (Fayettev). 2023 May 29;3:13. doi: 10.48130/FR-2023-0013 (PMC11524262; doi:10.48130/FR-2023-0013)
Supplement: Supplementary file 1 — Supplementary data to this article can be found online. [file FR-2023-0013-S1.zip › 10.48130_FR-2023-0013-Suppl-FigureS3.pdf]

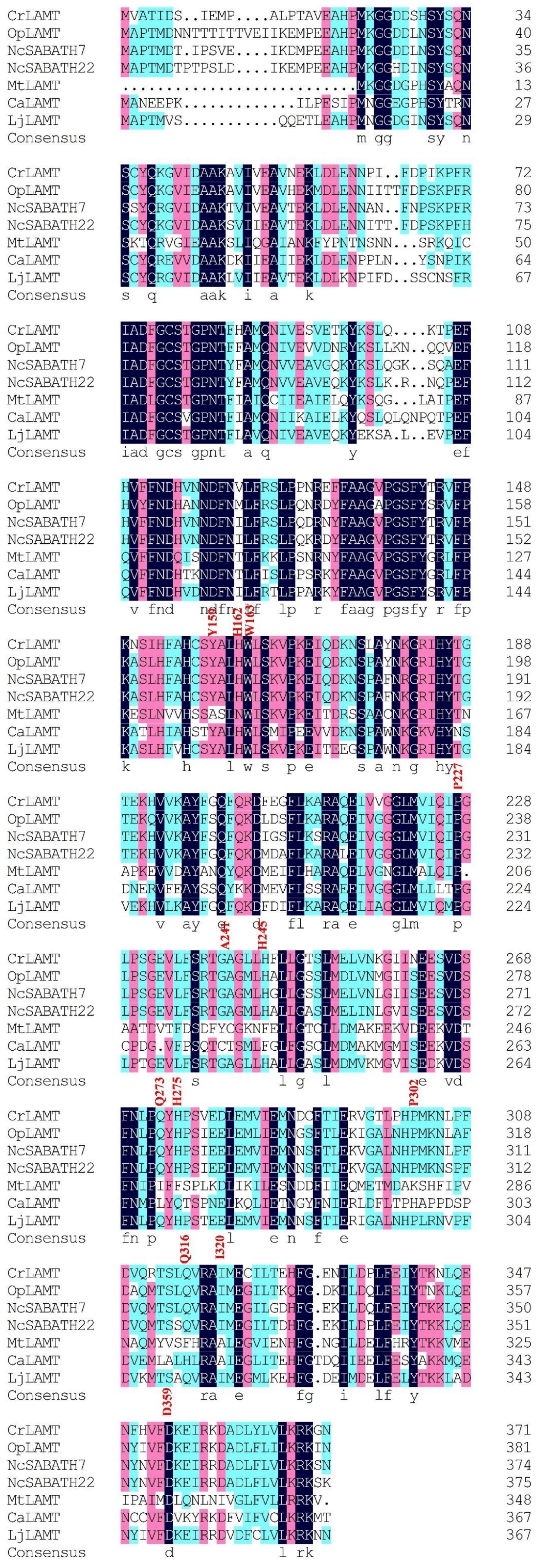

Supplementary Figure S3. Alignment of amino acid sequences of NcSABATH7/22 with LAMT from the other five species. Mt, *Medicago truncatula*; Lj, *Lonicera japonica*, Ca, *Camptotheca acuminata*. Active sites were marked in red.
