# Supplementary material for: Genome-wide identification and expression pattern analysis of the SABATH gene family in Neolamarckia cadamba
Source: For Res (Fayettev). 2023 May 29;3:13. doi: 10.48130/FR-2023-0013 (PMC11524262; doi:10.48130/FR-2023-0013)
Supplement: Supplementary file 1 — Supplementary data to this article can be found online. [file FR-2023-0013-S1.zip › 10.48130_FR-2023-0013-Suppl-FigureS4.pdf]

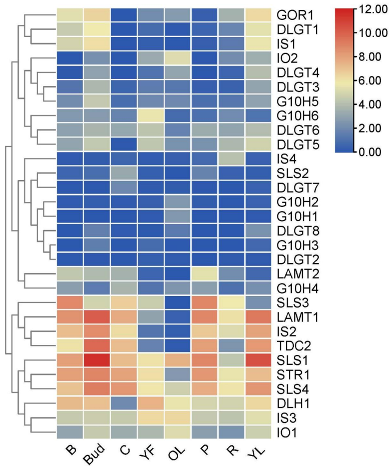

Supplementary Figure S4. Co-expression analysis of *NcSABATH7*/22 and genes related to cadambine biosynthesis. DLH, deoxyloganic acid hydroxylase; DLGT, 7-deoxyloganetic acid UDP-glucosyltransferase; G10H, Geraniol-10-hydroxylase; GOR, 8-hydroxygeraniol oxidoreductase; IO, iridoid oxidase; IS, iridoid synthase; LAMT, loganic acid methyltransferase; SLS, Secologanin synthase; STR, strictosidine synthase; TDC, tryptophan decarboxylase. Bark (B), cambium (C), bud (Bud), young fruit (YF), old leaves (OL), phloem (P), root (R), and young leaves (YL). The color scale represents relative expression levels from high (red color) to low (blue color).
