# Supplementary material for: Genome-wide identification and expression pattern analysis of the SABATH gene family in Neolamarckia cadamba
Source: For Res (Fayettev). 2023 May 29;3:13. doi: 10.48130/FR-2023-0013 (PMC11524262; doi:10.48130/FR-2023-0013)
Supplement: Supplementary file 1 — Supplementary data to this article can be found online. [file FR-2023-0013-S1.zip › 10.48130_FR-2023-0013-Suppl-TableS1.pdf]

Table S1. The accession numbers of the SABATH proteins used for ML phylogenetic tree construction from different species.

Species	Gene ID/Name	Accession no.	Database
Atropa belladonna	AbSAMT1	AB049752.1	NCBI
Aspergillus clavatus	AcSABATH1	XP_001270502	NCBI
Antirrhinum majus	AmBAMT	AF198492	NCBI
	AmSAMT	AF515284	NCBI
Arabidopsis thaliana	AT1G15125	D'Auria et al. 2003	TAIR
	AT1G19640(AtJMT)	D'Auria et al. 2003	TAIR
	AT1G66690(AtPXMT2)	D'Auria et al. 2003	TAIR
	AT1G66700(AtPXMT1)	D'Auria et al. 2003	TAIR
	AT1G66720(AtPXMT3)	D'Auria et al. 2003	TAIR
	AT1G68040	D'Auria et al. 2003	TAIR
	AT2G14060	D'Auria et al. 2003	TAIR
	AT3G11480(AtBSMT1)	D'Auria et al. 2003	TAIR
	AT3G21950	D'Auria et al. 2003	TAIR
	AT3G44840	D'Auria et al. 2003	TAIR
	AT3G44860(AtFAMT)	D'Auria et al. 2003	TAIR
	AT3G44870	D'Auria et al. 2003	TAIR
	AT4G26420(AtGAMT1)	D'Auria et al. 2003	TAIR
	AT4G36470(AtCLAMT)	D'Auria et al. 2003	TAIR
	AT5G04370(AtNAMT1)	D'Auria et al. 2003	TAIR
	AT5G04380	D'Auria et al. 2003	TAIR
	AT5G37970	D'Auria et al. 2003	TAIR
	AT5G37990	D'Auria et al. 2003	TAIR
	AT5G38020	D'Auria et al. 2003	TAIR
	AT5G38100	D'Auria et al. 2003	TAIR
	AT5G38780	D'Auria et al. 2003	TAIR
	AT5G55250(AtIAMT1)	D'Auria et al. 2003	TAIR
	AT5G56300(AtGAMT2)	D'Auria et al. 2003	TAIR
	AT5G66430	D'Auria et al. 2003	TAIR
Coffea arabica	CaCCS1	Q8H0D3	NCBI
	CaXMT1	AB048793	NCBI
	CaDXMT1	AB084125	NCBI
Clarkia breweri	CbSAMT	AF133053	NCBI
Coffea canephora	CcXMT	ABD90685.1	NCBI
Coffea canephora	CcDXMT	ABD90686.1	NCBI
Catharanthus roseus	CrLAMT	EU057974	NCBI
Camellia sinensis	CsSAMT	MG459470	NCBI
	CsTCS1	AB031280	NCBI
Coccomyxa subellipsoidea	CosSABATH1	XP_005646815	NCBI
Nicotiana suaveolens	NsBSMT	AJ628349	NCBI
	NsSAMT	ACZ55216	NCBI
Ocimum basilicum	ObCCMT1	ABV91100	NCBI
	ObCCMT2	ABV91101	NCBI
	ObCCMT3	ABV91102	NCBI
Ophiorrhiza pumila	OpLAMT	MT942677	NCBI
Oryza sativa	Os01g0700300	Bin Wang,2019	The Rice Annotation Project (RAP) Database
	Os01g0701700/OsSAMT	Bin Wang,2020	The Rice Annotation Project (RAP) Database
	Os02g0719600/OsBSMT1	Bin Wang,2021	The Rice Annotation Project (RAP) Database
	Os04g0665200/OsIAMT1	Bin Wang,2022	The Rice Annotation Project (RAP) Database
	Os05g0102000/OsJMT	Bin Wang,2023	The Rice Annotation Project (RAP) Database
	Os06g0241400	Bin Wang,2024	The Rice Annotation Project (RAP) Database
	Os06g0242000/OsBSMT2	Bin Wang,2025	The Rice Annotation Project (RAP) Database
	Os06g0242900	Bin Wang,2026	The Rice Annotation Project (RAP) Database
	Os06g0243300	Bin Wang,2027	The Rice Annotation Project (RAP) Database
	Os06g0244000	Bin Wang,2028	The Rice Annotation Project (RAP) Database
	Os06g0311800	Bin Wang,2029	The Rice Annotation Project (RAP) Database
	Os06g0313320	Bin Wang,2030	The Rice Annotation Project (RAP) Database
	Os06g0313440	Bin Wang,2031	The Rice Annotation Project (RAP) Database
	Os06g0314600	Bin Wang,2032	The Rice Annotation Project (RAP) Database
	Os06g0315000	Bin Wang,2033	The Rice Annotation Project (RAP) Database
	Os06g0315300	Bin Wang,2034	The Rice Annotation Project (RAP) Database
	Os06g0323100	Bin Wang,2035	The Rice Annotation Project (RAP) Database
	Os06g0329900	Bin Wang,2036	The Rice Annotation Project (RAP) Database
	Os11g0256600	Bin Wang,2037	The Rice Annotation Project (RAP) Database

	Os11g0257700	Bin Wang,2038	The Rice Annotation Project (RAP) Database
	Os11g0259700/OsBSMT3	Bin Wang,2039	The Rice Annotation Project (RAP) Database
Picea abies	PaJMT1	MA_128083g0020	ConGenIE
	PaJMT2	MA_9561g0010	ConGenIE
	PaJMT3	MA_10259819g0010	ConGenIE
	PaSAMT	MA_3356g0010	ConGenIE
	PaSABATH3	MA_10433097g0010	ConGenIE
	PaSABATH6	MA_10435381g0020	ConGenIE
	PaSABATH7	MA_48038g0020	ConGenIE
	PaSABATH8	MA_13253g0010	ConGenIE
	PaSABATH9	MA_25646g0010	ConGenIE
	PaIAMT	MA_98251g0010	ConGenIE
Picea glauca	PgSABATH1	GT739878	NCBI
	PgSABATH2	BT103017	NCBI
	PgSABATH3	BT101896	NCBI
	PgSABATH4	BT112158	NCBI
	PgSABATH5	BT112171	NCBI
	PgSABATH6	BT103249	NCBI
	PgSABATH7	BT108086	NCBI
	PgSABATH8	BT106072	NCBI
Petunia×hybrida	PhBSMT1	AY233465	NCBI
	PhBSMT2	AY233466	NCBI
Physcomitrella patens	PpSABATH1	Phpat.003G094200	Phytozome
	PpSABATH2	Phpat.016G083300	Phytozome
	PpSABATH3	Phpat.003G076100	Phytozome
	PpSABATH4	Phpat.005G011900	Phytozome
Populus trichocarpa	PtSABATH1(PtIAMT)	KU758927	NCBI
	PtSABATH2	KU758928	NCBI
	PtSABATH3(PtJMT)	KU758929	NCBI
	PtSABATH4(PtSAMT)	KU758930	NCBI
	PtSABATH5	KU758931	NCBI
	PtSABATH6	KU758932	NCBI
	PtSABATH7	KU758933	NCBI
	PtSABATH8	KU758934	NCBI
	PtSABATH9	KU758935	NCBI
	PtSABATH10	KU758936	NCBI
	PtSABATH11	KU758937	NCBI
	PtSABATH12	KU758938	NCBI
	PtSABATH13	KU758939	NCBI
	PtSABATH14	KU758940	NCBI
	PtSABATH15	KU758941	NCBI
	PtSABATH16	KU758942	NCBI
	PtSABATH17	KU758943	NCBI
	PtSABATH18	KU758944	NCBI
	PtSABATH19	KU758945	NCBI
	PtSABATH20	KU758946	NCBI
	PtSABATH21	KU758947	NCBI
	PtSABATH22	KU758948	NCBI
	PtSABATH23	KU758949	NCBI
	PtSABATH24(PtBAMT)	KU758950	NCBI
	PtSABATH25	KU758951	NCBI
	PtSABATH26	KU758952	NCBI
	PtSABATH27	KU758953	NCBI
	PtSABATH28	KU758954	NCBI
Stephanotis floribunda	SfSAMT	AJ308570	NCBI
Selaginella moellendorffii	SmSABATH1	65922	Phytozome
	SmSABATH2	24414	Phytozome
	SmSABATH3	439767	Phytozome
	SmSABATH4	109905	Phytozome
	SmSABATH5	75845	Phytozome
	SmSABATH6	65870	Phytozome
Zea mays	ZmAAMT1	HM242244	NCBI
	ZmAAMT2	HM242246	NCBI
	ZmAAMT3	HM242247	NCBI
Nicotiana gossei	NgNAMT	ACZ55224	NCBI
