# Supplementary material for: Genome-wide identification and expression pattern analysis of the SABATH gene family in Neolamarckia cadamba
Source: For Res (Fayettev). 2023 May 29;3:13. doi: 10.48130/FR-2023-0013 (PMC11524262; doi:10.48130/FR-2023-0013)
Supplement: Supplementary file 1 — Supplementary data to this article can be found online. [file FR-2023-0013-S1.zip › 10.48130_FR-2023-0013-Suppl-TableS2.pdf]

Table S2. Transcriptomic data of tissue expression of *NcSABATH* genes

GeneID	Gene name	Bark	Bud	Cambium	Young fruit	Old leaf	Phloem	Root	Young leaf	Primary phloem	Transitional phloem	Secondary phloem	Transitional cambium	Secondary cambium	Primary xylem	Transitional xylem	Secondary xylem
evm.model.Contig1.15	NcSABATH23	0	0.0935573	0.0498136	2.7267	13.7197	0	0	0.16097	0	0.839236	0	0	0	1.50436	1.69939	0.697417
evm.model.Contig139.120	NcSABATH20	0.247175	2.70718	0	10.0497	2.81367	0.965741	1.88874	3.10977	6.39988	10.0578	4.40367	1.25117	6.20754	2.21827	1.12301	0
evm.model.Contig139.217	NcSABATH21	0.0665908	0.433577	2.20708	0.0287773	0.302007	0.414845	0.386852	0.886041	0	0	4.55814	15.7366	0.66159	2.05308	0.0478739	0
evm.model.Contig154.585	NcSABATH14	0	0	0	0	0	0	0	0.0626258	0	0	0.144002	0.02614	0	0	0.163391	0
evm.model.Contig21.35	NcSABATH11	18.3442	141.667	14.7642	4.6096	2.732	44.9919	1.31892	51.9106	8.24274	11.8898	14.924	23.2163	13.9758	1.80054	0.607376	1.05382
evm.model.Contig267.36	NcSABATH7	364.082	1109.4	208.973	6.85033	0.286419	452.946	68.3022	610.093	64.836	540.161	586.3	165.936	995.099	34.509	1.27109	4.59071
evm.model.Contig371.14	NcSABATH17	0	0.422456	0	2.59136	0.0525601	0	0	0.200354	0	0	0	0	0	0	0	0
evm.model.Contig371.16	NcSABATH18	0.0321565	0	0.0319945	0.204936	0.115504	0	0	0.0178652	0	0.0605876	0	0	0	0	0	0
evm.model.Contig394.265	NcSABATH3	0	4.29368	0	0.763198	5.19584	0.0621061	0.894332	4.79042	0	0	0.0183542	0	0	4.44021	74.6409	30.3993
evm.model.Contig437.21	NcSABATH16	0.0130701	805.984	0.0296873	23.598	0	1.95027	0.10445	104.177	3.54654	6.99367	0	0	0.0117431	0	0	0
evm.model.Contig45.442	NcSABATH12	0	1.7276	0	9.70268	0.0212415	0	1.95601	1.56501	0	0	0	0	0.191319	3.359	75.6829	33.6522
evm.model.Contig480.228	NcSABATH8	0.242721	0.106704	3.64946	1.52711	0.0486054	0.353238	0.157704	0.622619	0	0.836381	0	8.59893	0.0281158	0	0	0
evm.model.Contig481.103	NcSABATH6	0	0	0	0	0	0	0	0	0	0	0	0	0	0	0	0
evm.model.Contig52.38	NcSABATH4	0	0	0	0	0	0	0	0	0	0.192086	0	0	0	0	0	0
evm.model.Contig52.39	NcSABATH5	0.0117961	0	0	0	0	0.0723316	0.0125315	0	0	0	0	0	0	0	0	0
evm.model.Contig54.5	NcSABATH2	0.112945	0.0349776	0	0	0	0.653218	0	0.0389184	0.102082	0.0866191	0	0	0	0	0	0
evm.model.Contig555.236	NcSABATH15	0	0.268731	0	7.31661	4.96659	0	0	1.7264	0	1.58457	0.541293	0	4.32324	0	0	0
evm.model.Contig625.59	NcSABATH22	20.2652	13.856	10.8884	0.43027	0	42.4804	3.35553	0.576567	44.1482	74.911	63.7789	20.5904	32.6498	3.8871	0	0.0165024
evm.model.Contig66.900	NcSABATH13	9.06785	2.46432	1.12314	8.27602	0.680027	2.04165	0.736109	1.15467	2.66956	0.0214191	0	0	0	0	0	0.265183
evm.model.Contig69.50	NcSABATH10	0	372.882	0	2.7644	51.9225	1.4523	0.162745	76.8093	0	0.0761366	0	0	0	0	0	0
evm.model.Contig69.51	NcSABATH9	0	96.2707	0	1.08915	6.28615	0.44686	0.0192314	16.5102	0	0.0126894	0	0	0	0	0	0
evm.model.Contig81.1046	NcSABATH1	31.6612	2.09907	2.89155	0.251612	0.102068	31.0509	1.46268	2.52565	82.1743	45.7445	25.7	12.8384	10.3873	2.04469	0.495148	0.0114178
evm.model.Contig892.11	NcSABATH19	0.0621547	0.0134502	0.127888	2.85106	9.62589	0.0457158	0	0.102856	0	0	0	0	0	0	0.0234971	0
