# Supplementary material for: Genome-wide identification and expression pattern analysis of the SABATH gene family in Neolamarckia cadamba
Source: For Res (Fayettev). 2023 May 29;3:13. doi: 10.48130/FR-2023-0013 (PMC11524262; doi:10.48130/FR-2023-0013)
Supplement: Supplementary file 1 — Supplementary data to this article can be found online. [file FR-2023-0013-S1.zip › 10.48130_FR-2023-0013-Suppl-TableS3.pdf]

Table S3. Primers used for quantitative real-time PCR

Gene name	Forward Primer	Reverse Primer
SAMDC	GGGACCAACTGGATGAAA	TGTAAGGGAAGACAAAGAG
NcSABATH11	CAGCTGCCTGGAATGAAGGA	GCGGAGTGGAGTCACAATCA
NcSABATH3	CTACGCAACATCCCAGGAGG	TCCAAAATGGTGGGCGATCA
NcSABATH16	AATTGGTAACTGGTGGCCGG	GGCCCTCAGAAACCATGTCA
NcSABATH21	TGGCCGATTTGGGATGTTCT	ATTCCGGCAGCTCATCGTAG
NcSABATH8	CGTCTCTTCCTCGCCAGATC	TTCCTGTAGGCGTTTGCTGT
NcSABATH1	TGATGACAACCAAGGACCCG	TGGTCCGTATACCGCTCTCA
NcSABATH7	GCCTTTCCGCATTGCTGATT	GCCTGGCTTTTTCCTTGCAG
NcSABATH22	TCCAGAAAGACATGGACGCA	CACTCGGAAGGCCAGGAATT
NcSTR1	TGGTCTTGGTGTGGTTGGAC	GGTCCACGGCTAAGGCATAA
