# Supplementary material for: Genome-wide identification and expression pattern analysis of the SABATH gene family in Neolamarckia cadamba
Source: For Res (Fayettev). 2023 May 29;3:13. doi: 10.48130/FR-2023-0013 (PMC11524262; doi:10.48130/FR-2023-0013)
Supplement: Supplementary file 1 — Supplementary data to this article can be found online. [file FR-2023-0013-S1.zip › 10.48130_FR-2023-0013-Suppl-TableS4.pdf]

Table S4 Classification statistics of *NcSABATHs* promoter cis-elements

Gene ID	Cis-elements	Sequence	Position	Type
evm.model.Contig1.15	chs-CMA2a	TCACTTGA	397	light-responsive elements
evm.model.Contig1.15	Box III	atCATTTTCACt	1032	Site-binding related elements
evm.model.Contig1.15	TATA-box	TACATAAA	125	promoter-related elements
evm.model.Contig1.15	TATA-box	ATTATA	151	promoter-related elements
evm.model.Contig1.15	TATA-box	TATAA	152	promoter-related elements
evm.model.Contig1.15	TATA-box	TATA	153	promoter-related elements
evm.model.Contig1.15	TATA-box	TACAAAA	237	promoter-related elements
evm.model.Contig1.15	TATA-box	TATATA	303	promoter-related elements
evm.model.Contig1.15	TATA-box	ATATAA	304	promoter-related elements
evm.model.Contig1.15	TATA-box	TATA	305	promoter-related elements
evm.model.Contig1.15	TATA-box	TATAA	352	promoter-related elements
evm.model.Contig1.15	TATA-box	TATA	353	promoter-related elements
evm.model.Contig1.15	TATA-box	TATA	379	promoter-related elements
evm.model.Contig1.15	TATA-box	TATAA	627	promoter-related elements
evm.model.Contig1.15	TATA-box	TATA	628	promoter-related elements
evm.model.Contig1.15	TATA-box	ATATAA	671	promoter-related elements
evm.model.Contig1.15	TATA-box	TATA	672	promoter-related elements
evm.model.Contig1.15	TATA-box	ATATAA	1381	promoter-related elements
evm.model.Contig1.15	TATA-box	TATA	1382	promoter-related elements
evm.model.Contig1.15	TATA-box	TAAAGATT	1429	promoter-related elements
evm.model.Contig1.15	TATA-box	TATAAA	1433	promoter-related elements
evm.model.Contig1.15	TATA-box	TATAA	1434	promoter-related elements
evm.model.Contig1.15	TATA-box	TATA	1435	promoter-related elements
evm.model.Contig1.15	TATA-box	TACATAAA	1872	promoter-related elements
evm.model.Contig1.15	TATA-box	TATATA	1901	promoter-related elements
evm.model.Contig1.15	TATA-box	ATATAT	1902	promoter-related elements
evm.model.Contig1.15	TATA-box	TATATA	1903	promoter-related elements
evm.model.Contig1.15	TATA-box	ATATAA	1904	promoter-related elements
evm.model.Contig1.15	TATA-box	TATA	1905	promoter-related elements
evm.model.Contig1.15	MYB-like sequence	TAACCA	624	Site-binding related elements
evm.model.Contig1.15	MYB-like sequence	TAACCA	770	Site-binding related elements
evm.model.Contig1.15	MYB-like sequence	TAACCA	1106	Site-binding related elements
evm.model.Contig1.15	CGTCA-motif	CGTCA	431	homone-responsive elements
evm.model.Contig1.15	CGTCA-motif	CGTCA	1504	homone-responsive elements
evm.model.Contig1.15	G-box	TACGTG	381	light-responsive elements
evm.model.Contig1.15	G-box	CACGTG	749	light-responsive elements
evm.model.Contig1.15	G-box	CACGTG	760	light-responsive elements
evm.model.Contig1.15	G-box	CACGTG	1127	light-responsive elements
evm.model.Contig1.15	Myb	TAACTG	442	Site-binding related elements
evm.model.Contig1.15	Myb	CAACTG	680	Site-binding related elements
evm.model.Contig1.15	Myb	TAACTG	1699	Site-binding related elements
evm.model.Contig1.15	STRE	AGGGG	498	environmental stress-related elements
evm.model.Contig1.15	ABRE	ACGTG	382	homone-responsive elements
evm.model.Contig1.15	ABRE	CACGTG	749	homone-responsive elements
evm.model.Contig1.15	ABRE	ACGTG	750	homone-responsive elements
evm.model.Contig1.15	ABRE	CACGTG	760	homone-responsive elements
evm.model.Contig1.15	ABRE	ACGTG	761	homone-responsive elements
evm.model.Contig1.15	ABRE	CACGTG	1127	homone-responsive elements
evm.model.Contig1.15	ABRE	ACGTG	1128	homone-responsive elements
evm.model.Contig1.15	G-Box	CACGTG	749	light-responsive elements
evm.model.Contig1.15	G-Box	CACGTG	760	light-responsive elements
evm.model.Contig1.15	G-Box	CACGTG	1127	light-responsive elements
evm.model.Contig1.15	WRE3	CCACCT	70	environmental stress-related elements
evm.model.Contig1.15	ARE	AAACCA	130	environmental stress-related elements
evm.model.Contig1.15	ARE	AAACCA	234	environmental stress-related elements
evm.model.Contig1.15	ARE	AAACCA	1625	environmental stress-related elements
evm.model.Contig1.15	ARE	AAACCA	1639	environmental stress-related elements
evm.model.Contig1.15	ATCT-motif	AATCTAATCC	1554	light-responsive elements
evm.model.Contig1.15	TCT-motif	TCTTAC	425	light-responsive elements
evm.model.Contig1.15	TCT-motif	TCTTAC	1144	light-responsive elements
evm.model.Contig1.15	MYC	CATTTG	182	Site-binding related elements
evm.model.Contig1.15	MYC	CAATTG	1049	Site-binding related elements
evm.model.Contig1.15	MYC	CATGTG	1116	Site-binding related elements
evm.model.Contig1.15	MYC	CAATTG	1248	Site-binding related elements
evm.model.Contig1.15	MYC	CATTTG	1340	Site-binding related elements
evm.model.Contig1.15	MBS	CAACTG	680	Site-binding related elements
evm.model.Contig1.15	TCA-element	CCATCTTTTT	66	homone-responsive elements
evm.model.Contig1.15	TGACG-motif	TGACG	431	homone-responsive elements
evm.model.Contig1.15	TGACG-motif	TGACG	1504	homone-responsive elements
evm.model.Contig1.15	MYB	CAACCA	367	Site-binding related elements
evm.model.Contig1.15	MYB	TAACCA	624	Site-binding related elements
evm.model.Contig1.15	MYB	TAACCA	770	Site-binding related elements
evm.model.Contig1.15	MYB	TAACCA	1106	Site-binding related elements
evm.model.Contig1.15	LTR	CCGAAA	1931	environmental stress-related elements
evm.model.Contig1.15	CAAT-box	CAAT	39	promoter-related elements
evm.model.Contig1.15	CAAT-box	CAAAT	182	promoter-related elements

evm.model.Contig1.15	CAAT-box	CAAT	192	promoter-related elements
evm.model.Contig1.15	CAAT-box	CAAAT	243	promoter-related elements
evm.model.Contig1.15	CAAT-box	CAAT	324	promoter-related elements
evm.model.Contig1.15	CAAT-box	CAAT	336	promoter-related elements
evm.model.Contig1.15	CAAT-box	CAAAT	410	promoter-related elements
evm.model.Contig1.15	CAAT-box	CAAT	503	promoter-related elements
evm.model.Contig1.15	CAAT-box	CAAT	521	promoter-related elements
evm.model.Contig1.15	CAAT-box	CCAAT	551	promoter-related elements
evm.model.Contig1.15	CAAT-box	CAAAT	586	promoter-related elements
evm.model.Contig1.15	CAAT-box	CAAAT	607	promoter-related elements
evm.model.Contig1.15	CAAT-box	CAAAT	618	promoter-related elements
evm.model.Contig1.15	CAAT-box	CAAAT	621	promoter-related elements
evm.model.Contig1.15	CAAT-box	CAAT	631	promoter-related elements
evm.model.Contig1.15	CAAT-box	CAAAT	668	promoter-related elements
evm.model.Contig1.15	CAAT-box	CAAT	713	promoter-related elements
evm.model.Contig1.15	CAAT-box	CAAT	719	promoter-related elements
evm.model.Contig1.15	CAAT-box	CAAAT	728	promoter-related elements
evm.model.Contig1.15	CAAT-box	CCAAT	766	promoter-related elements
evm.model.Contig1.15	CAAT-box	CAAT	767	promoter-related elements
evm.model.Contig1.15	CAAT-box	CAAAT	774	promoter-related elements
evm.model.Contig1.15	CAAT-box	CAAT	833	promoter-related elements
evm.model.Contig1.15	CAAT-box	CAAAT	839	promoter-related elements
evm.model.Contig1.15	CAAT-box	CAAT	964	promoter-related elements
evm.model.Contig1.15	CAAT-box	CAAAT	979	promoter-related elements
evm.model.Contig1.15	CAAT-box	CAAAT	1021	promoter-related elements
evm.model.Contig1.15	CAAT-box	CAAT	1049	promoter-related elements
evm.model.Contig1.15	CAAT-box	CAAT	1051	promoter-related elements
evm.model.Contig1.15	CAAT-box	CAAAT	1055	promoter-related elements
evm.model.Contig1.15	CAAT-box	CAAAT	1103	promoter-related elements
evm.model.Contig1.15	CAAT-box	CAAT	1190	promoter-related elements
evm.model.Contig1.15	CAAT-box	CAAT	1248	promoter-related elements
evm.model.Contig1.15	CAAT-box	CAAT	1250	promoter-related elements
evm.model.Contig1.15	CAAT-box	CAAAT	1341	promoter-related elements
evm.model.Contig1.15	CAAT-box	CCAAT	1417	promoter-related elements
evm.model.Contig1.15	CAAT-box	CAAT	1418	promoter-related elements
evm.model.Contig1.15	CAAT-box	CAAT	1459	promoter-related elements
evm.model.Contig1.15	CAAT-box	CAAT	1475	promoter-related elements
evm.model.Contig1.15	CAAT-box	CAAT	1509	promoter-related elements
evm.model.Contig1.15	CAAT-box	CAAAT	1525	promoter-related elements
evm.model.Contig1.15	CAAT-box	CAAAT	1561	promoter-related elements
evm.model.Contig1.15	CAAT-box	CAAT	1572	promoter-related elements
evm.model.Contig1.15	CAAT-box	CAAAT	1661	promoter-related elements
evm.model.Contig1.15	CAAT-box	CAAT	1705	promoter-related elements
evm.model.Contig1.15	CAAT-box	CAAT	1717	promoter-related elements
evm.model.Contig1.15	CAAT-box	CCAAT	1770	promoter-related elements
evm.model.Contig1.15	CAAT-box	CCAAT	1837	promoter-related elements
evm.model.Contig1.15	CAAT-box	CAAT	1838	promoter-related elements
evm.model.Contig1.15	CAAT-box	CAAAT	1840	promoter-related elements
evm.model.Contig1.15	CAAT-box	CAAT	1868	promoter-related elements
evm.model.Contig1.15	CAAT-box	CAAT	1896	promoter-related elements
evm.model.Contig1.15	Box 4	ATTAAT	1043	light-responsive elements
evm.model.Contig139.120	Myb-binding site	CAACAG	1059	Site-binding related elements
evm.model.Contig139.120	G-box	TACGTG	453	light-responsive elements
evm.model.Contig139.120	G-box	TACGTG	851	light-responsive elements
evm.model.Contig139.120	G-box	ACACGTGT	872	light-responsive elements
evm.model.Contig139.120	G-box	CACGTG	873	light-responsive elements
evm.model.Contig139.120	G-box	TACGTG	1164	light-responsive elements
evm.model.Contig139.120	Myb	CAACTG	1605	Site-binding related elements
evm.model.Contig139.120	Myb	CAACTG	1687	Site-binding related elements
evm.model.Contig139.120	GARE-motif	TCTGTTG	1058	homone-responsive elements
evm.model.Contig139.120	TATA-box	TATATAA	1	promoter-related elements
evm.model.Contig139.120	TATA-box	TATATA	2	promoter-related elements
evm.model.Contig139.120	TATA-box	TATA	4	promoter-related elements
evm.model.Contig139.120	TATA-box	TATA	12	promoter-related elements
evm.model.Contig139.120	TATA-box	TATATA	24	promoter-related elements
evm.model.Contig139.120	TATA-box	ATATAA	25	promoter-related elements
evm.model.Contig139.120	TATA-box	TATA	26	promoter-related elements
evm.model.Contig139.120	TATA-box	ATTATA	32	promoter-related elements
evm.model.Contig139.120	TATA-box	TATAA	33	promoter-related elements
evm.model.Contig139.120	TATA-box	TATA	34	promoter-related elements
evm.model.Contig139.120	TATA-box	taTATAAAtc	41	promoter-related elements
evm.model.Contig139.120	TATA-box	TATAAAT	42	promoter-related elements
evm.model.Contig139.120	TATA-box	TATAAA	43	promoter-related elements
evm.model.Contig139.120	TATA-box	TATATAA	44	promoter-related elements
evm.model.Contig139.120	TATA-box	TATATA	45	promoter-related elements
evm.model.Contig139.120	TATA-box	ATATAT	46	promoter-related elements
evm.model.Contig139.120	TATA-box	TATA	47	promoter-related elements
evm.model.Contig139.120	TATA-box	TATACA	51	promoter-related elements

evm.model.Contig139.120	TATA-box	TATA	53	promoter-related elements
evm.model.Contig139.120	TATA-box	ATATAT	57	promoter-related elements
evm.model.Contig139.120	TATA-box	TATATA	58	promoter-related elements
evm.model.Contig139.120	TATA-box	ATATAT	59	promoter-related elements
evm.model.Contig139.120	TATA-box	TATATA	60	promoter-related elements
evm.model.Contig139.120	TATA-box	TATA	62	promoter-related elements
evm.model.Contig139.120	TATA-box	TATAA	71	promoter-related elements
evm.model.Contig139.120	TATA-box	TATA	72	promoter-related elements
evm.model.Contig139.120	TATA-box	TATA	81	promoter-related elements
evm.model.Contig139.120	TATA-box	ATTATA	85	promoter-related elements
evm.model.Contig139.120	TATA-box	TATAA	86	promoter-related elements
evm.model.Contig139.120	TATA-box	TATA	87	promoter-related elements
evm.model.Contig139.120	TATA-box	ATTATA	91	promoter-related elements
evm.model.Contig139.120	TATA-box	TATATAA	92	promoter-related elements
evm.model.Contig139.120	TATA-box	TATATA	93	promoter-related elements
evm.model.Contig139.120	TATA-box	ATATAT	94	promoter-related elements
evm.model.Contig139.120	TATA-box	TATATA	95	promoter-related elements
evm.model.Contig139.120	TATA-box	ATATAT	96	promoter-related elements
evm.model.Contig139.120	TATA-box	TATATA	97	promoter-related elements
evm.model.Contig139.120	TATA-box	ATATAA	98	promoter-related elements
evm.model.Contig139.120	TATA-box	TATA	99	promoter-related elements
evm.model.Contig139.120	TATA-box	TATATA	109	promoter-related elements
evm.model.Contig139.120	TATA-box	ATATAT	110	promoter-related elements
evm.model.Contig139.120	TATA-box	TATA	111	promoter-related elements
evm.model.Contig139.120	TATA-box	TATA	120	promoter-related elements
evm.model.Contig139.120	TATA-box	ATTATA	145	promoter-related elements
evm.model.Contig139.120	TATA-box	TATAA	146	promoter-related elements
evm.model.Contig139.120	TATA-box	TATA	147	promoter-related elements
evm.model.Contig139.120	TATA-box	ATATAT	151	promoter-related elements
evm.model.Contig139.120	TATA-box	TATA	152	promoter-related elements
evm.model.Contig139.120	TATA-box	ATATAT	173	promoter-related elements
evm.model.Contig139.120	TATA-box	TATA	174	promoter-related elements
evm.model.Contig139.120	TATA-box	ATTATA	177	promoter-related elements
evm.model.Contig139.120	TATA-box	TATATAA	178	promoter-related elements
evm.model.Contig139.120	TATA-box	TATATA	179	promoter-related elements
evm.model.Contig139.120	TATA-box	ATATAT	180	promoter-related elements
evm.model.Contig139.120	TATA-box	TATA	181	promoter-related elements
evm.model.Contig139.120	TATA-box	ATATAT	188	promoter-related elements
evm.model.Contig139.120	TATA-box	TATATA	189	promoter-related elements
evm.model.Contig139.120	TATA-box	ATATAT	190	promoter-related elements
evm.model.Contig139.120	TATA-box	TATA	191	promoter-related elements
evm.model.Contig139.120	TATA-box	TATATA	198	promoter-related elements
evm.model.Contig139.120	TATA-box	ATATAT	199	promoter-related elements
evm.model.Contig139.120	TATA-box	TATA	200	promoter-related elements
evm.model.Contig139.120	TATA-box	ATTATA	203	promoter-related elements
evm.model.Contig139.120	TATA-box	TATATAA	204	promoter-related elements
evm.model.Contig139.120	TATA-box	TATATA	205	promoter-related elements
evm.model.Contig139.120	TATA-box	ATATAT	206	promoter-related elements
evm.model.Contig139.120	TATA-box	TATATA	207	promoter-related elements
evm.model.Contig139.120	TATA-box	TATA	209	promoter-related elements
evm.model.Contig139.120	TATA-box	ATATAA	227	promoter-related elements
evm.model.Contig139.120	TATA-box	TATA	228	promoter-related elements
evm.model.Contig139.120	TATA-box	TATACA	239	promoter-related elements
evm.model.Contig139.120	TATA-box	TATA	241	promoter-related elements
evm.model.Contig139.120	TATA-box	ATATAT	246	promoter-related elements
evm.model.Contig139.120	TATA-box	TATATA	247	promoter-related elements
evm.model.Contig139.120	TATA-box	TATA	249	promoter-related elements
evm.model.Contig139.120	TATA-box	TATACA	265	promoter-related elements
evm.model.Contig139.120	TATA-box	TATA	267	promoter-related elements
evm.model.Contig139.120	TATA-box	ATTATA	276	promoter-related elements
evm.model.Contig139.120	TATA-box	TATAA	277	promoter-related elements
evm.model.Contig139.120	TATA-box	TATA	278	promoter-related elements
evm.model.Contig139.120	TATA-box	ATATAT	284	promoter-related elements
evm.model.Contig139.120	TATA-box	TATATA	285	promoter-related elements
evm.model.Contig139.120	TATA-box	ATATAT	286	promoter-related elements
evm.model.Contig139.120	TATA-box	TATATA	287	promoter-related elements
evm.model.Contig139.120	TATA-box	ATATAA	288	promoter-related elements
evm.model.Contig139.120	TATA-box	TATA	289	promoter-related elements
evm.model.Contig139.120	TATA-box	TATATA	303	promoter-related elements
evm.model.Contig139.120	TATA-box	ATATAA	304	promoter-related elements
evm.model.Contig139.120	TATA-box	TATA	305	promoter-related elements
evm.model.Contig139.120	TATA-box	ATATAT	309	promoter-related elements
evm.model.Contig139.120	TATA-box	TATATA	310	promoter-related elements
evm.model.Contig139.120	TATA-box	ATATAT	311	promoter-related elements
evm.model.Contig139.120	TATA-box	TATATA	312	promoter-related elements
evm.model.Contig139.120	TATA-box	TATA	314	promoter-related elements
evm.model.Contig139.120	TATA-box	TATAAA	323	promoter-related elements
evm.model.Contig139.120	TATA-box	TATAA	324	promoter-related elements

evm.model.Contig139.120	TATA-box	TATA	325	promoter-related elements
evm.model.Contig139.120	TATA-box	TATA	330	promoter-related elements
evm.model.Contig139.120	TATA-box	ATATAT	348	promoter-related elements
evm.model.Contig139.120	TATA-box	TATATA	349	promoter-related elements
evm.model.Contig139.120	TATA-box	ATATAT	350	promoter-related elements
evm.model.Contig139.120	TATA-box	TATATA	351	promoter-related elements
evm.model.Contig139.120	TATA-box	ATATAA	352	promoter-related elements
evm.model.Contig139.120	TATA-box	TATA	353	promoter-related elements
evm.model.Contig139.120	TATA-box	ATATAT	361	promoter-related elements
evm.model.Contig139.120	TATA-box	TATATA	362	promoter-related elements
evm.model.Contig139.120	TATA-box	ATATAT	363	promoter-related elements
evm.model.Contig139.120	TATA-box	TATATA	364	promoter-related elements
evm.model.Contig139.120	TATA-box	ATATAT	365	promoter-related elements
evm.model.Contig139.120	TATA-box	TATA	366	promoter-related elements
evm.model.Contig139.120	TATA-box	ATTATA	369	promoter-related elements
evm.model.Contig139.120	TATA-box	TATAA	370	promoter-related elements
evm.model.Contig139.120	TATA-box	TATA	371	promoter-related elements
evm.model.Contig139.120	TATA-box	ATTATA	374	promoter-related elements
evm.model.Contig139.120	TATA-box	TATATAA	375	promoter-related elements
evm.model.Contig139.120	TATA-box	TATATA	376	promoter-related elements
evm.model.Contig139.120	TATA-box	ATATAT	377	promoter-related elements
evm.model.Contig139.120	TATA-box	TATA	378	promoter-related elements
evm.model.Contig139.120	TATA-box	TATATAA	400	promoter-related elements
evm.model.Contig139.120	TATA-box	TATATA	401	promoter-related elements
evm.model.Contig139.120	TATA-box	ATATAT	402	promoter-related elements
evm.model.Contig139.120	TATA-box	TATATA	403	promoter-related elements
evm.model.Contig139.120	TATA-box	TATA	405	promoter-related elements
evm.model.Contig139.120	TATA-box	TATATA	410	promoter-related elements
evm.model.Contig139.120	TATA-box	TATA	412	promoter-related elements
evm.model.Contig139.120	TATA-box	TATAA	430	promoter-related elements
evm.model.Contig139.120	TATA-box	TATA	431	promoter-related elements
evm.model.Contig139.120	TATA-box	TATACA	472	promoter-related elements
evm.model.Contig139.120	TATA-box	TATATA	474	promoter-related elements
evm.model.Contig139.120	TATA-box	ATATAT	475	promoter-related elements
evm.model.Contig139.120	TATA-box	TATATA	476	promoter-related elements
evm.model.Contig139.120	TATA-box	ATATAT	477	promoter-related elements
evm.model.Contig139.120	TATA-box	TATATA	478	promoter-related elements
evm.model.Contig139.120	TATA-box	ATATAA	479	promoter-related elements
evm.model.Contig139.120	TATA-box	TATA	480	promoter-related elements
evm.model.Contig139.120	TATA-box	TATA	485	promoter-related elements
evm.model.Contig139.120	TATA-box	ATATAT	502	promoter-related elements
evm.model.Contig139.120	TATA-box	TATA	503	promoter-related elements
evm.model.Contig139.120	TATA-box	TATATAA	509	promoter-related elements
evm.model.Contig139.120	TATA-box	TATATA	510	promoter-related elements
evm.model.Contig139.120	TATA-box	ATATAT	511	promoter-related elements
evm.model.Contig139.120	TATA-box	TATATA	512	promoter-related elements
evm.model.Contig139.120	TATA-box	ATATAT	513	promoter-related elements
evm.model.Contig139.120	TATA-box	TATATA	514	promoter-related elements
evm.model.Contig139.120	TATA-box	ATATAA	515	promoter-related elements
evm.model.Contig139.120	TATA-box	TATA	516	promoter-related elements
evm.model.Contig139.120	TATA-box	TATA	521	promoter-related elements
evm.model.Contig139.120	TATA-box	TATACA	529	promoter-related elements
evm.model.Contig139.120	TATA-box	TATATA	531	promoter-related elements
evm.model.Contig139.120	TATA-box	ATATAT	532	promoter-related elements
evm.model.Contig139.120	TATA-box	TATATA	533	promoter-related elements
evm.model.Contig139.120	TATA-box	ATATAT	534	promoter-related elements
evm.model.Contig139.120	TATA-box	TATA	535	promoter-related elements
evm.model.Contig139.120	TATA-box	ATTATA	549	promoter-related elements
evm.model.Contig139.120	TATA-box	TATAA	550	promoter-related elements
evm.model.Contig139.120	TATA-box	TATA	551	promoter-related elements
evm.model.Contig139.120	TATA-box	ATTATA	554	promoter-related elements
evm.model.Contig139.120	TATA-box	TATAA	555	promoter-related elements
evm.model.Contig139.120	TATA-box	TATA	556	promoter-related elements
evm.model.Contig139.120	TATA-box	TATACA	560	promoter-related elements
evm.model.Contig139.120	TATA-box	TATA	562	promoter-related elements
evm.model.Contig139.120	TATA-box	TATACA	582	promoter-related elements
evm.model.Contig139.120	TATA-box	TATA	584	promoter-related elements
evm.model.Contig139.120	TATA-box	ATATAT	601	promoter-related elements
evm.model.Contig139.120	TATA-box	TATATA	602	promoter-related elements
evm.model.Contig139.120	TATA-box	TATA	604	promoter-related elements
evm.model.Contig139.120	TATA-box	ATATAT	616	promoter-related elements
evm.model.Contig139.120	TATA-box	TATATA	617	promoter-related elements
evm.model.Contig139.120	TATA-box	ATATAA	618	promoter-related elements
evm.model.Contig139.120	TATA-box	TATA	619	promoter-related elements
evm.model.Contig139.120	TATA-box	ATATAA	625	promoter-related elements
evm.model.Contig139.120	TATA-box	TATA	626	promoter-related elements
evm.model.Contig139.120	TATA-box	ATATAT	630	promoter-related elements
evm.model.Contig139.120	TATA-box	TATATA	631	promoter-related elements

evm.model.Contig139.120	TATA-box	ATATAT	632	promoter-related elements
evm.model.Contig139.120	TATA-box	TATATA	633	promoter-related elements
evm.model.Contig139.120	TATA-box	ATATAT	634	promoter-related elements
evm.model.Contig139.120	TATA-box	TATATA	635	promoter-related elements
evm.model.Contig139.120	TATA-box	ATATAT	636	promoter-related elements
evm.model.Contig139.120	TATA-box	TATATA	637	promoter-related elements
evm.model.Contig139.120	TATA-box	ATATAT	638	promoter-related elements
evm.model.Contig139.120	TATA-box	TATATA	639	promoter-related elements
evm.model.Contig139.120	TATA-box	ATATAT	640	promoter-related elements
evm.model.Contig139.120	TATA-box	TATATA	641	promoter-related elements
evm.model.Contig139.120	TATA-box	ATATAT	642	promoter-related elements
evm.model.Contig139.120	TATA-box	TATATA	643	promoter-related elements
evm.model.Contig139.120	TATA-box	ATATAT	644	promoter-related elements
evm.model.Contig139.120	TATA-box	TATATA	645	promoter-related elements
evm.model.Contig139.120	TATA-box	ATATAT	646	promoter-related elements
evm.model.Contig139.120	TATA-box	TATA	647	promoter-related elements
evm.model.Contig139.120	TATA-box	TATACA	651	promoter-related elements
evm.model.Contig139.120	TATA-box	TATATA	653	promoter-related elements
evm.model.Contig139.120	TATA-box	ATATAT	654	promoter-related elements
evm.model.Contig139.120	TATA-box	TATATA	655	promoter-related elements
evm.model.Contig139.120	TATA-box	ATATAT	656	promoter-related elements
evm.model.Contig139.120	TATA-box	TATATA	657	promoter-related elements
evm.model.Contig139.120	TATA-box	ATATAT	658	promoter-related elements
evm.model.Contig139.120	TATA-box	TATATA	659	promoter-related elements
evm.model.Contig139.120	TATA-box	ATATAT	660	promoter-related elements
evm.model.Contig139.120	TATA-box	TATA	661	promoter-related elements
evm.model.Contig139.120	TATA-box	ATTATA	664	promoter-related elements
evm.model.Contig139.120	TATA-box	TATAA	665	promoter-related elements
evm.model.Contig139.120	TATA-box	TATA	666	promoter-related elements
evm.model.Contig139.120	TATA-box	ATATAT	703	promoter-related elements
evm.model.Contig139.120	TATA-box	TATATA	704	promoter-related elements
evm.model.Contig139.120	TATA-box	ATATAT	705	promoter-related elements
evm.model.Contig139.120	TATA-box	TATATA	706	promoter-related elements
evm.model.Contig139.120	TATA-box	TATA	708	promoter-related elements
evm.model.Contig139.120	TATA-box	ATATAT	729	promoter-related elements
evm.model.Contig139.120	TATA-box	TATA	730	promoter-related elements
evm.model.Contig139.120	TATA-box	TATATAA	736	promoter-related elements
evm.model.Contig139.120	TATA-box	TATATA	737	promoter-related elements
evm.model.Contig139.120	TATA-box	ATATAT	738	promoter-related elements
evm.model.Contig139.120	TATA-box	TATATA	739	promoter-related elements
evm.model.Contig139.120	TATA-box	ATATAA	740	promoter-related elements
evm.model.Contig139.120	TATA-box	TATA	741	promoter-related elements
evm.model.Contig139.120	TATA-box	TACATAAA	746	promoter-related elements
evm.model.Contig139.120	TATA-box	TATACA	754	promoter-related elements
evm.model.Contig139.120	TATA-box	TATA	756	promoter-related elements
evm.model.Contig139.120	TATA-box	ATTATA	759	promoter-related elements
evm.model.Contig139.120	TATA-box	TATAA	760	promoter-related elements
evm.model.Contig139.120	TATA-box	TATA	761	promoter-related elements
evm.model.Contig139.120	TATA-box	ATTATA	764	promoter-related elements
evm.model.Contig139.120	TATA-box	TATATAA	765	promoter-related elements
evm.model.Contig139.120	TATA-box	TATATA	766	promoter-related elements
evm.model.Contig139.120	TATA-box	ATATAT	767	promoter-related elements
evm.model.Contig139.120	TATA-box	TATA	768	promoter-related elements
evm.model.Contig139.120	TATA-box	ATTATA	776	promoter-related elements
evm.model.Contig139.120	TATA-box	TATAA	777	promoter-related elements
evm.model.Contig139.120	TATA-box	TATA	778	promoter-related elements
evm.model.Contig139.120	TATA-box	TATAAAA	782	promoter-related elements
evm.model.Contig139.120	TATA-box	TATAAA	783	promoter-related elements
evm.model.Contig139.120	TATA-box	TATATAA	784	promoter-related elements
evm.model.Contig139.120	TATA-box	TATATA	785	promoter-related elements
evm.model.Contig139.120	TATA-box	ATATAT	786	promoter-related elements
evm.model.Contig139.120	TATA-box	TATATA	787	promoter-related elements
evm.model.Contig139.120	TATA-box	ATATAT	788	promoter-related elements
evm.model.Contig139.120	TATA-box	TATATA	789	promoter-related elements
evm.model.Contig139.120	TATA-box	ATATAT	790	promoter-related elements
evm.model.Contig139.120	TATA-box	TATATA	791	promoter-related elements
evm.model.Contig139.120	TATA-box	ATATAT	792	promoter-related elements
evm.model.Contig139.120	TATA-box	TATATA	793	promoter-related elements
evm.model.Contig139.120	TATA-box	ATATAT	794	promoter-related elements
evm.model.Contig139.120	TATA-box	TATATA	795	promoter-related elements
evm.model.Contig139.120	TATA-box	ATATAT	796	promoter-related elements
evm.model.Contig139.120	TATA-box	TATA	797	promoter-related elements
evm.model.Contig139.120	TATA-box	ATTATA	800	promoter-related elements
evm.model.Contig139.120	TATA-box	TATAA	801	promoter-related elements
evm.model.Contig139.120	TATA-box	TATA	802	promoter-related elements
evm.model.Contig139.120	TATA-box	TATAAAA	806	promoter-related elements
evm.model.Contig139.120	TATA-box	TATAAA	807	promoter-related elements
evm.model.Contig139.120	TATA-box	TATATAA	808	promoter-related elements

evm.model.Contig139.120	TATA-box	TATATA	809	promoter-related elements
evm.model.Contig139.120	TATA-box	ATATAT	810	promoter-related elements
evm.model.Contig139.120	TATA-box	TATA	811	promoter-related elements
evm.model.Contig139.120	TATA-box	TATACA	823	promoter-related elements
evm.model.Contig139.120	TATA-box	TATATA	825	promoter-related elements
evm.model.Contig139.120	TATA-box	ATATAT	826	promoter-related elements
evm.model.Contig139.120	TATA-box	TATA	827	promoter-related elements
evm.model.Contig139.120	TATA-box	TATA	845	promoter-related elements
evm.model.Contig139.120	TATA-box	TATACA	859	promoter-related elements
evm.model.Contig139.120	TATA-box	TATA	861	promoter-related elements
evm.model.Contig139.120	TATA-box	TATACA	897	promoter-related elements
evm.model.Contig139.120	TATA-box	TATATA	899	promoter-related elements
evm.model.Contig139.120	TATA-box	ATATAT	900	promoter-related elements
evm.model.Contig139.120	TATA-box	TATATA	901	promoter-related elements
evm.model.Contig139.120	TATA-box	ATATAT	902	promoter-related elements
evm.model.Contig139.120	TATA-box	TATATA	903	promoter-related elements
evm.model.Contig139.120	TATA-box	ATATAT	904	promoter-related elements
evm.model.Contig139.120	TATA-box	TATATA	905	promoter-related elements
evm.model.Contig139.120	TATA-box	TATA	907	promoter-related elements
evm.model.Contig139.120	TATA-box	taTATAAAg	1118	promoter-related elements
evm.model.Contig139.120	TATA-box	TATAAA	1119	promoter-related elements
evm.model.Contig139.120	TATA-box	TATATAA	1120	promoter-related elements
evm.model.Contig139.120	TATA-box	TATATA	1121	promoter-related elements
evm.model.Contig139.120	TATA-box	ATATAA	1122	promoter-related elements
evm.model.Contig139.120	TATA-box	TATA	1123	promoter-related elements
evm.model.Contig139.120	TATA-box	TATAAATA	1201	promoter-related elements
evm.model.Contig139.120	TATA-box	TATAAAT	1202	promoter-related elements
evm.model.Contig139.120	TATA-box	TATAAA	1203	promoter-related elements
evm.model.Contig139.120	TATA-box	TATAA	1204	promoter-related elements
evm.model.Contig139.120	TATA-box	TATA	1205	promoter-related elements
evm.model.Contig139.120	TATA-box	TATA	1262	promoter-related elements
evm.model.Contig139.120	TATA-box	TATAA	1320	promoter-related elements
evm.model.Contig139.120	TATA-box	TATA	1321	promoter-related elements
evm.model.Contig139.120	TATA-box	TATAAAA	1372	promoter-related elements
evm.model.Contig139.120	TATA-box	TATAAA	1373	promoter-related elements
evm.model.Contig139.120	TATA-box	TATAA	1374	promoter-related elements
evm.model.Contig139.120	TATA-box	TATA	1375	promoter-related elements
evm.model.Contig139.120	TATA-box	TATA	1383	promoter-related elements
evm.model.Contig139.120	TATA-box	TATAAA	1515	promoter-related elements
evm.model.Contig139.120	TATA-box	TATAA	1516	promoter-related elements
evm.model.Contig139.120	TATA-box	TATA	1517	promoter-related elements
evm.model.Contig139.120	TATA-box	ATATAA	1861	promoter-related elements
evm.model.Contig139.120	TATA-box	TATA	1862	promoter-related elements
evm.model.Contig139.120	TATA-box	TATAAAA	1879	promoter-related elements
evm.model.Contig139.120	TATA-box	TATAAA	1880	promoter-related elements
evm.model.Contig139.120	TATA-box	TATAA	1881	promoter-related elements
evm.model.Contig139.120	TATA-box	TATA	1882	promoter-related elements
evm.model.Contig139.120	GATA-motif	GATAGGA	1889	light-responsive elements
evm.model.Contig139.120	WUN-motif	TTATTACAT	542	environmental stress-related elements
evm.model.Contig139.120	MYC	CATTTG	1789	Site-binding related elements
evm.model.Contig139.120	ACE	GACACGTATG	451	light-responsive elements
evm.model.Contig139.120	ACE	GACACGTATG	849	light-responsive elements
evm.model.Contig139.120	MBS	CAACTG	1605	Site-binding related elements
evm.model.Contig139.120	MBS	CAACTG	1687	Site-binding related elements
evm.model.Contig139.120	MYB	CAACAG	1059	Site-binding related elements
evm.model.Contig139.120	CAAT-box	CAAT	141	promoter-related elements
evm.model.Contig139.120	CAAT-box	CAAT	225	promoter-related elements
evm.model.Contig139.120	CAAT-box	CAAT	237	promoter-related elements
evm.model.Contig139.120	CAAT-box	CAAAT	253	promoter-related elements
evm.model.Contig139.120	CAAT-box	CAAT	944	promoter-related elements
evm.model.Contig139.120	CAAT-box	CAAT	1091	promoter-related elements
evm.model.Contig139.120	CAAT-box	CAAT	1134	promoter-related elements
evm.model.Contig139.120	CAAT-box	CAAT	1154	promoter-related elements
evm.model.Contig139.120	CAAT-box	CCAAT	1296	promoter-related elements
evm.model.Contig139.120	CAAT-box	CAAAT	1337	promoter-related elements
evm.model.Contig139.120	CAAT-box	CAAT	1399	promoter-related elements
evm.model.Contig139.120	CAAT-box	CAAT	1437	promoter-related elements
evm.model.Contig139.120	CAAT-box	CAAT	1655	promoter-related elements
evm.model.Contig139.120	CAAT-box	CAAAT	1676	promoter-related elements
evm.model.Contig139.120	CAAT-box	CAAAT	1708	promoter-related elements
evm.model.Contig139.120	CAAT-box	CAAT	1736	promoter-related elements
evm.model.Contig139.120	CAAT-box	CAAAT	1790	promoter-related elements
evm.model.Contig139.120	CAAT-box	CCAAT	1802	promoter-related elements
evm.model.Contig139.120	CAAT-box	CAAT	1809	promoter-related elements
evm.model.Contig139.120	CAAT-box	CAAT	1859	promoter-related elements
evm.model.Contig139.120	CAAT-box	CAAT	1869	promoter-related elements
evm.model.Contig139.120	CAAT-box	CAAT	1953	promoter-related elements
evm.model.Contig139.120	CAAT-box	CAAT	1962	promoter-related elements

evm.model.Contig139.120	CAAT-box	CAAT	1972	promoter-related elements
evm.model.Contig139.120	CAAT-box	CCAAT	1988	promoter-related elements
evm.model.Contig139.120	CAAT-box	CAAT	1989	promoter-related elements
evm.model.Contig139.120	CAAT-box	CAAAT	1991	promoter-related elements
evm.model.Contig139.120	Box 4	ATTAAT	1253	light-responsive elements
evm.model.Contig139.120	Box 4	ATTAAT	1943	light-responsive elements
evm.model.Contig139.120	ERE	ATTTCATA	1665	homone-responsive elements
evm.model.Contig139.120	TGA-element	AACGAC	1265	homone-responsive elements
evm.model.Contig139.120	ARE	AAACCA	1691	environmental stress-related elements
evm.model.Contig139.120	RY-element	CATGCATG	817	other elements
evm.model.Contig139.120	AE-box	AGAAACAA	1099	light-responsive elements
evm.model.Contig139.120	G-Box	CACGTG	873	light-responsive elements
evm.model.Contig139.120	ABRE	ACGTG	454	homone-responsive elements
evm.model.Contig139.120	ABRE	ACGTG	852	homone-responsive elements
evm.model.Contig139.120	ABRE	CACGTG	873	homone-responsive elements
evm.model.Contig139.120	ABRE	ACGTG	874	homone-responsive elements
evm.model.Contig139.120	ABRE	ACGTG	1164	homone-responsive elements
evm.model.Contig139.217	MYB-like sequence	TAACCA	93	Site-binding related elements
evm.model.Contig139.217	MYB-like sequence	TAACCA	672	Site-binding related elements
evm.model.Contig139.217	MYB-like sequence	TAACCA	1922	Site-binding related elements
evm.model.Contig139.217	CAT-box	GCCACT	1468	other elements
evm.model.Contig139.217	LAMP-element	CTTTATCA	1164	light-responsive elements
evm.model.Contig139.217	circadian	CAAAGATATC	1023	other elements
evm.model.Contig139.217	TATA-box	TATACA	278	promoter-related elements
evm.model.Contig139.217	TATA-box	TATA	280	promoter-related elements
evm.model.Contig139.217	TATA-box	TATACA	296	promoter-related elements
evm.model.Contig139.217	TATA-box	TATA	298	promoter-related elements
evm.model.Contig139.217	TATA-box	TATATAA	419	promoter-related elements
evm.model.Contig139.217	TATA-box	TATATA	420	promoter-related elements
evm.model.Contig139.217	TATA-box	TATA	422	promoter-related elements
evm.model.Contig139.217	TATA-box	TACATAAA	552	promoter-related elements
evm.model.Contig139.217	TATA-box	ATATAT	784	promoter-related elements
evm.model.Contig139.217	TATA-box	TATA	785	promoter-related elements
evm.model.Contig139.217	TATA-box	TATA	867	promoter-related elements
evm.model.Contig139.217	TATA-box	ATATAT	875	promoter-related elements
evm.model.Contig139.217	TATA-box	TATA	876	promoter-related elements
evm.model.Contig139.217	TATA-box	ATATAT	1001	promoter-related elements
evm.model.Contig139.217	TATA-box	TATA	1002	promoter-related elements
evm.model.Contig139.217	TATA-box	TATA	1069	promoter-related elements
evm.model.Contig139.217	TATA-box	ATTATA	1307	promoter-related elements
evm.model.Contig139.217	TATA-box	TATATAA	1308	promoter-related elements
evm.model.Contig139.217	TATA-box	TATATA	1309	promoter-related elements
evm.model.Contig139.217	TATA-box	TATA	1311	promoter-related elements
evm.model.Contig139.217	TATA-box	ATATAT	1431	promoter-related elements
evm.model.Contig139.217	TATA-box	TATA	1432	promoter-related elements
evm.model.Contig139.217	TATA-box	ATATAT	1506	promoter-related elements
evm.model.Contig139.217	TATA-box	TATA	1507	promoter-related elements
evm.model.Contig139.217	TATA-box	TATA	1884	promoter-related elements
evm.model.Contig139.217	AACA_motif	TAACAAACTCCA	410	other elements
evm.model.Contig139.217	HD-Zip 3	GTAAT(G/C)ATTAC	426	Site-binding related elements
evm.model.Contig139.217	STRE	AGGGG	705	environmental stress-related elements
evm.model.Contig139.217	STRE	AGGGG	1405	environmental stress-related elements
evm.model.Contig139.217	STRE	AGGGG	1724	environmental stress-related elements
evm.model.Contig139.217	GT1-motif	GGTTAA	94	light-responsive elements
evm.model.Contig139.217	GT1-motif	GGTTAA	673	light-responsive elements
evm.model.Contig139.217	Myb	TAACTG	358	Site-binding related elements
evm.model.Contig139.217	Myb	TAACTG	975	Site-binding related elements
evm.model.Contig139.217	Myb	TAACTG	1454	Site-binding related elements
evm.model.Contig139.217	G-box	CACGTG	149	light-responsive elements
evm.model.Contig139.217	CGTCA-motif	CGTCA	736	homone-responsive elements
evm.model.Contig139.217	CGTCA-motif	CGTCA	826	homone-responsive elements
evm.model.Contig139.217	CGTCA-motif	CGTCA	1656	homone-responsive elements
evm.model.Contig139.217	CGTCA-motif	CGTCA	1750	homone-responsive elements
evm.model.Contig139.217	AE-box	AGAAACAA	1228	light-responsive elements
evm.model.Contig139.217	G-Box	CACGTG	149	light-responsive elements
evm.model.Contig139.217	TC-rich repeats	GTTTTCTTAC	571	environmental stress-related elements
evm.model.Contig139.217	Unnamed__1	GAATTTAATTAA	1201	site-binding related elements
evm.model.Contig139.217	ABRE	CACGTG	149	homone-responsive elements
evm.model.Contig139.217	ABRE	ACGTG	150	homone-responsive elements
evm.model.Contig139.217	O2-site	GATGATGTGG	205	other elements
evm.model.Contig139.217	Box 4	ATTAAT	562	light-responsive elements
evm.model.Contig139.217	Box 4	ATTAAT	1020	light-responsive elements
evm.model.Contig139.217	Box 4	ATTAAT	1241	light-responsive elements
evm.model.Contig139.217	Box 4	ATTAAT	1531	light-responsive elements
evm.model.Contig139.217	CAAT-box	CAAT	264	promoter-related elements
evm.model.Contig139.217	CAAT-box	CAAT	266	promoter-related elements
evm.model.Contig139.217	CAAT-box	CAAAT	414	promoter-related elements
evm.model.Contig139.217	CAAT-box	CAAT	491	promoter-related elements

evm.model.Contig139.217	CAAT-box	CAAT	566	promoter-related elements
evm.model.Contig139.217	CAAT-box	CAAT	595	promoter-related elements
evm.model.Contig139.217	CAAT-box	CAAT	605	promoter-related elements
evm.model.Contig139.217	CAAT-box	TGCCAAC	628	promoter-related elements
evm.model.Contig139.217	CAAT-box	CAAT	636	promoter-related elements
evm.model.Contig139.217	CAAT-box	CCAAT	659	promoter-related elements
evm.model.Contig139.217	CAAT-box	CAAT	660	promoter-related elements
evm.model.Contig139.217	CAAT-box	CAAAT	717	promoter-related elements
evm.model.Contig139.217	CAAT-box	CAAAT	760	promoter-related elements
evm.model.Contig139.217	CAAT-box	CAAT	775	promoter-related elements
evm.model.Contig139.217	CAAT-box	CAAT	805	promoter-related elements
evm.model.Contig139.217	CAAT-box	CAAT	809	promoter-related elements
evm.model.Contig139.217	CAAT-box	CAAT	850	promoter-related elements
evm.model.Contig139.217	CAAT-box	CAAT	910	promoter-related elements
evm.model.Contig139.217	CAAT-box	CAAT	914	promoter-related elements
evm.model.Contig139.217	CAAT-box	CAAT	944	promoter-related elements
evm.model.Contig139.217	CAAT-box	CAAT	963	promoter-related elements
evm.model.Contig139.217	CAAT-box	CAAAT	965	promoter-related elements
evm.model.Contig139.217	CAAT-box	CAAT	982	promoter-related elements
evm.model.Contig139.217	CAAT-box	CAAAT	995	promoter-related elements
evm.model.Contig139.217	CAAT-box	CCAAT	1121	promoter-related elements
evm.model.Contig139.217	CAAT-box	CAAT	1149	promoter-related elements
evm.model.Contig139.217	CAAT-box	CAAT	1233	promoter-related elements
evm.model.Contig139.217	CAAT-box	CAAAT	1275	promoter-related elements
evm.model.Contig139.217	CAAT-box	CAAAT	1355	promoter-related elements
evm.model.Contig139.217	CAAT-box	CAAAT	1413	promoter-related elements
evm.model.Contig139.217	CAAT-box	CAAT	1510	promoter-related elements
evm.model.Contig139.217	CAAT-box	CAAAT	1517	promoter-related elements
evm.model.Contig139.217	CAAT-box	CAAT	1659	promoter-related elements
evm.model.Contig139.217	CAAT-box	CAAT	1790	promoter-related elements
evm.model.Contig139.217	CAAT-box	CAAT	1876	promoter-related elements
evm.model.Contig139.217	CAAT-box	CAAT	1942	promoter-related elements
evm.model.Contig139.217	CAAT-box	CAAT	1964	promoter-related elements
evm.model.Contig139.217	MYB	CAACCA	3	Site-binding related elements
evm.model.Contig139.217	MYB	TAACCA	93	Site-binding related elements
evm.model.Contig139.217	MYB	CAACCA	626	Site-binding related elements
evm.model.Contig139.217	MYB	TAACCA	672	Site-binding related elements
evm.model.Contig139.217	MYB	TAACCA	1922	Site-binding related elements
evm.model.Contig139.217	TGACG-motif	TGACG	736	homone-responsive elements
evm.model.Contig139.217	TGACG-motif	TGACG	826	homone-responsive elements
evm.model.Contig139.217	TGACG-motif	TGACG	1656	homone-responsive elements
evm.model.Contig139.217	TGACG-motif	TGACG	1750	homone-responsive elements
evm.model.Contig139.217	MYC	CATGTG	29	Site-binding related elements
evm.model.Contig139.217	MYC	CAATTG	264	Site-binding related elements
evm.model.Contig139.217	MYC	CATGTG	1840	Site-binding related elements
evm.model.Contig139.217	ARE	AAACCA	1332	environmental stress-related elements
evm.model.Contig139.217	ERE	ATTTTAAA	1327	homone-responsive elements
evm.model.Contig139.217	P-box	CCTTTTG	182	homone-responsive elements
evm.model.Contig139.217	WRE3	CCACCT	893	environmental stress-related elements
evm.model.Contig154.585	TATA-box	ATTATA	355	promoter-related elements
evm.model.Contig154.585	TATA-box	TATAA	356	promoter-related elements
evm.model.Contig154.585	TATA-box	TATA	357	promoter-related elements
evm.model.Contig154.585	TATA-box	TATA	754	promoter-related elements
evm.model.Contig154.585	CCGTCC-box	CCGTCC	601	other elements
evm.model.Contig154.585	CAT-box	GCCACT	168	other elements
evm.model.Contig154.585	GT1-motif	GTGTGTGAA	59	light-responsive elements
evm.model.Contig154.585	GT1-motif	GGTTAA	248	light-responsive elements
evm.model.Contig154.585	GT1-motif	GGTTAA	873	light-responsive elements
evm.model.Contig154.585	STRE	AGGGG	916	environmental stress-related elements
evm.model.Contig154.585	STRE	AGGGG	1042	environmental stress-related elements
evm.model.Contig154.585	STRE	AGGGG	1577	environmental stress-related elements
evm.model.Contig154.585	MRE	AACCTAA	1674	Site-binding related elements
evm.model.Contig154.585	MRE	AACCTAA	1742	Site-binding related elements
evm.model.Contig154.585	CGTCA-motif	CGTCA	1888	homone-responsive elements
evm.model.Contig154.585	G-box	ACACGTGT	23	light-responsive elements
evm.model.Contig154.585	G-box	CACGTG	24	light-responsive elements
evm.model.Contig154.585	G-box	CACGTG	85	light-responsive elements
evm.model.Contig154.585	G-box	ACACGTGT	615	light-responsive elements
evm.model.Contig154.585	G-box	CACGTG	616	light-responsive elements
evm.model.Contig154.585	G-box	CACGTG	952	light-responsive elements
evm.model.Contig154.585	G-box	CACGTC	1508	light-responsive elements
evm.model.Contig154.585	G-box	CACGAC	1511	light-responsive elements
evm.model.Contig154.585	G-Box	CACGTG	24	light-responsive elements
evm.model.Contig154.585	G-Box	CACGTG	85	light-responsive elements
evm.model.Contig154.585	G-Box	CACGTG	616	light-responsive elements
evm.model.Contig154.585	G-Box	CACGTG	952	light-responsive elements
evm.model.Contig154.585	A-box	CCGTCC	601	other elements
evm.model.Contig154.585	4cl-CMA2b	TCTCACCAACCCCA	1340	light-responsive elements

evm.model.Contig154.585	ABRE	CGCACGTGTC	22	homone-responsive elements
evm.model.Contig154.585	ABRE	CACGTG	24	homone-responsive elements
evm.model.Contig154.585	ABRE	ACGTG	25	homone-responsive elements
evm.model.Contig154.585	ABRE	CACGTG	85	homone-responsive elements
evm.model.Contig154.585	ABRE	ACGTG	86	homone-responsive elements
evm.model.Contig154.585	ABRE	CGCACGTGTC	614	homone-responsive elements
evm.model.Contig154.585	ABRE	CACGTG	616	homone-responsive elements
evm.model.Contig154.585	ABRE	ACGTG	617	homone-responsive elements
evm.model.Contig154.585	ABRE	CGCACGTGTC	950	homone-responsive elements
evm.model.Contig154.585	ABRE	CACGTG	952	homone-responsive elements
evm.model.Contig154.585	ABRE	ACGTG	953	homone-responsive elements
evm.model.Contig154.585	ABRE	ACGTG	1508	homone-responsive elements
evm.model.Contig154.585	TC-rich repeats	ATTCTCTAAC	1483	environmental stress-related elements
evm.model.Contig154.585	TC-rich repeats	ATTCTCTAAC	1775	environmental stress-related elements
evm.model.Contig154.585	TGACG-motif	TGACG	1888	homone-responsive elements
evm.model.Contig154.585	MYC	CATTTG	460	Site-binding related elements
evm.model.Contig154.585	ACE	GACACGTATG	22	light-responsive elements
evm.model.Contig154.585	ACE	GACACGTATG	558	light-responsive elements
evm.model.Contig154.585	ACE	GACACGTATG	614	light-responsive elements
evm.model.Contig154.585	Box 4	ATTAAT	366	light-responsive elements
evm.model.Contig154.585	Box 4	ATTAAT	1180	light-responsive elements
evm.model.Contig154.585	Box 4	ATTAAT	1637	light-responsive elements
evm.model.Contig154.585	CAAT-box	CCAAT	13	promoter-related elements
evm.model.Contig154.585	CAAT-box	CAAT	14	promoter-related elements
evm.model.Contig154.585	CAAT-box	CAAAT	55	promoter-related elements
evm.model.Contig154.585	CAAT-box	CAAAT	116	promoter-related elements
evm.model.Contig154.585	CAAT-box	CAAAT	176	promoter-related elements
evm.model.Contig154.585	CAAT-box	CAAAT	208	promoter-related elements
evm.model.Contig154.585	CAAT-box	CAAAT	259	promoter-related elements
evm.model.Contig154.585	CAAT-box	CAAAT	286	promoter-related elements
evm.model.Contig154.585	CAAT-box	CAAT	289	promoter-related elements
evm.model.Contig154.585	CAAT-box	CAAAT	305	promoter-related elements
evm.model.Contig154.585	CAAT-box	CAAAT	322	promoter-related elements
evm.model.Contig154.585	CAAT-box	CAAT	325	promoter-related elements
evm.model.Contig154.585	CAAT-box	CAAAT	460	promoter-related elements
evm.model.Contig154.585	CAAT-box	CCAAT	605	promoter-related elements
evm.model.Contig154.585	CAAT-box	CAAT	606	promoter-related elements
evm.model.Contig154.585	CAAT-box	CAAT	711	promoter-related elements
evm.model.Contig154.585	CAAT-box	CAAT	778	promoter-related elements
evm.model.Contig154.585	CAAT-box	CAAT	782	promoter-related elements
evm.model.Contig154.585	CAAT-box	CAAT	794	promoter-related elements
evm.model.Contig154.585	CAAT-box	CAAT	830	promoter-related elements
evm.model.Contig154.585	CAAT-box	CAAT	1054	promoter-related elements
evm.model.Contig154.585	CAAT-box	CAAT	1209	promoter-related elements
evm.model.Contig154.585	CAAT-box	CAAT	1497	promoter-related elements
evm.model.Contig154.585	CAAT-box	CAAAT	1592	promoter-related elements
evm.model.Contig154.585	CAAT-box	CAAT	1666	promoter-related elements
evm.model.Contig154.585	CAAT-box	CAAT	1671	promoter-related elements
evm.model.Contig154.585	CAAT-box	CAAAT	1701	promoter-related elements
evm.model.Contig154.585	CAAT-box	CAAT	1765	promoter-related elements
evm.model.Contig154.585	CAAT-box	CAAT	1771	promoter-related elements
evm.model.Contig154.585	CAAT-box	CAAT	1809	promoter-related elements
evm.model.Contig154.585	CAAT-box	CAAT	1838	promoter-related elements
evm.model.Contig154.585	CAAT-box	CAAAT	1862	promoter-related elements
evm.model.Contig154.585	CAAT-box	CAAT	1952	promoter-related elements
evm.model.Contig154.585	CAAT-box	CAAAT	1980	promoter-related elements
evm.model.Contig154.585	LTR	CCGAAA	1618	environmental stress-related elements
evm.model.Contig154.585	ARE	AAACCA	35	environmental stress-related elements
evm.model.Contig154.585	ARE	AAACCA	1298	environmental stress-related elements
evm.model.Contig154.585	ERE	ATTTCATA	1446	homone-responsive elements
evm.model.Contig21.35	ERE	ATTTTAAA	1476	homone-responsive elements
evm.model.Contig21.35	TGA-element	AACGAC	757	homone-responsive elements
evm.model.Contig21.35	ARE	AAACCA	515	environmental stress-related elements
evm.model.Contig21.35	ARE	AAACCA	545	environmental stress-related elements
evm.model.Contig21.35	ARE	AAACCA	1761	environmental stress-related elements
evm.model.Contig21.35	ARE	AAACCA	1874	environmental stress-related elements
evm.model.Contig21.35	WRE3	CCACCT	1881	environmental stress-related elements
evm.model.Contig21.35	WRE3	CCACCT	1888	environmental stress-related elements
evm.model.Contig21.35	WRE3	CCACCT	1895	environmental stress-related elements
evm.model.Contig21.35	WUN-motif	AAATTACT	1719	environmental stress-related elements
evm.model.Contig21.35	MYC	CATGTG	511	Site-binding related elements
evm.model.Contig21.35	MYC	CATTTG	612	Site-binding related elements
evm.model.Contig21.35	MYC	CAATTG	1122	Site-binding related elements
evm.model.Contig21.35	MYC	CATTTG	1683	Site-binding related elements
evm.model.Contig21.35	MYC	CAATTG	1811	Site-binding related elements
evm.model.Contig21.35	TGACG-motif	TGACG	99	homone-responsive elements
evm.model.Contig21.35	TGACG-motif	TGACG	768	homone-responsive elements
evm.model.Contig21.35	MYB	CAACCA	195	Site-binding related elements

evm.model.Contig21.35	MYB	CAACCA	1878	Site-binding related elements
evm.model.Contig21.35	CAAT-box	CAAT	54	promoter-related elements
evm.model.Contig21.35	CAAT-box	CAAT	63	promoter-related elements
evm.model.Contig21.35	CAAT-box	CAAT	77	promoter-related elements
evm.model.Contig21.35	CAAT-box	CAAT	105	promoter-related elements
evm.model.Contig21.35	CAAT-box	CAAT	147	promoter-related elements
evm.model.Contig21.35	CAAT-box	CAAAT	264	promoter-related elements
evm.model.Contig21.35	CAAT-box	CAAT	279	promoter-related elements
evm.model.Contig21.35	CAAT-box	CAAT	330	promoter-related elements
evm.model.Contig21.35	CAAT-box	CAAT	354	promoter-related elements
evm.model.Contig21.35	CAAT-box	CAAT	418	promoter-related elements
evm.model.Contig21.35	CAAT-box	CAAT	444	promoter-related elements
evm.model.Contig21.35	CAAT-box	CAAAT	524	promoter-related elements
evm.model.Contig21.35	CAAT-box	CAAAT	554	promoter-related elements
evm.model.Contig21.35	CAAT-box	CAAAT	612	promoter-related elements
evm.model.Contig21.35	CAAT-box	CAAAT	644	promoter-related elements
evm.model.Contig21.35	CAAT-box	CAAAT	677	promoter-related elements
evm.model.Contig21.35	CAAT-box	CCAAT	708	promoter-related elements
evm.model.Contig21.35	CAAT-box	CAAT	709	promoter-related elements
evm.model.Contig21.35	CAAT-box	CAAAT	747	promoter-related elements
evm.model.Contig21.35	CAAT-box	CAAT	819	promoter-related elements
evm.model.Contig21.35	CAAT-box	CAAT	835	promoter-related elements
evm.model.Contig21.35	CAAT-box	CAAT	863	promoter-related elements
evm.model.Contig21.35	CAAT-box	CAAT	870	promoter-related elements
evm.model.Contig21.35	CAAT-box	CAAT	911	promoter-related elements
evm.model.Contig21.35	CAAT-box	CCAAT	947	promoter-related elements
evm.model.Contig21.35	CAAT-box	CAAT	952	promoter-related elements
evm.model.Contig21.35	CAAT-box	CAAAT	976	promoter-related elements
evm.model.Contig21.35	CAAT-box	CAAAT	1023	promoter-related elements
evm.model.Contig21.35	CAAT-box	CAAT	1043	promoter-related elements
evm.model.Contig21.35	CAAT-box	CCAAT	1114	promoter-related elements
evm.model.Contig21.35	CAAT-box	CAAT	1115	promoter-related elements
evm.model.Contig21.35	CAAT-box	CAAT	1122	promoter-related elements
evm.model.Contig21.35	CAAT-box	CAAT	1124	promoter-related elements
evm.model.Contig21.35	CAAT-box	CAAT	1223	promoter-related elements
evm.model.Contig21.35	CAAT-box	CAAT	1271	promoter-related elements
evm.model.Contig21.35	CAAT-box	CAAT	1328	promoter-related elements
evm.model.Contig21.35	CAAT-box	CCAAT	1407	promoter-related elements
evm.model.Contig21.35	CAAT-box	CAAAT	1416	promoter-related elements
evm.model.Contig21.35	CAAT-box	CAAAT	1446	promoter-related elements
evm.model.Contig21.35	CAAT-box	CAAAT	1552	promoter-related elements
evm.model.Contig21.35	CAAT-box	CAAAT	1565	promoter-related elements
evm.model.Contig21.35	CAAT-box	CAAAT	1595	promoter-related elements
evm.model.Contig21.35	CAAT-box	CAAAT	1601	promoter-related elements
evm.model.Contig21.35	CAAT-box	CAAAT	1654	promoter-related elements
evm.model.Contig21.35	CAAT-box	CAAAT	1684	promoter-related elements
evm.model.Contig21.35	CAAT-box	CAAAT	1723	promoter-related elements
evm.model.Contig21.35	CAAT-box	CAAAT	1730	promoter-related elements
evm.model.Contig21.35	CAAT-box	CCAAT	1745	promoter-related elements
evm.model.Contig21.35	CAAT-box	CAAAT	1753	promoter-related elements
evm.model.Contig21.35	CAAT-box	CAAT	1811	promoter-related elements
evm.model.Contig21.35	CAAT-box	CCAAT	1813	promoter-related elements
evm.model.Contig21.35	CAAT-box	CAAT	1831	promoter-related elements
evm.model.Contig21.35	CAAT-box	CAAT	1933	promoter-related elements
evm.model.Contig21.35	Box 4	ATTAAT	217	light-responsive elements
evm.model.Contig21.35	Box 4	ATTAAT	318	light-responsive elements
evm.model.Contig21.35	Box 4	ATTAAT	716	light-responsive elements
evm.model.Contig21.35	Box 4	ATTAAT	829	light-responsive elements
evm.model.Contig21.35	Box 4	ATTAAT	1291	light-responsive elements
evm.model.Contig21.35	Box 4	ATTAAT	1783	light-responsive elements
evm.model.Contig21.35	ATCT-motif	AATCTAATCC	110	light-responsive elements
evm.model.Contig21.35	TCT-motif	TCTTAC	858	light-responsive elements
evm.model.Contig21.35	TCT-motif	TCTTAC	1257	light-responsive elements
evm.model.Contig21.35	ABRE	CACGTG	1805	homone-responsive elements
evm.model.Contig21.35	ABRE	ACGTG	1806	homone-responsive elements
evm.model.Contig21.35	G-Box	CACGTG	1805	light-responsive elements
evm.model.Contig21.35	CGTCA-motif	CGTCA	99	homone-responsive elements
evm.model.Contig21.35	CGTCA-motif	CGTCA	768	homone-responsive elements
evm.model.Contig21.35	G-box	CACGAC	1193	light-responsive elements
evm.model.Contig21.35	G-box	CACGTG	1805	light-responsive elements
evm.model.Contig21.35	STRE	AGGGG	488	environmental stress-related elements
evm.model.Contig21.35	STRE	AGGGG	600	environmental stress-related elements
evm.model.Contig21.35	STRE	AGGGG	762	environmental stress-related elements
evm.model.Contig21.35	STRE	AGGGG	1646	environmental stress-related elements
evm.model.Contig21.35	STRE	AGGGG	1677	environmental stress-related elements
evm.model.Contig21.35	AT-rich element	ATAGAAATCAA	1925	site-binding related elements
evm.model.Contig21.35	GT1-motif	GGTTAAT	491	light-responsive elements
evm.model.Contig21.35	GT1-motif	GGTTAA	565	light-responsive elements

evm.model.Contig21.35	GT1-motif	GGTTAA	1534	light-responsive elements
evm.model.Contig21.35	TCCC-motif	TCTCCCT	1980	light-responsive elements
evm.model.Contig21.35	GATA-motif	GATAGGG	1643	light-responsive elements
evm.model.Contig21.35	CAT-box	GCCACT	965	other elements
evm.model.Contig21.35	W box	TTGACC	521	site-binding related elements
evm.model.Contig21.35	W box	TTGACC	549	site-binding related elements
evm.model.Contig21.35	W box	TTGACC	1133	site-binding related elements
evm.model.Contig21.35	W box	TTGACC	1569	site-binding related elements
evm.model.Contig21.35	W box	TTGACC	1727	site-binding related elements
evm.model.Contig21.35	TATA-box	ATTATA	117	promoter-related elements
evm.model.Contig21.35	TATA-box	TATAA	118	promoter-related elements
evm.model.Contig21.35	TATA-box	TATA	119	promoter-related elements
evm.model.Contig21.35	TATA-box	taTATAAAtc	231	promoter-related elements
evm.model.Contig21.35	TATA-box	TATAAAT	232	promoter-related elements
evm.model.Contig21.35	TATA-box	TATAAA	233	promoter-related elements
evm.model.Contig21.35	TATA-box	TATAA	234	promoter-related elements
evm.model.Contig21.35	TATA-box	TATA	235	promoter-related elements
evm.model.Contig21.35	TATA-box	TATAA	287	promoter-related elements
evm.model.Contig21.35	TATA-box	TATA	288	promoter-related elements
evm.model.Contig21.35	TATA-box	TACAAAA	370	promoter-related elements
evm.model.Contig21.35	TATA-box	TATAA	640	promoter-related elements
evm.model.Contig21.35	TATA-box	TATA	641	promoter-related elements
evm.model.Contig21.35	TATA-box	taTATAAAtc	715	promoter-related elements
evm.model.Contig21.35	TATA-box	ATATAT	720	promoter-related elements
evm.model.Contig21.35	TATA-box	TATA	721	promoter-related elements
evm.model.Contig21.35	TATA-box	TACAAAA	752	promoter-related elements
evm.model.Contig21.35	TATA-box	TATATAA	893	promoter-related elements
evm.model.Contig21.35	TATA-box	TATATA	894	promoter-related elements
evm.model.Contig21.35	TATA-box	TATA	896	promoter-related elements
evm.model.Contig21.35	TATA-box	TATATAA	935	promoter-related elements
evm.model.Contig21.35	TATA-box	TATATA	936	promoter-related elements
evm.model.Contig21.35	TATA-box	TATA	938	promoter-related elements
evm.model.Contig21.35	TATA-box	TATAAAA	1231	promoter-related elements
evm.model.Contig21.35	TATA-box	TATAAA	1232	promoter-related elements
evm.model.Contig21.35	TATA-box	TATAA	1233	promoter-related elements
evm.model.Contig21.35	TATA-box	TATA	1234	promoter-related elements
evm.model.Contig21.35	TATA-box	taTATAAAtc	1236	promoter-related elements
evm.model.Contig21.35	TATA-box	TATA	1265	promoter-related elements
evm.model.Contig21.35	TATA-box	ATTATA	1366	promoter-related elements
evm.model.Contig21.35	TATA-box	TATAA	1367	promoter-related elements
evm.model.Contig21.35	TATA-box	TATA	1368	promoter-related elements
evm.model.Contig21.35	TATA-box	ATATAA	1389	promoter-related elements
evm.model.Contig21.35	TATA-box	TATA	1390	promoter-related elements
evm.model.Contig21.35	TATA-box	ccTATAAAaa	1397	promoter-related elements
evm.model.Contig21.35	TATA-box	TATAAAA	1398	promoter-related elements
evm.model.Contig21.35	TATA-box	TATAAA	1399	promoter-related elements
evm.model.Contig21.35	TATA-box	TATAA	1400	promoter-related elements
evm.model.Contig21.35	TATA-box	TATA	1401	promoter-related elements
evm.model.Contig21.35	TATA-box	TATAAAA	1439	promoter-related elements
evm.model.Contig21.35	TATA-box	TATAAA	1440	promoter-related elements
evm.model.Contig21.35	TATA-box	TATAA	1441	promoter-related elements
evm.model.Contig21.35	TATA-box	TATA	1442	promoter-related elements
evm.model.Contig21.35	TATA-box	ATATAA	1560	promoter-related elements
evm.model.Contig21.35	TATA-box	TATA	1561	promoter-related elements
evm.model.Contig21.35	TATA-box	TATA	1916	promoter-related elements
evm.model.Contig267.36	ERE	ATTTCATA	13	homone-responsive elements
evm.model.Contig267.36	WRE3	CCACCT	37	environmental stress-related elements
evm.model.Contig267.36	MYB	CAACCA	56	Site-binding related elements
evm.model.Contig267.36	CAAT-box	CCAAT	59	promoter-related elements
evm.model.Contig267.36	CAAT-box	CAAT	60	promoter-related elements
evm.model.Contig267.36	STRE	AGGGG	112	environmental stress-related elements
evm.model.Contig267.36	W box	TTGACC	116	site-binding related elements
evm.model.Contig267.36	CGTCA-motif	CGTCA	199	homone-responsive elements
evm.model.Contig267.36	TGACG-motif	TGACG	199	homone-responsive elements
evm.model.Contig267.36	CAAT-box	CAAT	250	promoter-related elements
evm.model.Contig267.36	CAAT-box	CAAT	257	promoter-related elements
evm.model.Contig267.36	CAAT-box	CAAT	264	promoter-related elements
evm.model.Contig267.36	CAAT-box	CAAT	299	promoter-related elements
evm.model.Contig267.36	CAAT-box	CAAT	303	promoter-related elements
evm.model.Contig267.36	TATA-box	TATACA	337	promoter-related elements
evm.model.Contig267.36	TATA-box	TATA	339	promoter-related elements
evm.model.Contig267.36	GCN4_motif	TGAGTCA	376	other elements
evm.model.Contig267.36	MYC	CATGTG	381	Site-binding related elements
evm.model.Contig267.36	TATA-box	ATTATA	391	promoter-related elements
evm.model.Contig267.36	TATA-box	TATAA	392	promoter-related elements
evm.model.Contig267.36	TATA-box	TATA	393	promoter-related elements
evm.model.Contig267.36	Myb	TAACTG	395	Site-binding related elements
evm.model.Contig267.36	CAAT-box	CAAAT	424	promoter-related elements

evm.model.Contig267.36	MYC	CATTTG	424	Site-binding related elements
evm.model.Contig267.36	CAAT-box	CAAT	459	promoter-related elements
evm.model.Contig267.36	CAAT-box	CAAT	468	promoter-related elements
evm.model.Contig267.36	MYC	CAATTG	468	Site-binding related elements
evm.model.Contig267.36	CAAT-box	CCAAT	470	promoter-related elements
evm.model.Contig267.36	MYC	CATGTG	483	Site-binding related elements
evm.model.Contig267.36	Myb	CAACTG	500	Site-binding related elements
evm.model.Contig267.36	MBS	CAACTG	500	Site-binding related elements
evm.model.Contig267.36	CAAT-box	TGCCAAC	502	promoter-related elements
evm.model.Contig267.36	TATA-box	TATA	522	promoter-related elements
evm.model.Contig267.36	TCT-motif	TCTTAC	531	light-responsive elements
evm.model.Contig267.36	TATA-box	TATA	567	promoter-related elements
evm.model.Contig267.36	TATA-box	TATA	572	promoter-related elements
evm.model.Contig267.36	CAAT-box	CAAAT	598	promoter-related elements
evm.model.Contig267.36	CAAT-box	CAAAT	619	promoter-related elements
evm.model.Contig267.36	TATA-box	ATTATA	622	promoter-related elements
evm.model.Contig267.36	TATA-box	TATAA	623	promoter-related elements
evm.model.Contig267.36	TATA-box	TATA	624	promoter-related elements
evm.model.Contig267.36	Unnamed__1	GAATTTAATTAA	646	site-binding related elements
evm.model.Contig267.36	TATA-box	ATATAA	648	promoter-related elements
evm.model.Contig267.36	TATA-box	TATA	649	promoter-related elements
evm.model.Contig267.36	Box 4	ATTAAT	719	light-responsive elements
evm.model.Contig267.36	TATA-box	TATA	724	promoter-related elements
evm.model.Contig267.36	MYB-like sequence	TAACCA	748	Site-binding related elements
evm.model.Contig267.36	MYB	TAACCA	748	Site-binding related elements
evm.model.Contig267.36	MYC	CATTTG	781	Site-binding related elements
evm.model.Contig267.36	CAAT-box	CAAAT	782	promoter-related elements
evm.model.Contig267.36	TATA-box	ATATAA	845	promoter-related elements
evm.model.Contig267.36	TATA-box	TATA	846	promoter-related elements
evm.model.Contig267.36	AT-rich sequence	TAAAATACT	852	other elements
evm.model.Contig267.36	TATA-box	ATTATA	866	promoter-related elements
evm.model.Contig267.36	TATA-box	TATAA	867	promoter-related elements
evm.model.Contig267.36	TATA-box	TATA	868	promoter-related elements
evm.model.Contig267.36	CAAT-box	CAAT	889	promoter-related elements
evm.model.Contig267.36	CCGTCC-box	CCGTCC	947	other elements
evm.model.Contig267.36	A-box	CCGTCC	947	other elements
evm.model.Contig267.36	CAAT-box	CAAT	967	promoter-related elements
evm.model.Contig267.36	TATA-box	TACATAAA	1020	promoter-related elements
evm.model.Contig267.36	TATA-box	TATACA	1024	promoter-related elements
evm.model.Contig267.36	TATA-box	TATA	1026	promoter-related elements
evm.model.Contig267.36	CGTCA-motif	CGTCA	1074	homone-responsive elements
evm.model.Contig267.36	TGACG-motif	TGACG	1074	homone-responsive elements
evm.model.Contig267.36	CAAT-box	CAAT	1077	promoter-related elements
evm.model.Contig267.36	CAAT-box	CCAAT	1086	promoter-related elements
evm.model.Contig267.36	CAAT-box	CAAT	1087	promoter-related elements
evm.model.Contig267.36	MRE	AACCTAA	1112	Site-binding related elements
evm.model.Contig267.36	MYC	CATTTG	1163	Site-binding related elements
evm.model.Contig267.36	CAAT-box	CAAAT	1164	promoter-related elements
evm.model.Contig267.36	CAAT-box	CAAT	1169	promoter-related elements
evm.model.Contig267.36	LAMP-element	CTTTATCA	1189	light-responsive elements
evm.model.Contig267.36	CAAT-box	CAAAT	1199	promoter-related elements
evm.model.Contig267.36	TATA-box	TATA	1224	promoter-related elements
evm.model.Contig267.36	MYB	CAACCA	1236	Site-binding related elements
evm.model.Contig267.36	ARE	AAACCA	1243	environmental stress-related elements
evm.model.Contig267.36	CAAT-box	CCAAT	1290	promoter-related elements
evm.model.Contig267.36	CAAT-box	CAAT	1291	promoter-related elements
evm.model.Contig267.36	CAAT-box	CAAT	1297	promoter-related elements
evm.model.Contig267.36	CAAT-box	CAAAT	1299	promoter-related elements
evm.model.Contig267.36	CAAT-box	TGCCAAC	1303	promoter-related elements
evm.model.Contig267.36	STRE	AGGGG	1347	environmental stress-related elements
evm.model.Contig267.36	CAAT-box	CAAT	1363	promoter-related elements
evm.model.Contig267.36	Myb	TAACTG	1384	Site-binding related elements
evm.model.Contig267.36	CAAT-box	CAAT	1412	promoter-related elements
evm.model.Contig267.36	CAAT-box	CAAT	1438	promoter-related elements
evm.model.Contig267.36	CAAT-box	CAAAT	1440	promoter-related elements
evm.model.Contig267.36	CAAT-box	CAAT	1453	promoter-related elements
evm.model.Contig267.36	CAAT-box	CAAAT	1458	promoter-related elements
evm.model.Contig267.36	CAAT-box	CCAAT	1538	promoter-related elements
evm.model.Contig267.36	CAAT-box	CCAAT	1545	promoter-related elements
evm.model.Contig267.36	MYB	CAACCA	1547	Site-binding related elements
evm.model.Contig267.36	CAAT-box	CAAAT	1561	promoter-related elements
evm.model.Contig267.36	WRE3	CCACCT	1574	environmental stress-related elements
evm.model.Contig267.36	ARE	AAACCA	1605	environmental stress-related elements
evm.model.Contig267.36	TATA-box	TATAAATA	1637	promoter-related elements
evm.model.Contig267.36	TATA-box	TATAAAT	1638	promoter-related elements
evm.model.Contig267.36	TATA-box	TATAAA	1639	promoter-related elements
evm.model.Contig267.36	TATA-box	TATAA	1640	promoter-related elements
evm.model.Contig267.36	TATA-box	TATA	1641	promoter-related elements

evm.model.Contig267.36	CAAT-box	CAAT	1682	promoter-related elements
evm.model.Contig267.36	CAAT-box	CAAT	1699	promoter-related elements
evm.model.Contig267.36	TATA-box	ATATAA	1717	promoter-related elements
evm.model.Contig267.36	TATA-box	TATA	1718	promoter-related elements
evm.model.Contig267.36	CAAT-box	CAAT	1722	promoter-related elements
evm.model.Contig267.36	CAAT-box	CAAT	1730	promoter-related elements
evm.model.Contig267.36	G-box	TACGTG	1734	light-responsive elements
evm.model.Contig267.36	ABRE	ACGTG	1735	homone-responsive elements
evm.model.Contig267.36	MYC	CATTTG	1758	Site-binding related elements
evm.model.Contig267.36	CAAT-box	CAAAT	1759	promoter-related elements
evm.model.Contig267.36	MYC	CATGTG	1784	Site-binding related elements
evm.model.Contig267.36	CAAT-box	CAAT	1839	promoter-related elements
evm.model.Contig267.36	CAAT-box	CAAT	1856	promoter-related elements
evm.model.Contig267.36	TATA-box	TATA	1862	promoter-related elements
evm.model.Contig267.36	Myb	CAACTG	1870	Site-binding related elements
evm.model.Contig267.36	MBS	CAACTG	1870	Site-binding related elements
evm.model.Contig267.36	ERE	ATTTCATA	1905	homone-responsive elements
evm.model.Contig267.36	TATA-box	ATATAA	1928	promoter-related elements
evm.model.Contig267.36	TATA-box	TATA	1929	promoter-related elements
evm.model.Contig267.36	LTR	CCGAAA	1965	environmental stress-related elements
evm.model.Contig267.36	TCT-motif	TCTTAC	1988	light-responsive elements
evm.model.Contig267.36	TCCC-motif	TCTCCCT	1993	light-responsive elements
evm.model.Contig371.14	GCN4_motif	TGAGTCA	1635	other elements
evm.model.Contig371.14	G-Box	CACGTG	1532	light-responsive elements
evm.model.Contig371.14	ABRE	CACGTG	1532	homone-responsive elements
evm.model.Contig371.14	ABRE	ACGTG	1533	homone-responsive elements
evm.model.Contig371.14	Box 4	ATTAAT	288	light-responsive elements
evm.model.Contig371.14	Box 4	ATTAAT	1351	light-responsive elements
evm.model.Contig371.14	Box 4	ATTAAT	1871	light-responsive elements
evm.model.Contig371.14	CAAT-box	CAAAT	54	promoter-related elements
evm.model.Contig371.14	CAAT-box	CAAT	137	promoter-related elements
evm.model.Contig371.14	CAAT-box	CAAT	259	promoter-related elements
evm.model.Contig371.14	CAAT-box	TGCCAAC	262	promoter-related elements
evm.model.Contig371.14	CAAT-box	CAAT	281	promoter-related elements
evm.model.Contig371.14	CAAT-box	CAAAT	475	promoter-related elements
evm.model.Contig371.14	CAAT-box	CAAAT	487	promoter-related elements
evm.model.Contig371.14	CAAT-box	CAAT	520	promoter-related elements
evm.model.Contig371.14	CAAT-box	CAAT	527	promoter-related elements
evm.model.Contig371.14	CAAT-box	CAAAT	544	promoter-related elements
evm.model.Contig371.14	CAAT-box	CAAT	592	promoter-related elements
evm.model.Contig371.14	CAAT-box	CAAAT	609	promoter-related elements
evm.model.Contig371.14	CAAT-box	CAAAT	622	promoter-related elements
evm.model.Contig371.14	CAAT-box	CAAAT	634	promoter-related elements
evm.model.Contig371.14	CAAT-box	CAAT	643	promoter-related elements
evm.model.Contig371.14	CAAT-box	CAAT	667	promoter-related elements
evm.model.Contig371.14	CAAT-box	CAAAT	720	promoter-related elements
evm.model.Contig371.14	CAAT-box	CCAAT	762	promoter-related elements
evm.model.Contig371.14	CAAT-box	CAAT	763	promoter-related elements
evm.model.Contig371.14	CAAT-box	CAAAT	785	promoter-related elements
evm.model.Contig371.14	CAAT-box	CAAAT	813	promoter-related elements
evm.model.Contig371.14	CAAT-box	CAAAT	859	promoter-related elements
evm.model.Contig371.14	CAAT-box	CAAT	875	promoter-related elements
evm.model.Contig371.14	CAAT-box	CAAAT	990	promoter-related elements
evm.model.Contig371.14	CAAT-box	CAAT	997	promoter-related elements
evm.model.Contig371.14	CAAT-box	CAAAT	1003	promoter-related elements
evm.model.Contig371.14	CAAT-box	CAAAT	1051	promoter-related elements
evm.model.Contig371.14	CAAT-box	CCAAT	1080	promoter-related elements
evm.model.Contig371.14	CAAT-box	CAAT	1086	promoter-related elements
evm.model.Contig371.14	CAAT-box	CAAT	1110	promoter-related elements
evm.model.Contig371.14	CAAT-box	CAAAT	1118	promoter-related elements
evm.model.Contig371.14	CAAT-box	CAAAT	1130	promoter-related elements
evm.model.Contig371.14	CAAT-box	CAAAT	1198	promoter-related elements
evm.model.Contig371.14	CAAT-box	CAAAT	1210	promoter-related elements
evm.model.Contig371.14	CAAT-box	CAAT	1369	promoter-related elements
evm.model.Contig371.14	CAAT-box	CAAT	1408	promoter-related elements
evm.model.Contig371.14	CAAT-box	CAAT	1414	promoter-related elements
evm.model.Contig371.14	CAAT-box	CAAT	1428	promoter-related elements
evm.model.Contig371.14	CAAT-box	CCAAT	1439	promoter-related elements
evm.model.Contig371.14	CAAT-box	CAAT	1499	promoter-related elements
evm.model.Contig371.14	CAAT-box	CAAT	1540	promoter-related elements
evm.model.Contig371.14	CAAT-box	CAAT	1583	promoter-related elements
evm.model.Contig371.14	CAAT-box	CAAT	1604	promoter-related elements
evm.model.Contig371.14	CAAT-box	CAAT	1615	promoter-related elements
evm.model.Contig371.14	CAAT-box	CAAT	1630	promoter-related elements
evm.model.Contig371.14	CAAT-box	CAAAT	1649	promoter-related elements
evm.model.Contig371.14	CAAT-box	CCAAT	1652	promoter-related elements
evm.model.Contig371.14	CAAT-box	CAAAT	1657	promoter-related elements
evm.model.Contig371.14	CAAT-box	CAAT	1685	promoter-related elements

evm.model.Contig371.14	CAAT-box	CAAAT	1692	promoter-related elements
evm.model.Contig371.14	CAAT-box	CAAT	1721	promoter-related elements
evm.model.Contig371.14	CAAT-box	CCAAT	1913	promoter-related elements
evm.model.Contig371.14	CAAT-box	CAAT	1914	promoter-related elements
evm.model.Contig371.14	MYB	CAACAG	265	Site-binding related elements
evm.model.Contig371.14	LTR	CCGAAA	1251	environmental stress-related elements
evm.model.Contig371.14	MBS	CAACTG	334	Site-binding related elements
evm.model.Contig371.14	ACE	CTAACGTATT	291	light-responsive elements
evm.model.Contig371.14	MYC	CATTTG	487	Site-binding related elements
evm.model.Contig371.14	MYC	CATTTG	634	Site-binding related elements
evm.model.Contig371.14	MYC	CATTTG	1117	Site-binding related elements
evm.model.Contig371.14	MYC	CATTTG	1197	Site-binding related elements
evm.model.Contig371.14	MYC	CATTTG	1692	Site-binding related elements
evm.model.Contig371.14	WUN-motif	TAATTACTC	789	environmental stress-related elements
evm.model.Contig371.14	TCT-motif	TCTTAC	687	light-responsive elements
evm.model.Contig371.14	GA-motif	ATAGATAA	948	light-responsive elements
evm.model.Contig371.14	GA-motif	ATAGATAA	984	light-responsive elements
evm.model.Contig371.14	ARE	AAACCA	222	environmental stress-related elements
evm.model.Contig371.14	MYB recognition site	CCGTTG	95	Site-binding related elements
evm.model.Contig371.14	I-box	cCATATCCAAT	1652	light-responsive elements
evm.model.Contig371.14	W box	TTGACC	668	site-binding related elements
evm.model.Contig371.14	W box	TTGACC	1083	site-binding related elements
evm.model.Contig371.14	TATA-box	TATA	48	promoter-related elements
evm.model.Contig371.14	TATA-box	TATA	708	promoter-related elements
evm.model.Contig371.14	TATA-box	TATAAAT	765	promoter-related elements
evm.model.Contig371.14	TATA-box	TATAAA	766	promoter-related elements
evm.model.Contig371.14	TATA-box	TATAA	767	promoter-related elements
evm.model.Contig371.14	TATA-box	TATA	768	promoter-related elements
evm.model.Contig371.14	TATA-box	TATAAAA	1044	promoter-related elements
evm.model.Contig371.14	TATA-box	TATAAA	1045	promoter-related elements
evm.model.Contig371.14	TATA-box	TATAA	1046	promoter-related elements
evm.model.Contig371.14	TATA-box	TATA	1047	promoter-related elements
evm.model.Contig371.14	TATA-box	TACAAAA	1327	promoter-related elements
evm.model.Contig371.14	TATA-box	TATAAA	1558	promoter-related elements
evm.model.Contig371.14	TATA-box	TATAA	1559	promoter-related elements
evm.model.Contig371.14	TATA-box	TATA	1560	promoter-related elements
evm.model.Contig371.14	TATA-box	TATA	1601	promoter-related elements
evm.model.Contig371.14	TATA-box	ATTATA	1663	promoter-related elements
evm.model.Contig371.14	TATA-box	TATAA	1664	promoter-related elements
evm.model.Contig371.14	TATA-box	TATA	1665	promoter-related elements
evm.model.Contig371.14	TATA-box	TACATAAA	1667	promoter-related elements
evm.model.Contig371.14	TATA-box	TATACA	1865	promoter-related elements
evm.model.Contig371.14	TATA-box	TATA	1867	promoter-related elements
evm.model.Contig371.14	TATA-box	TATA	1983	promoter-related elements
evm.model.Contig371.14	L-box	ATCCCACCTAC	368	light-responsive elements
evm.model.Contig371.14	HD-Zip 3	GTAAT(G/C)ATTAC	153	Site-binding related elements
evm.model.Contig371.14	TATC-box	TATCCCA	367	homone-responsive elements
evm.model.Contig371.14	CCAAT-box	CAACGG	95	Site-binding related elements
evm.model.Contig371.14	Myb-binding site	CAACAG	265	Site-binding related elements
evm.model.Contig371.14	Myb	CAACTG	334	Site-binding related elements
evm.model.Contig371.14	G-box	CACGTG	1532	light-responsive elements
evm.model.Contig371.16	STRE	AGGGG	1691	environmental stress-related elements
evm.model.Contig371.16	GT1-motif	GGTTAA	549	light-responsive elements
evm.model.Contig371.16	GT1-motif	GGTTAA	870	light-responsive elements
evm.model.Contig371.16	Myb-binding site	CAACAG	1634	Site-binding related elements
evm.model.Contig371.16	CGTCA-motif	CGTCA	354	homone-responsive elements
evm.model.Contig371.16	CGTCA-motif	CGTCA	1336	homone-responsive elements
evm.model.Contig371.16	CGTCA-motif	CGTCA	1734	homone-responsive elements
evm.model.Contig371.16	Myb	TAACTG	471	Site-binding related elements
evm.model.Contig371.16	Myb	TAACTG	1302	Site-binding related elements
evm.model.Contig371.16	Myb	CAACTG	1413	Site-binding related elements
evm.model.Contig371.16	GARE-motif	TCTGTTG	1633	homone-responsive elements
evm.model.Contig371.16	LAMP-element	CTTTATCA	1082	light-responsive elements
evm.model.Contig371.16	MYB-like sequence	TAACCA	871	Site-binding related elements
evm.model.Contig371.16	MYB-like sequence	TAACCA	1480	Site-binding related elements
evm.model.Contig371.16	W box	TTGACC	1817	site-binding related elements
evm.model.Contig371.16	TATA-box	ATTATA	256	promoter-related elements
evm.model.Contig371.16	TATA-box	TATAA	257	promoter-related elements
evm.model.Contig371.16	TATA-box	TATA	258	promoter-related elements
evm.model.Contig371.16	TATA-box	TATA	284	promoter-related elements
evm.model.Contig371.16	TATA-box	ATATAA	364	promoter-related elements
evm.model.Contig371.16	TATA-box	TATA	365	promoter-related elements
evm.model.Contig371.16	TATA-box	ATTATA	452	promoter-related elements
evm.model.Contig371.16	TATA-box	TATAA	453	promoter-related elements
evm.model.Contig371.16	TATA-box	TATA	454	promoter-related elements
evm.model.Contig371.16	TATA-box	TATACA	583	promoter-related elements
evm.model.Contig371.16	TATA-box	TATA	585	promoter-related elements
evm.model.Contig371.16	TATA-box	ATATAT	698	promoter-related elements

evm.model.Contig371.16	TATA-box	TATA	699	promoter-related elements
evm.model.Contig371.16	TATA-box	TATA	755	promoter-related elements
evm.model.Contig371.16	TATA-box	TATA	809	promoter-related elements
evm.model.Contig371.16	TATA-box	ATATAT	950	promoter-related elements
evm.model.Contig371.16	TATA-box	TATA	951	promoter-related elements
evm.model.Contig371.16	TATA-box	ATATAT	1097	promoter-related elements
evm.model.Contig371.16	TATA-box	TATATA	1098	promoter-related elements
evm.model.Contig371.16	TATA-box	TATA	1100	promoter-related elements
evm.model.Contig371.16	TATA-box	ATTATA	1229	promoter-related elements
evm.model.Contig371.16	TATA-box	TATAA	1230	promoter-related elements
evm.model.Contig371.16	TATA-box	TATA	1231	promoter-related elements
evm.model.Contig371.16	TATA-box	ATATAT	1319	promoter-related elements
evm.model.Contig371.16	TATA-box	TATA	1320	promoter-related elements
evm.model.Contig371.16	TATA-box	TATAA	1544	promoter-related elements
evm.model.Contig371.16	TATA-box	TATA	1545	promoter-related elements
evm.model.Contig371.16	TATA-box	ATTATA	1789	promoter-related elements
evm.model.Contig371.16	TATA-box	TATAA	1790	promoter-related elements
evm.model.Contig371.16	TATA-box	TATA	1791	promoter-related elements
evm.model.Contig371.16	TATA-box	ATATAA	1807	promoter-related elements
evm.model.Contig371.16	TATA-box	TATA	1808	promoter-related elements
evm.model.Contig371.16	TATA-box	ATTATA	1978	promoter-related elements
evm.model.Contig371.16	TATA-box	TATAA	1979	promoter-related elements
evm.model.Contig371.16	TATA-box	TATA	1980	promoter-related elements
evm.model.Contig371.16	TATC-box	TATCCCA	675	homone-responsive elements
evm.model.Contig371.16	TCCC-motif	TCTCCCT	658	light-responsive elements
evm.model.Contig371.16	GATA-motif	AAGGATAAGG	1797	light-responsive elements
evm.model.Contig371.16	MYC	CATGTG	336	Site-binding related elements
evm.model.Contig371.16	MYC	CATTTG	687	Site-binding related elements
evm.model.Contig371.16	MYC	CATGTG	850	Site-binding related elements
evm.model.Contig371.16	TCA-element	CCATCTTTTT	1577	homone-responsive elements
evm.model.Contig371.16	TGACG-motif	TGACG	354	homone-responsive elements
evm.model.Contig371.16	TGACG-motif	TGACG	1336	homone-responsive elements
evm.model.Contig371.16	TGACG-motif	TGACG	1734	homone-responsive elements
evm.model.Contig371.16	MBS	CAACTG	1413	Site-binding related elements
evm.model.Contig371.16	MYB	CAACCA	347	Site-binding related elements
evm.model.Contig371.16	MYB	TAACCA	871	Site-binding related elements
evm.model.Contig371.16	MYB	TAACCA	1480	Site-binding related elements
evm.model.Contig371.16	MYB	CAACCA	1507	Site-binding related elements
evm.model.Contig371.16	MYB	CAACAG	1634	Site-binding related elements
evm.model.Contig371.16	LTR	CCGAAA	975	environmental stress-related elements
evm.model.Contig371.16	CAAT-box	CAAT	103	promoter-related elements
evm.model.Contig371.16	CAAT-box	CCAAT	126	promoter-related elements
evm.model.Contig371.16	CAAT-box	CAAAT	133	promoter-related elements
evm.model.Contig371.16	CAAT-box	CAAAT	140	promoter-related elements
evm.model.Contig371.16	CAAT-box	CCAAT	206	promoter-related elements
evm.model.Contig371.16	CAAT-box	CAAT	281	promoter-related elements
evm.model.Contig371.16	CAAT-box	CAAAT	292	promoter-related elements
evm.model.Contig371.16	CAAT-box	CAAT	297	promoter-related elements
evm.model.Contig371.16	CAAT-box	CAAAT	484	promoter-related elements
evm.model.Contig371.16	CAAT-box	CAAT	532	promoter-related elements
evm.model.Contig371.16	CAAT-box	CCAAT	537	promoter-related elements
evm.model.Contig371.16	CAAT-box	CAAT	538	promoter-related elements
evm.model.Contig371.16	CAAT-box	CAAT	623	promoter-related elements
evm.model.Contig371.16	CAAT-box	CCAAT	679	promoter-related elements
evm.model.Contig371.16	CAAT-box	CAAT	680	promoter-related elements
evm.model.Contig371.16	CAAT-box	CAAAT	688	promoter-related elements
evm.model.Contig371.16	CAAT-box	CAAT	822	promoter-related elements
evm.model.Contig371.16	CAAT-box	CAAAT	970	promoter-related elements
evm.model.Contig371.16	CAAT-box	CAAT	1064	promoter-related elements
evm.model.Contig371.16	CAAT-box	CAAT	1093	promoter-related elements
evm.model.Contig371.16	CAAT-box	CAAT	1188	promoter-related elements
evm.model.Contig371.16	CAAT-box	CAAT	1227	promoter-related elements
evm.model.Contig371.16	CAAT-box	CAAT	1371	promoter-related elements
evm.model.Contig371.16	CAAT-box	CAAAT	1554	promoter-related elements
evm.model.Contig371.16	CAAT-box	CAAAT	1576	promoter-related elements
evm.model.Contig371.16	CAAT-box	CAAAT	1603	promoter-related elements
evm.model.Contig371.16	CAAT-box	CAAAT	1622	promoter-related elements
evm.model.Contig371.16	CAAT-box	TGCCAAC	1636	promoter-related elements
evm.model.Contig371.16	CAAT-box	CAAT	1722	promoter-related elements
evm.model.Contig371.16	CAAT-box	CAAAT	1832	promoter-related elements
evm.model.Contig371.16	CAAT-box	CAAT	1838	promoter-related elements
evm.model.Contig371.16	CAAT-box	CAAAT	1840	promoter-related elements
evm.model.Contig371.16	CAAT-box	CAAAT	1873	promoter-related elements
evm.model.Contig371.16	CAAT-box	CAAT	1893	promoter-related elements
evm.model.Contig371.16	CAAT-box	CAAAT	1918	promoter-related elements
evm.model.Contig371.16	CAAT-box	CAAT	1963	promoter-related elements
evm.model.Contig371.16	Box 4	ATTAAT	269	light-responsive elements
evm.model.Contig371.16	Box 4	ATTAAT	1167	light-responsive elements

evm.model.Contig371.16	AuxRR-core	GGTCCAT	509	homone-responsive elements
evm.model.Contig371.16	TCT-motif	TCTTAC	23	light-responsive elements
evm.model.Contig371.16	TCT-motif	TCTTAC	1469	light-responsive elements
evm.model.Contig371.16	TCT-motif	TCTTAC	1939	light-responsive elements
evm.model.Contig371.16	I-box	ccttatcct	1798	light-responsive elements
evm.model.Contig371.16	ERE	ATTTCATA	149	homone-responsive elements
evm.model.Contig371.16	ERE	ATTTTAAA	194	homone-responsive elements
evm.model.Contig371.16	ERE	ATTTTAAA	1972	homone-responsive elements
evm.model.Contig371.16	ARE	AAACCA	1343	environmental stress-related elements
evm.model.Contig371.16	ARE	AAACCA	1443	environmental stress-related elements
evm.model.Contig371.16	P-box	CCTTTTG	110	homone-responsive elements
evm.model.Contig371.16	O2-site	GATGATGTGG	1742	other elements
evm.model.Contig394.265	ABRE	ACGTG	230	homone-responsive elements
evm.model.Contig394.265	G-Box	CACGTT	230	light-responsive elements
evm.model.Contig394.265	GCN4_motif	TGAGTCA	242	other elements
evm.model.Contig394.265	GCN4_motif	TGAGTCA	424	other elements
evm.model.Contig394.265	ERE	ATTTTAAA	639	homone-responsive elements
evm.model.Contig394.265	ERE	ATTTTAAA	1396	homone-responsive elements
evm.model.Contig394.265	ARE	AAACCA	1422	environmental stress-related elements
evm.model.Contig394.265	ARE	AAACCA	1447	environmental stress-related elements
evm.model.Contig394.265	MYC	CATTTG	601	Site-binding related elements
evm.model.Contig394.265	MYC	CATTTG	873	Site-binding related elements
evm.model.Contig394.265	MYC	CATTTG	1024	Site-binding related elements
evm.model.Contig394.265	MYC	CAATTG	1768	Site-binding related elements
evm.model.Contig394.265	TCA-element	TCAGAAGAGG	1887	homone-responsive elements
evm.model.Contig394.265	TGACG-motif	TGACG	769	homone-responsive elements
evm.model.Contig394.265	TGACG-motif	TGACG	1885	homone-responsive elements
evm.model.Contig394.265	LTR	CCGAAA	1162	environmental stress-related elements
evm.model.Contig394.265	ATC-motif	AGTAATCT	1092	light-responsive elements
evm.model.Contig394.265	MYB	CAACCA	553	Site-binding related elements
evm.model.Contig394.265	MYB	CAACCA	827	Site-binding related elements
evm.model.Contig394.265	MYB	CAACAG	1302	Site-binding related elements
evm.model.Contig394.265	CAAT-box	CAAT	50	promoter-related elements
evm.model.Contig394.265	CAAT-box	CAAAT	95	promoter-related elements
evm.model.Contig394.265	CAAT-box	CAAT	184	promoter-related elements
evm.model.Contig394.265	CAAT-box	CAAT	240	promoter-related elements
evm.model.Contig394.265	CAAT-box	CAAT	465	promoter-related elements
evm.model.Contig394.265	CAAT-box	CCAAT	497	promoter-related elements
evm.model.Contig394.265	CAAT-box	CAAAT	602	promoter-related elements
evm.model.Contig394.265	CAAT-box	CAAT	632	promoter-related elements
evm.model.Contig394.265	CAAT-box	CAAT	652	promoter-related elements
evm.model.Contig394.265	CAAT-box	CAAAT	666	promoter-related elements
evm.model.Contig394.265	CAAT-box	CAAT	691	promoter-related elements
evm.model.Contig394.265	CAAT-box	CAAT	726	promoter-related elements
evm.model.Contig394.265	CAAT-box	CAAT	758	promoter-related elements
evm.model.Contig394.265	CAAT-box	CAAAT	762	promoter-related elements
evm.model.Contig394.265	CAAT-box	CAAAT	772	promoter-related elements
evm.model.Contig394.265	CAAT-box	CCAAT	796	promoter-related elements
evm.model.Contig394.265	CAAT-box	CAAT	797	promoter-related elements
evm.model.Contig394.265	CAAT-box	CAAAT	873	promoter-related elements
evm.model.Contig394.265	CAAT-box	CAAT	897	promoter-related elements
evm.model.Contig394.265	CAAT-box	CAAT	912	promoter-related elements
evm.model.Contig394.265	CAAT-box	CAAT	987	promoter-related elements
evm.model.Contig394.265	CAAT-box	CAAAT	1025	promoter-related elements
evm.model.Contig394.265	CAAT-box	CAAAT	1066	promoter-related elements
evm.model.Contig394.265	CAAT-box	CCAAT	1078	promoter-related elements
evm.model.Contig394.265	CAAT-box	CAAT	1079	promoter-related elements
evm.model.Contig394.265	CAAT-box	CAAAT	1101	promoter-related elements
evm.model.Contig394.265	CAAT-box	CAAT	1106	promoter-related elements
evm.model.Contig394.265	CAAT-box	CAAT	1110	promoter-related elements
evm.model.Contig394.265	CAAT-box	CAAT	1133	promoter-related elements
evm.model.Contig394.265	CAAT-box	CAAT	1152	promoter-related elements
evm.model.Contig394.265	CAAT-box	CAAT	1193	promoter-related elements
evm.model.Contig394.265	CAAT-box	CAAT	1210	promoter-related elements
evm.model.Contig394.265	CAAT-box	CAAT	1285	promoter-related elements
evm.model.Contig394.265	CAAT-box	CAAT	1298	promoter-related elements
evm.model.Contig394.265	CAAT-box	CCAAT	1320	promoter-related elements
evm.model.Contig394.265	CAAT-box	CCAAT	1340	promoter-related elements
evm.model.Contig394.265	CAAT-box	CAAT	1368	promoter-related elements
evm.model.Contig394.265	CAAT-box	CAAAT	1454	promoter-related elements
evm.model.Contig394.265	CAAT-box	CAAT	1474	promoter-related elements
evm.model.Contig394.265	CAAT-box	CCAAT	1480	promoter-related elements
evm.model.Contig394.265	CAAT-box	CAAT	1481	promoter-related elements
evm.model.Contig394.265	CAAT-box	CAAT	1579	promoter-related elements
evm.model.Contig394.265	CAAT-box	CAAT	1674	promoter-related elements
evm.model.Contig394.265	CAAT-box	CAAT	1705	promoter-related elements
evm.model.Contig394.265	CAAT-box	CAAAT	1711	promoter-related elements
evm.model.Contig394.265	CAAT-box	CAAT	1716	promoter-related elements

evm.model.Contig394.265	CAAT-box	CCAAT	1743	promoter-related elements
evm.model.Contig394.265	CAAT-box	CAAT	1768	promoter-related elements
evm.model.Contig394.265	CAAT-box	CAAT	1770	promoter-related elements
evm.model.Contig394.265	CAAT-box	CAAT	1812	promoter-related elements
evm.model.Contig394.265	CAAT-box	CAAAT	1855	promoter-related elements
evm.model.Contig394.265	CAAT-box	CCAAT	1993	promoter-related elements
evm.model.Contig394.265	CAAT-box	CAAT	1994	promoter-related elements
evm.model.Contig394.265	Box 4	ATTAAT	446	light-responsive elements
evm.model.Contig394.265	Box 4	ATTAAT	1537	light-responsive elements
evm.model.Contig394.265	Box 4	ATTAAT	1821	light-responsive elements
evm.model.Contig394.265	TATA-box	TATA	40	promoter-related elements
evm.model.Contig394.265	TATA-box	ATTATA	56	promoter-related elements
evm.model.Contig394.265	TATA-box	TATAA	57	promoter-related elements
evm.model.Contig394.265	TATA-box	TATA	58	promoter-related elements
evm.model.Contig394.265	TATA-box	TATA	212	promoter-related elements
evm.model.Contig394.265	TATA-box	ATATAA	248	promoter-related elements
evm.model.Contig394.265	TATA-box	TATA	249	promoter-related elements
evm.model.Contig394.265	TATA-box	TATAAA	337	promoter-related elements
evm.model.Contig394.265	TATA-box	TATATAA	338	promoter-related elements
evm.model.Contig394.265	TATA-box	TATATA	339	promoter-related elements
evm.model.Contig394.265	TATA-box	ATATAA	340	promoter-related elements
evm.model.Contig394.265	TATA-box	TATA	341	promoter-related elements
evm.model.Contig394.265	TATA-box	TATTTAAA	370	promoter-related elements
evm.model.Contig394.265	TATA-box	TATA	544	promoter-related elements
evm.model.Contig394.265	TATA-box	TATAAAA	562	promoter-related elements
evm.model.Contig394.265	TATA-box	TATAAA	563	promoter-related elements
evm.model.Contig394.265	TATA-box	TATAA	564	promoter-related elements
evm.model.Contig394.265	TATA-box	TATA	565	promoter-related elements
evm.model.Contig394.265	TATA-box	ATATAT	591	promoter-related elements
evm.model.Contig394.265	TATA-box	TATATA	592	promoter-related elements
evm.model.Contig394.265	TATA-box	ATATAT	593	promoter-related elements
evm.model.Contig394.265	TATA-box	TATA	594	promoter-related elements
evm.model.Contig394.265	TATA-box	TATA	881	promoter-related elements
evm.model.Contig394.265	TATA-box	TATA	1070	promoter-related elements
evm.model.Contig394.265	TATA-box	TATAAAT	1402	promoter-related elements
evm.model.Contig394.265	TATA-box	TATAAA	1403	promoter-related elements
evm.model.Contig394.265	TATA-box	TATAA	1404	promoter-related elements
evm.model.Contig394.265	TATA-box	TATA	1405	promoter-related elements
evm.model.Contig394.265	TATA-box	TATAA	1830	promoter-related elements
evm.model.Contig394.265	TATA-box	TATA	1831	promoter-related elements
evm.model.Contig394.265	TATA-box	ATATAT	1868	promoter-related elements
evm.model.Contig394.265	TATA-box	TATA	1869	promoter-related elements
evm.model.Contig394.265	TATA-box	TATA	1959	promoter-related elements
evm.model.Contig394.265	GARE-motif	TCTGTTG	1301	homone-responsive elements
evm.model.Contig394.265	W box	TTGACC	510	site-binding related elements
evm.model.Contig394.265	W box	TTGACC	1789	site-binding related elements
evm.model.Contig394.265	CGTCA-motif	CGTCA	769	homone-responsive elements
evm.model.Contig394.265	CGTCA-motif	CGTCA	1885	homone-responsive elements
evm.model.Contig394.265	G-box	CACGAC	1626	light-responsive elements
evm.model.Contig394.265	Myb-binding site	CAACAG	1302	Site-binding related elements
evm.model.Contig394.265	STRE	AGGGG	1273	environmental stress-related elements
evm.model.Contig437.21	ARE	AAACCA	192	environmental stress-related elements
evm.model.Contig437.21	P-box	CCTTTTG	1475	homone-responsive elements
evm.model.Contig437.21	LTR	CCGAAA	1422	environmental stress-related elements
evm.model.Contig437.21	CAAT-box	CAAT	83	promoter-related elements
evm.model.Contig437.21	CAAT-box	CAAAT	96	promoter-related elements
evm.model.Contig437.21	CAAT-box	CAAT	112	promoter-related elements
evm.model.Contig437.21	CAAT-box	CAAT	132	promoter-related elements
evm.model.Contig437.21	CAAT-box	CCAAT	160	promoter-related elements
evm.model.Contig437.21	CAAT-box	CAAT	161	promoter-related elements
evm.model.Contig437.21	CAAT-box	CAAAT	207	promoter-related elements
evm.model.Contig437.21	CAAT-box	CCAAT	367	promoter-related elements
evm.model.Contig437.21	CAAT-box	CAAT	368	promoter-related elements
evm.model.Contig437.21	CAAT-box	CAAAT	372	promoter-related elements
evm.model.Contig437.21	CAAT-box	CAAAT	401	promoter-related elements
evm.model.Contig437.21	CAAT-box	CAAT	714	promoter-related elements
evm.model.Contig437.21	CAAT-box	CAAT	759	promoter-related elements
evm.model.Contig437.21	CAAT-box	CAAT	773	promoter-related elements
evm.model.Contig437.21	CAAT-box	CAAAT	821	promoter-related elements
evm.model.Contig437.21	CAAT-box	CCAAT	848	promoter-related elements
evm.model.Contig437.21	CAAT-box	CAAT	849	promoter-related elements
evm.model.Contig437.21	CAAT-box	CAAT	863	promoter-related elements
evm.model.Contig437.21	CAAT-box	CAAT	920	promoter-related elements
evm.model.Contig437.21	CAAT-box	CAAT	928	promoter-related elements
evm.model.Contig437.21	CAAT-box	CAAAT	998	promoter-related elements
evm.model.Contig437.21	CAAT-box	CAAAT	1144	promoter-related elements
evm.model.Contig437.21	CAAT-box	CAAT	1213	promoter-related elements
evm.model.Contig437.21	CAAT-box	CCAAT	1240	promoter-related elements

evm.model.Contig437.21	CAAT-box	CCAAT	1269	promoter-related elements
evm.model.Contig437.21	CAAT-box	CAAT	1383	promoter-related elements
evm.model.Contig437.21	CAAT-box	CAAT	1437	promoter-related elements
evm.model.Contig437.21	CAAT-box	CAAT	1463	promoter-related elements
evm.model.Contig437.21	CAAT-box	CCAAT	1499	promoter-related elements
evm.model.Contig437.21	CAAT-box	CAAT	1500	promoter-related elements
evm.model.Contig437.21	CAAT-box	CAAAT	1553	promoter-related elements
evm.model.Contig437.21	CAAT-box	CAAT	1559	promoter-related elements
evm.model.Contig437.21	CAAT-box	CAAT	1696	promoter-related elements
evm.model.Contig437.21	MYC	CATGTG	1225	Site-binding related elements
evm.model.Contig437.21	MYC	CATTTG	1552	Site-binding related elements
evm.model.Contig437.21	WUN-motif	TTATTACAT	854	environmental stress-related elements
evm.model.Contig437.21	TCA-element	CCATCTTTTT	1902	homone-responsive elements
evm.model.Contig437.21	TGACG-motif	TGACG	187	homone-responsive elements
evm.model.Contig437.21	TGACG-motif	TGACG	1408	homone-responsive elements
evm.model.Contig437.21	TGACG-motif	TGACG	1491	homone-responsive elements
evm.model.Contig437.21	ABRE	ACGTG	173	homone-responsive elements
evm.model.Contig437.21	ABRE	CGTACGTGCA	934	homone-responsive elements
evm.model.Contig437.21	ABRE	ACGTG	937	homone-responsive elements
evm.model.Contig437.21	AE-box	AGAAACAA	1951	light-responsive elements
evm.model.Contig437.21	CGTCA-motif	CGTCA	187	homone-responsive elements
evm.model.Contig437.21	CGTCA-motif	CGTCA	1408	homone-responsive elements
evm.model.Contig437.21	CGTCA-motif	CGTCA	1491	homone-responsive elements
evm.model.Contig437.21	G-box	CACGTC	172	light-responsive elements
evm.model.Contig437.21	G-box	TACGTG	936	light-responsive elements
evm.model.Contig437.21	Myb	TAACTG	720	Site-binding related elements
evm.model.Contig437.21	GT1-motif	GGTTAAT	1897	light-responsive elements
evm.model.Contig437.21	GT1-motif	GGTTAA	1898	light-responsive elements
evm.model.Contig437.21	TATC-box	TATCCCA	1872	homone-responsive elements
evm.model.Contig437.21	GATA-motif	GATAGGA	815	light-responsive elements
evm.model.Contig437.21	GATA-motif	GATAGGG	1364	light-responsive elements
evm.model.Contig437.21	CAT-box	GCCACT	977	other elements
evm.model.Contig437.21	CAT-box	GCCACT	1866	other elements
evm.model.Contig437.21	TATA-box	ATATAT	15	promoter-related elements
evm.model.Contig437.21	TATA-box	TATA	16	promoter-related elements
evm.model.Contig437.21	TATA-box	TATAA	36	promoter-related elements
evm.model.Contig437.21	TATA-box	TATA	37	promoter-related elements
evm.model.Contig437.21	TATA-box	ATATAA	324	promoter-related elements
evm.model.Contig437.21	TATA-box	TATA	325	promoter-related elements
evm.model.Contig437.21	TATA-box	TATA	395	promoter-related elements
evm.model.Contig437.21	TATA-box	ATATAT	443	promoter-related elements
evm.model.Contig437.21	TATA-box	TATATA	444	promoter-related elements
evm.model.Contig437.21	TATA-box	ATATAT	445	promoter-related elements
evm.model.Contig437.21	TATA-box	TATATA	446	promoter-related elements
evm.model.Contig437.21	TATA-box	ATATAT	447	promoter-related elements
evm.model.Contig437.21	TATA-box	TATATA	448	promoter-related elements
evm.model.Contig437.21	TATA-box	ATATAT	449	promoter-related elements
evm.model.Contig437.21	TATA-box	TATA	450	promoter-related elements
evm.model.Contig437.21	TATA-box	ATATAT	467	promoter-related elements
evm.model.Contig437.21	TATA-box	TATATA	468	promoter-related elements
evm.model.Contig437.21	TATA-box	TATA	470	promoter-related elements
evm.model.Contig437.21	TATA-box	ATATAT	475	promoter-related elements
evm.model.Contig437.21	TATA-box	TATATA	476	promoter-related elements
evm.model.Contig437.21	TATA-box	ATATAT	477	promoter-related elements
evm.model.Contig437.21	TATA-box	TATA	478	promoter-related elements
evm.model.Contig437.21	TATA-box	ATATAT	491	promoter-related elements
evm.model.Contig437.21	TATA-box	TATATA	492	promoter-related elements
evm.model.Contig437.21	TATA-box	TATA	494	promoter-related elements
evm.model.Contig437.21	TATA-box	ATATAT	509	promoter-related elements
evm.model.Contig437.21	TATA-box	TATATA	510	promoter-related elements
evm.model.Contig437.21	TATA-box	TATA	512	promoter-related elements
evm.model.Contig437.21	TATA-box	ATATAT	521	promoter-related elements
evm.model.Contig437.21	TATA-box	TATATA	522	promoter-related elements
evm.model.Contig437.21	TATA-box	ATATAT	523	promoter-related elements
evm.model.Contig437.21	TATA-box	TATA	524	promoter-related elements
evm.model.Contig437.21	TATA-box	ATATAT	541	promoter-related elements
evm.model.Contig437.21	TATA-box	TATATA	542	promoter-related elements
evm.model.Contig437.21	TATA-box	ATATAT	543	promoter-related elements
evm.model.Contig437.21	TATA-box	TATATA	544	promoter-related elements
evm.model.Contig437.21	TATA-box	ATATAT	545	promoter-related elements
evm.model.Contig437.21	TATA-box	TATATA	546	promoter-related elements
evm.model.Contig437.21	TATA-box	ATATAT	547	promoter-related elements
evm.model.Contig437.21	TATA-box	TATATA	548	promoter-related elements
evm.model.Contig437.21	TATA-box	ATATAT	549	promoter-related elements
evm.model.Contig437.21	TATA-box	TATATA	550	promoter-related elements
evm.model.Contig437.21	TATA-box	ATATAT	551	promoter-related elements
evm.model.Contig437.21	TATA-box	TATA	552	promoter-related elements
evm.model.Contig437.21	TATA-box	TATACA	564	promoter-related elements

evm.model.Contig437.21	TATA-box	TATATA	566	promoter-related elements
evm.model.Contig437.21	TATA-box	ATATAT	567	promoter-related elements
evm.model.Contig437.21	TATA-box	TATATA	568	promoter-related elements
evm.model.Contig437.21	TATA-box	ATATAT	569	promoter-related elements
evm.model.Contig437.21	TATA-box	TATATA	570	promoter-related elements
evm.model.Contig437.21	TATA-box	ATATAT	571	promoter-related elements
evm.model.Contig437.21	TATA-box	TATATA	572	promoter-related elements
evm.model.Contig437.21	TATA-box	ATATAT	573	promoter-related elements
evm.model.Contig437.21	TATA-box	TATATA	574	promoter-related elements
evm.model.Contig437.21	TATA-box	ATATAT	575	promoter-related elements
evm.model.Contig437.21	TATA-box	TATATA	576	promoter-related elements
evm.model.Contig437.21	TATA-box	ATATAT	577	promoter-related elements
evm.model.Contig437.21	TATA-box	TATATA	578	promoter-related elements
evm.model.Contig437.21	TATA-box	ATATAT	579	promoter-related elements
evm.model.Contig437.21	TATA-box	TATATA	580	promoter-related elements
evm.model.Contig437.21	TATA-box	TATA	582	promoter-related elements
evm.model.Contig437.21	TATA-box	ATATAT	591	promoter-related elements
evm.model.Contig437.21	TATA-box	TATATA	592	promoter-related elements
evm.model.Contig437.21	TATA-box	ATATAT	593	promoter-related elements
evm.model.Contig437.21	TATA-box	TATA	594	promoter-related elements
evm.model.Contig437.21	TATA-box	TATACA	598	promoter-related elements
evm.model.Contig437.21	TATA-box	TATATA	600	promoter-related elements
evm.model.Contig437.21	TATA-box	ATATAT	601	promoter-related elements
evm.model.Contig437.21	TATA-box	TATA	602	promoter-related elements
evm.model.Contig437.21	TATA-box	ATATAT	617	promoter-related elements
evm.model.Contig437.21	TATA-box	TATATA	618	promoter-related elements
evm.model.Contig437.21	TATA-box	ATATAT	619	promoter-related elements
evm.model.Contig437.21	TATA-box	TATATTTATATTT	620	promoter-related elements
evm.model.Contig437.21	TATA-box	taTATAAAtc	622	promoter-related elements
evm.model.Contig437.21	TATA-box	TATAAAT	623	promoter-related elements
evm.model.Contig437.21	TATA-box	TATAAA	624	promoter-related elements
evm.model.Contig437.21	TATA-box	TATATAA	625	promoter-related elements
evm.model.Contig437.21	TATA-box	TATATA	626	promoter-related elements
evm.model.Contig437.21	TATA-box	ATATAT	627	promoter-related elements
evm.model.Contig437.21	TATA-box	TATATA	628	promoter-related elements
evm.model.Contig437.21	TATA-box	ATATAT	629	promoter-related elements
evm.model.Contig437.21	TATA-box	TATATA	630	promoter-related elements
evm.model.Contig437.21	TATA-box	ATATAT	631	promoter-related elements
evm.model.Contig437.21	TATA-box	TATATA	632	promoter-related elements
evm.model.Contig437.21	TATA-box	ATATAT	633	promoter-related elements
evm.model.Contig437.21	TATA-box	TATATA	634	promoter-related elements
evm.model.Contig437.21	TATA-box	ATATAT	635	promoter-related elements
evm.model.Contig437.21	TATA-box	TATATA	636	promoter-related elements
evm.model.Contig437.21	TATA-box	ATATAT	637	promoter-related elements
evm.model.Contig437.21	TATA-box	TATA	638	promoter-related elements
evm.model.Contig437.21	TATA-box	TACAAAA	704	promoter-related elements
evm.model.Contig437.21	TATA-box	TATA	751	promoter-related elements
evm.model.Contig437.21	TATA-box	TATACA	775	promoter-related elements
evm.model.Contig437.21	TATA-box	TATATA	777	promoter-related elements
evm.model.Contig437.21	TATA-box	ATATAT	778	promoter-related elements
evm.model.Contig437.21	TATA-box	TATATA	779	promoter-related elements
evm.model.Contig437.21	TATA-box	ATATAT	780	promoter-related elements
evm.model.Contig437.21	TATA-box	TATA	781	promoter-related elements
evm.model.Contig437.21	TATA-box	ATATAA	915	promoter-related elements
evm.model.Contig437.21	TATA-box	TATA	916	promoter-related elements
evm.model.Contig437.21	TATA-box	tcTATAAATAgg	1087	promoter-related elements
evm.model.Contig437.21	TATA-box	TATA	1089	promoter-related elements
evm.model.Contig437.21	TATA-box	TATA	1318	promoter-related elements
evm.model.Contig437.21	TATA-box	ccTATAAAaa	1365	promoter-related elements
evm.model.Contig437.21	TATA-box	TATAA	1400	promoter-related elements
evm.model.Contig437.21	TATA-box	TATA	1401	promoter-related elements
evm.model.Contig437.21	TATA-box	TATA	1674	promoter-related elements
evm.model.Contig437.21	TATA-box	TATAA	1855	promoter-related elements
evm.model.Contig437.21	TATA-box	TATA	1856	promoter-related elements
evm.model.Contig437.21	TATA-box	TATA	1881	promoter-related elements
evm.model.Contig45.442	MBSI	TTTTTACGGTTA	1250	Site-binding related elements
evm.model.Contig45.442	chs-CMA1a	TTACTTAA	1710	light-responsive elements
evm.model.Contig45.442	AE-box	AGAAACTT	1239	light-responsive elements
evm.model.Contig45.442	TC-rich repeats	ATTCTCTAAC	1654	environmental stress-related elements
evm.model.Contig45.442	TC-rich repeats	ATTCTCTAAC	1849	environmental stress-related elements
evm.model.Contig45.442	ABRE	ACGTG	380	homone-responsive elements
evm.model.Contig45.442	TCT-motif	TCTTAC	1746	light-responsive elements
evm.model.Contig45.442	TCT-motif	TCTTAC	1811	light-responsive elements
evm.model.Contig45.442	Box 4	ATTAAT	672	light-responsive elements
evm.model.Contig45.442	Box 4	ATTAAT	758	light-responsive elements
evm.model.Contig45.442	CAAT-box	CAAT	39	promoter-related elements
evm.model.Contig45.442	CAAT-box	CAAAT	45	promoter-related elements
evm.model.Contig45.442	CAAT-box	CAAT	48	promoter-related elements

evm.model.Contig45.442	CAAT-box	CCAAT	142	promoter-related elements
evm.model.Contig45.442	CAAT-box	CAAT	143	promoter-related elements
evm.model.Contig45.442	CAAT-box	CAAAT	165	promoter-related elements
evm.model.Contig45.442	CAAT-box	CCAAT	213	promoter-related elements
evm.model.Contig45.442	CAAT-box	CAAT	214	promoter-related elements
evm.model.Contig45.442	CAAT-box	CAAAT	317	promoter-related elements
evm.model.Contig45.442	CAAT-box	CAAAT	669	promoter-related elements
evm.model.Contig45.442	CAAT-box	CAAAT	755	promoter-related elements
evm.model.Contig45.442	CAAT-box	CAAAT	791	promoter-related elements
evm.model.Contig45.442	CAAT-box	CAAT	815	promoter-related elements
evm.model.Contig45.442	CAAT-box	CAAAT	817	promoter-related elements
evm.model.Contig45.442	CAAT-box	CAAAT	859	promoter-related elements
evm.model.Contig45.442	CAAT-box	CAAAT	968	promoter-related elements
evm.model.Contig45.442	CAAT-box	CAAT	978	promoter-related elements
evm.model.Contig45.442	CAAT-box	CAAT	1005	promoter-related elements
evm.model.Contig45.442	CAAT-box	CAAT	1044	promoter-related elements
evm.model.Contig45.442	CAAT-box	CAAT	1127	promoter-related elements
evm.model.Contig45.442	CAAT-box	CAAT	1163	promoter-related elements
evm.model.Contig45.442	CAAT-box	CAAT	1183	promoter-related elements
evm.model.Contig45.442	CAAT-box	CAAT	1214	promoter-related elements
evm.model.Contig45.442	CAAT-box	CAAT	1291	promoter-related elements
evm.model.Contig45.442	CAAT-box	CCAAT	1320	promoter-related elements
evm.model.Contig45.442	CAAT-box	CAAT	1321	promoter-related elements
evm.model.Contig45.442	CAAT-box	CAAT	1343	promoter-related elements
evm.model.Contig45.442	CAAT-box	CAAT	1405	promoter-related elements
evm.model.Contig45.442	CAAT-box	CAAAT	1425	promoter-related elements
evm.model.Contig45.442	CAAT-box	CAAT	1472	promoter-related elements
evm.model.Contig45.442	CAAT-box	CAAT	1538	promoter-related elements
evm.model.Contig45.442	CAAT-box	CAAT	1560	promoter-related elements
evm.model.Contig45.442	CAAT-box	CAAAT	1578	promoter-related elements
evm.model.Contig45.442	CAAT-box	CAAT	1612	promoter-related elements
evm.model.Contig45.442	CAAT-box	CAAT	1755	promoter-related elements
evm.model.Contig45.442	CAAT-box	CAAT	1781	promoter-related elements
evm.model.Contig45.442	CAAT-box	CAAT	1857	promoter-related elements
evm.model.Contig45.442	CAAT-box	CAAT	1874	promoter-related elements
evm.model.Contig45.442	CAAT-box	CAAAT	1922	promoter-related elements
evm.model.Contig45.442	LTR	CCGAAA	1361	environmental stress-related elements
evm.model.Contig45.442	MYB	CAACCA	80	Site-binding related elements
evm.model.Contig45.442	MYB	CAACCA	247	Site-binding related elements
evm.model.Contig45.442	MBS	CAACTG	1352	Site-binding related elements
evm.model.Contig45.442	TGACG-motif	TGACG	602	homone-responsive elements
evm.model.Contig45.442	MYC	CATTTG	1577	Site-binding related elements
evm.model.Contig45.442	MYC	CATGTG	1722	Site-binding related elements
evm.model.Contig45.442	MYC	CATGTG	1770	Site-binding related elements
evm.model.Contig45.442	MYC	CATGTG	1935	Site-binding related elements
evm.model.Contig45.442	WUN-motif	AAATTACT	1312	environmental stress-related elements
evm.model.Contig45.442	ARE	AAACCA	1348	environmental stress-related elements
evm.model.Contig45.442	ARE	AAACCA	1503	environmental stress-related elements
evm.model.Contig45.442	ERE	ATTTTAAA	395	homone-responsive elements
evm.model.Contig45.442	ERE	ATTTTAAA	397	homone-responsive elements
evm.model.Contig45.442	ERE	ATTTCATA	797	homone-responsive elements
evm.model.Contig45.442	ERE	ATTTTAAA	980	homone-responsive elements
evm.model.Contig45.442	ERE	ATTTCATA	1308	homone-responsive elements
evm.model.Contig45.442	ERE	ATTTCATA	1525	homone-responsive elements
evm.model.Contig45.442	TGA-element	AACGAC	1190	homone-responsive elements
evm.model.Contig45.442	TATA-box	TATACA	357	promoter-related elements
evm.model.Contig45.442	TATA-box	TATA	359	promoter-related elements
evm.model.Contig45.442	TATA-box	TATACA	480	promoter-related elements
evm.model.Contig45.442	TATA-box	TATA	482	promoter-related elements
evm.model.Contig45.442	TATA-box	TATACA	576	promoter-related elements
evm.model.Contig45.442	TATA-box	TATA	578	promoter-related elements
evm.model.Contig45.442	TATA-box	taTATAAAtc	638	promoter-related elements
evm.model.Contig45.442	TATA-box	ATATAT	643	promoter-related elements
evm.model.Contig45.442	TATA-box	TATATA	644	promoter-related elements
evm.model.Contig45.442	TATA-box	ATATAA	645	promoter-related elements
evm.model.Contig45.442	TATA-box	TATA	646	promoter-related elements
evm.model.Contig45.442	TATA-box	TACAAAA	840	promoter-related elements
evm.model.Contig45.442	TATA-box	TACATAAA	849	promoter-related elements
evm.model.Contig45.442	TATA-box	TATA	877	promoter-related elements
evm.model.Contig45.442	TATA-box	TATATA	913	promoter-related elements
evm.model.Contig45.442	TATA-box	TATA	915	promoter-related elements
evm.model.Contig45.442	TATA-box	TATAAAA	1106	promoter-related elements
evm.model.Contig45.442	TATA-box	TATAAA	1107	promoter-related elements
evm.model.Contig45.442	TATA-box	TATAA	1108	promoter-related elements
evm.model.Contig45.442	TATA-box	TATA	1109	promoter-related elements
evm.model.Contig45.442	TATA-box	TATAA	1194	promoter-related elements
evm.model.Contig45.442	TATA-box	TATA	1195	promoter-related elements
evm.model.Contig45.442	TATA-box	TATA	1675	promoter-related elements

evm.model.Contig45.442	TATA-box	ATTATA	1802	promoter-related elements
evm.model.Contig45.442	TATA-box	TATATAA	1803	promoter-related elements
evm.model.Contig45.442	TATA-box	TATATA	1804	promoter-related elements
evm.model.Contig45.442	TATA-box	TATA	1806	promoter-related elements
evm.model.Contig45.442	TATA-box	TATA	1882	promoter-related elements
evm.model.Contig45.442	TATA-box	TATTTAAA	1897	promoter-related elements
evm.model.Contig45.442	TATA-box	TATA	1990	promoter-related elements
evm.model.Contig45.442	W box	TTGACC	551	site-binding related elements
evm.model.Contig45.442	W box	TTGACC	924	site-binding related elements
evm.model.Contig45.442	W box	TTGACC	975	site-binding related elements
evm.model.Contig45.442	GATA-motif	GATAGGG	54	light-responsive elements
evm.model.Contig45.442	GATA-motif	AAGGATAAGG	688	light-responsive elements
evm.model.Contig45.442	chs-CMA2a	TCACTTGA	1154	light-responsive elements
evm.model.Contig45.442	STRE	AGGGG	265	environmental stress-related elements
evm.model.Contig45.442	Myb	TAACTG	302	Site-binding related elements
evm.model.Contig45.442	Myb	CAACTG	1352	Site-binding related elements
evm.model.Contig45.442	G-box	CACGTC	380	light-responsive elements
evm.model.Contig45.442	CGTCA-motif	CGTCA	602	homone-responsive elements
evm.model.Contig480.228	Myc	TCTCTTA	341	Site-binding related elements
evm.model.Contig480.228	TATA-box	ATATAA	67	promoter-related elements
evm.model.Contig480.228	TATA-box	TATA	68	promoter-related elements
evm.model.Contig480.228	TATA-box	TATA	131	promoter-related elements
evm.model.Contig480.228	TATA-box	TATA	775	promoter-related elements
evm.model.Contig480.228	TATA-box	TATA	837	promoter-related elements
evm.model.Contig480.228	TATA-box	ATATAT	907	promoter-related elements
evm.model.Contig480.228	TATA-box	TATATA	908	promoter-related elements
evm.model.Contig480.228	TATA-box	TATA	910	promoter-related elements
evm.model.Contig480.228	TATA-box	TATA	927	promoter-related elements
evm.model.Contig480.228	TATA-box	TATAAAT	1029	promoter-related elements
evm.model.Contig480.228	TATA-box	TATAAA	1030	promoter-related elements
evm.model.Contig480.228	TATA-box	TATAA	1031	promoter-related elements
evm.model.Contig480.228	TATA-box	TATA	1032	promoter-related elements
evm.model.Contig480.228	TATA-box	TATA	1170	promoter-related elements
evm.model.Contig480.228	TATA-box	TATA	1263	promoter-related elements
evm.model.Contig480.228	TATA-box	TATAAAA	1318	promoter-related elements
evm.model.Contig480.228	TATA-box	TATAAA	1319	promoter-related elements
evm.model.Contig480.228	TATA-box	TATAA	1320	promoter-related elements
evm.model.Contig480.228	TATA-box	TATA	1321	promoter-related elements
evm.model.Contig480.228	TATA-box	TATA	1331	promoter-related elements
evm.model.Contig480.228	TATA-box	ATATAA	1366	promoter-related elements
evm.model.Contig480.228	TATA-box	TATA	1367	promoter-related elements
evm.model.Contig480.228	TATA-box	TACATAAA	1412	promoter-related elements
evm.model.Contig480.228	TATA-box	TATA	1424	promoter-related elements
evm.model.Contig480.228	TATA-box	ATATAT	1433	promoter-related elements
evm.model.Contig480.228	TATA-box	TATA	1434	promoter-related elements
evm.model.Contig480.228	TATA-box	TATAAA	1785	promoter-related elements
evm.model.Contig480.228	TATA-box	TATAA	1786	promoter-related elements
evm.model.Contig480.228	TATA-box	TATA	1787	promoter-related elements
evm.model.Contig480.228	TATA-box	TATA	1875	promoter-related elements
evm.model.Contig480.228	TATA-box	TATATA	1920	promoter-related elements
evm.model.Contig480.228	TATA-box	ATATAT	1921	promoter-related elements
evm.model.Contig480.228	TATA-box	TATATA	1922	promoter-related elements
evm.model.Contig480.228	TATA-box	TATA	1924	promoter-related elements
evm.model.Contig480.228	TATA-box	ccTATAAAaa	1974	promoter-related elements
evm.model.Contig480.228	MYB-like sequence	TAACCA	1957	Site-binding related elements
evm.model.Contig480.228	CAT-box	GCCACT	716	other elements
evm.model.Contig480.228	GATA-motif	GATAGGA	607	light-responsive elements
evm.model.Contig480.228	GT1-motif	GGTTAAT	155	light-responsive elements
evm.model.Contig480.228	GT1-motif	GGTTAA	156	light-responsive elements
evm.model.Contig480.228	GT1-motif	GGTTAAT	535	light-responsive elements
evm.model.Contig480.228	GT1-motif	GGTTAAT	733	light-responsive elements
evm.model.Contig480.228	GT1-motif	GGTTAA	734	light-responsive elements
evm.model.Contig480.228	GT1-motif	GGTTAA	866	light-responsive elements
evm.model.Contig480.228	Myb	TAACTG	177	Site-binding related elements
evm.model.Contig480.228	Myb	TAACTG	616	Site-binding related elements
