# Supplementary material for: Genome-wide identification and expression pattern analysis of the SABATH gene family in Neolamarckia cadamba
Source: For Res (Fayettev). 2023 May 29;3:13. doi: 10.48130/FR-2023-0013 (PMC11524262; doi:10.48130/FR-2023-0013)
Supplement: Supplementary file 1 — Supplementary data to this article can be found online. [file FR-2023-0013-S1.zip › 10.48130_FR-2023-0013-Suppl-TableS5.pdf]

Table S5. Predicted Transcription Factor binding sites in the promoters of *NcSABATHs* .

Transcription Factor ID	Family	Query Sequence Name	Start	Stop	Strand	Score	p-value	q-value	Matched Sequence
AT4G38910	BBR-BPC	NcSABATH11	1962	1991	+	48.9367	8.99E-17	8.00E-12	agagagacagagagagagagggagagagag
AT4G38910	BBR-BPC	NcSABATH11	1958	1987	+	44.5949	5.30E-16	2.35E-11	agagagagagacagagagagagagggagag
AT4G38910	BBR-BPC	NcSABATH11	1964	1993	+	43.0633	1.27E-15	2.37E-11	agagacagagagagagagggagagagagag
AT4G38910	BBR-BPC	NcSABATH11	1966	1995	+	43.038	1.31E-15	2.37E-11	agacagagagagagagggagagagagagag
AT4G38910	BBR-BPC	NcSABATH11	1960	1989	+	43.0253	1.33E-15	2.37E-11	agagagagacagagagagagagggagagag
AT4G38910	BBR-BPC	NcSABATH11	1968	1997	+	38.1139	1.03E-14	1.52E-10	acagagagagagagggagagagagagagac
AT2G01930	BBR-BPC	NcSABATH11	1971	1994	+	38.8906	1.11E-13	4.66E-09	gagagagagagggagagagagaga
AT2G01930	BBR-BPC	NcSABATH11	1969	1992	+	38.7344	1.24E-13	4.66E-09	cagagagagagagggagagagaga
AT2G01930	BBR-BPC	NcSABATH11	1973	1996	+	38.3125	1.80E-13	4.66E-09	gagagagagggagagagagagaga
AT2G01930	BBR-BPC	NcSABATH11	1967	1990	+	38.125	2.13E-13	4.66E-09	gacagagagagagagggagagaga
AT2G01930	BBR-BPC	NcSABATH11	1963	1986	+	37.7812	2.82E-13	4.94E-09	gagagacagagagagagagggaga
AT4G38910	BBR-BPC	NcSABATH11	1970	1999	+	28.3038	5.25E-13	6.66E-09	agagagagagagggagagagagagagacga
AT5G42520	BBR-BPC	NcSABATH16	1019	1039	-	37.0822	5.54E-13	4.91E-08	ATCTCTCTCTCTCTCTCTCTC
AT2G01930	BBR-BPC	NcSABATH11	1975	1998	+	36.1094	1.14E-12	1.67E-08	gagagagggagagagagagagacg
AT4G38910	BBR-BPC	NcSABATH11	1956	1985	+	24.6329	1.88E-12	2.09E-08	acagagagagagacagagagagagagggag
AT2G01930	BBR-BPC	NcSABATH11	1957	1980	+	35.1406	2.22E-12	2.59E-08	cagagagagagacagagagagaga
AT2G01930	BBR-BPC	NcSABATH11	1959	1982	+	35	2.44E-12	2.59E-08	gagagagagacagagagagagagg
AT2G01930	BBR-BPC	NcSABATH11	1965	1988	+	34.8594	2.66E-12	2.59E-08	gagacagagagagagagggagaga
AT5G42520	BBR-BPC	NcSABATH11	1971	1991	-	34.0137	4.90E-12	1.45E-07	CTCTCTCTCCCTCTCTCTCTC
AT5G42520	BBR-BPC	NcSABATH11	1975	1995	-	34.0137	4.90E-12	1.45E-07	CTCTCTCTCTCTCCCTCTCTC
AT5G42520	BBR-BPC	NcSABATH16	1017	1037	-	32.7671	1.36E-11	3.01E-07	CTCTCTCTCTCTCTCTCTCGA
AT5G42520	BBR-BPC	NcSABATH16	1021	1041	-	32.4658	1.72E-11	3.04E-07	ATATCTCTCTCTCTCTCTCTC
AT5G42520	BBR-BPC	NcSABATH11	1965	1985	-	31.9452	2.40E-11	3.54E-07	CTCCCTCTCTCTCTCTGTCTC
AT2G01930	BBR-BPC	NcSABATH16	1019	1042	+	30.6406	4.65E-11	4.08E-07	gagagagagagagagagagatatt
AT2G01930	BBR-BPC	NcSABATH16	1017	1040	+	29.6406	8.63E-11	6.88E-07	tcgagagagagagagagagagata
AT5G42520	BBR-BPC	NcSABATH11	1969	1989	-	29.726	1.00E-10	1.23E-06	CTCTCTCCCTCTCTCTCTCTG
AT5G42520	BBR-BPC	NcSABATH11	1977	1997	-	29.5753	1.11E-10	1.23E-06	GTCTCTCTCTCTCTCCCTCTC
AT4G38910	BBR-BPC	NcSABATH11	1952	1981	+	11.9367	1.12E-10	1.11E-06	aagcacagagagagagacagagagagagag
AT2G01930	BBR-BPC	NcSABATH11	1977	2000	+	29.0312	1.26E-10	9.23E-07	gagagggagagagagagagacgag
AT2G01930	BBR-BPC	NcSABATH11	1961	1984	+	27.9688	2.42E-10	1.63E-06	gagagagacagagagagagaggga
AT4G38910	BBR-BPC	NcSABATH11	1954	1983	+	9.27848	2.50E-10	2.22E-06	gcacagagagagagacagagagagagaggg
AT5G42520	BBR-BPC	NcSABATH11	1973	1993	-	28.0959	2.86E-10	2.82E-06	CTCTCTCTCTCCCTCTCTCTC
AT3G50410	Dof	NcSABATH3	1226	1246	-	18.9375	3.19E-10	2.86E-05	CTTCTTCTTTTTTTCCTTTTT
AT5G42520	BBR-BPC	NcSABATH11	1979	1999	-	27.4384	4.20E-10	3.46E-06	TCGTCTCTCTCTCTCTCCCTC
AT5G42520	BBR-BPC	NcSABATH11	1959	1979	-	27.2603	4.66E-10	3.46E-06	CTCTCTCTCTGTCTCTCTCTC
AT5G42520	BBR-BPC	NcSABATH11	1957	1977	-	27.1644	4.94E-10	3.46E-06	CTCTCTCTGTCTCTCTCTCTG
AT5G42520	BBR-BPC	NcSABATH11	1961	1981	-	27.1096	5.08E-10	3.46E-06	CTCTCTCTCTCTGTCTCTCTC
AT3G26790	AP2/ERF	NcSABATH4	1278	1292	+	20.6094	5.55E-10	5.03E-05	acatgcatgcatgca
AT5G38860	bHLH	NcSABATH23	750	767	+	25.1233	6.62E-10	3.02E-05	acgtgatactcacgtgcc
AT5G38860	bHLH	NcSABATH19	752	769	+	25.1233	6.62E-10	3.02E-05	acgtgatactcacgtgcc
AT5G62470	MYB	NcSABATH11	1882	1899	-	22.25	7.08E-10	6.44E-05	GGTGGTAGGTGGTAGGTG
AT5G66940	Dof	NcSABATH3	1219	1247	-	21.1094	1.18E-09	0.000105	TCTTCTTCTTTTTTTCCTTTTTTTACTTT
AT5G42520	BBR-BPC	NcSABATH11	1963	1983	-	25.2466	1.44E-09	9.14E-06	CCCTCTCTCTCTCTGTCTCTC
AT5G38860	bHLH	NcSABATH23	747	764	-	24.5479	1.45E-09	3.29E-05	ACGTGAGTATCACGTGAC
AT5G38860	bHLH	NcSABATH19	749	766	-	24.5479	1.45E-09	3.29E-05	ACGTGAGTATCACGTGAC
AT2G45660	MIKC_MADS	NcSABATH16	1018	1038	-	20.0725	1.52E-09	0.000135	TCTCTCTCTCTCTCTCTCTCG
AT5G42520	BBR-BPC	NcSABATH16	1023	1043	-	24.8493	1.79E-09	1.06E-05	AAATATCTCTCTCTCTCTCTC
AT4G38910	BBR-BPC	NcSABATH11	1950	1979	+	2	1.89E-09	1.53E-05	gcaagcacagagagagagacagagagagag
AT2G01930	BBR-BPC	NcSABATH16	1021	1044	+	23.4531	3.03E-09	1.90E-05	gagagagagagagagagatattta
AT4G38000	Dof	NcSABATH3	1225	1252	-	20.9219	3.05E-09	0.000268	TGTTTTCTTCTTCTTTTTTTCCTTTTTT
AT3G26790	AP2/ERF	NcSABATH4	1274	1288	+	19.8594	3.59E-09	0.000163	acatacatgcatgca
AT2G45660	MIKC_MADS	NcSABATH11	1974	1994	-	19.7391	4.04E-09	0.000179	TCTCTCTCTCTCCCTCTCTCT
AT4G38910	BBR-BPC	NcSABATH16	1016	1045	+	-1.06329	4.26E-09	3.16E-05	atcgagagagagagagagagagagatatttat
AT2G01930	BBR-BPC	NcSABATH11	1953	1976	+	22.7031	4.50E-09	2.63E-05	agcacagagagagagacagagaga
AT1G74650	MYB	NcSABATH11	1882	1896	+	20.7812	4.58E-09	0.000416	cacctaccacctacc
AT1G69570	Dof	NcSABATH3	1714	1740	+	21.125	5.31E-09	0.000474	atattgcttttccttttttttttttt
AT5G66940	Dof	NcSABATH18	1449	1477	-	20.25	5.41E-09	0.000241	TCCTCTTACCTTTTTTCTTTTTTTTTTCC
AT2G28810	Dof	NcSABATH3	1225	1245	-	18.8906	5.75E-09	0.000516	TTCTTCTTTTTTTCCTTTTTT
AT2G45660	MIKC_MADS	NcSABATH11	1960	1980	-	19.5797	6.07E-09	0.000179	TCTCTCTCTCTGTCTCTCTCT
AT5G02460	Dof	NcSABATH3	1225	1245	+	19.5156	7.36E-09	0.000657	aaaaaaggaaaaaaagaagaa
AT1G10120	bHLH	NcSABATH23	743	763	-	21.9315	8.16E-09	0.000371	CGTGAGTATCACGTGACCTGA
AT1G10120	bHLH	NcSABATH19	745	765	-	21.9315	8.16E-09	0.000371	CGTGAGTATCACGTGACCTGA
AT5G17430	AP2/ERF	NcSABATH8	1835	1854	-	20.4684	8.42E-09	0.000748	AAAAAAAAAAAAAAAGAGACA
AT5G42520	BBR-BPC	NcSABATH11	1967	1987	-	21.7945	8.85E-09	4.90E-05	CTCTCCCTCTCTCTCTCTGTC
AT2G01930	BBR-BPC	NcSABATH16	1015	1038	+	21.0625	1.04E-08	5.50E-05	tatcgagagagagagagagagaga

AT4G38910	BBR-BPC	NcSABATH11	1948	1977	+	-4.65823	1.05E-08	7.21E-05	gggcaagcacagagagagagacagagagag
AT2G01930	BBR-BPC	NcSABATH11	1955	1978	+	21.0156	1.07E-08	5.50E-05	cacagagagagagacagagagaga
AT3G26790	AP2/ERF	NcSABATH20	815	829	-	19.2969	1.18E-08	0.000358	ATATACATGCATGCA
AT4G26640	WRKY	NcSABATH5	1229	1241	+	17.6562	1.52E-08	0.00139	gccgttgaccagt
AT2G45660	MIKC_MADS	NcSABATH11	1958	1978	-	19.1159	1.79E-08	0.000398	TCTCTCTCTGTCTCTCTCTCT
AT1G01720	NAC	NcSABATH13	680	701	+	18.625	1.79E-08	0.00163	tacgtgttgaattcgtcatcta
AT5G42520	BBR-BPC	NcSABATH16	1015	1035	-	20.1233	2.00E-08	0.000104	CTCTCTCTCTCTCTCTCGATA
AT5G62940	Dof	NcSABATH3	1720	1740	-	17.8281	2.12E-08	0.00191	AAAAAAAAAAAAAAAGGAAAAG
AT2G01570	GRAS	NcSABATH11	1959	1978	+	19.3143	2.35E-08	0.0021	gagagagagacagagagaga
AT3G55370	Dof	NcSABATH18	1452	1472	-	18.875	2.49E-08	0.00134	TTACCTTTTTTCTTTTTTTTT
AT2G45660	MIKC_MADS	NcSABATH11	1956	1976	-	18.9275	2.67E-08	0.000473	TCTCTCTGTCTCTCTCTCTGT
AT3G49690	MYB	NcSABATH11	1881	1893	-	22.4051	2.76E-08	0.00126	AGGTGGTAGGTGG
AT3G49690	MYB	NcSABATH11	1888	1900	-	22.4051	2.76E-08	0.00126	AGGTGGTAGGTGG
AT5G38860	bHLH	NcSABATH23	1117	1134	+	21.726	3.07E-08	0.000467	atgtgagtatcacgtgac
AT5G38860	bHLH	NcSABATH19	1117	1134	+	21.726	3.07E-08	0.000467	atgtgagtatcacgtgac
AT2G45660	MIKC_MADS	NcSABATH11	1972	1992	-	18.7826	3.53E-08	0.000486	TCTCTCTCTCCCTCTCTCTCT
AT1G69570	Dof	NcSABATH17	219	245	-	19.5781	3.82E-08	0.00161	CTTTCACTTTCTCTTTTTTGGTTTCTT
AT2G45660	MIKC_MADS	NcSABATH11	1968	1988	-	18.7391	3.84E-08	0.000486	TCTCTCCCTCTCTCTCTCTGT
AT5G62470	MYB	NcSABATH7	31	48	-	19.75	3.87E-08	0.00176	GTAGGTAGGTGGTGGATG
AT3G55370	Dof	NcSABATH17	820	840	-	18.5781	3.95E-08	0.00134	TTAGCTTTTTGCTTTTTTTTT
AT1G35460	bHLH	NcSABATH1	1874	1884	-	18.5156	4.55E-08	0.00412	TGCAAGTGGCC
AT3G55370	Dof	NcSABATH7	1329	1349	+	18.4531	4.79E-08	0.00134	ttttctttttcttttttccc
AT5G17430	AP2/ERF	NcSABATH16	1019	1038	+	19.3671	4.85E-08	0.00148	gagagagagagagagagaga
AT4G38910	BBR-BPC	NcSABATH16	1012	1041	+	-11	4.89E-08	0.000311	tactatcgagagagagagagagagagagatat
AT2G45660	MIKC_MADS	NcSABATH8	1835	1855	+	18.5942	5.02E-08	0.000556	tgtctcttttttttttttttt
AT3G26790	AP2/ERF	NcSABATH20	812	826	+	18.5312	5.36E-08	0.00121	atatgcatgcatgta
AT3G45610	Dof	NcSABATH17	822	840	-	17.2344	5.39E-08	0.00484	TTAGCTTTTTGCTTTTTTT
AT3G50060	MYB	NcSABATH8	525	539	+	16.2031	5.89E-08	0.00538	ctggggtgacggtta
AT1G69570	Dof	NcSABATH18	1448	1474	-	19.1875	6.04E-08	0.00161	TCTTACCTTTTTTCTTTTTTTTTTCCT
AT3G55370	Dof	NcSABATH3	1224	1244	-	18.2969	6.06E-08	0.00134	TCTTCTTTTTTTCCTTTTTTT
AT1G29160	Dof	NcSABATH3	1720	1740	-	17.5312	6.34E-08	0.00553	AAAAAAAAAAAAAAAGGAAAAG
AT1G69010	bHLH	NcSABATH23	749	763	+	20.125	6.67E-08	0.002	cacgtgatactcacg
AT1G69010	bHLH	NcSABATH19	751	765	+	20.125	6.67E-08	0.002	cacgtgatactcacg
AT5G62940	Dof	NcSABATH7	1327	1347	-	17.2188	6.82E-08	0.00216	GAAAAAAAGAAAAAGAAAACA
AT2G42280	bHLH	NcSABATH1	1874	1884	+	17.9589	6.84E-08	0.00623	ggccacttgca
AT5G17430	AP2/ERF	NcSABATH11	1971	1990	+	19.0886	7.03E-08	0.00148	gagagagagagggagagaga
AT5G62940	Dof	NcSABATH18	1454	1474	+	17.1875	7.20E-08	0.00216	aaaaaaagaaaaaaggtaaga
AT3G47600	MYB	NcSABATH7	32	50	-	18.9844	7.81E-08	0.00708	GGGTAGGTAGGTGGTGGAT
AT2G45660	MIKC_MADS	NcSABATH1	403	423	+	18.3043	8.29E-08	0.000747	ttttcttttctttcttttctt
AT2G45660	MIKC_MADS	NcSABATH7	1330	1350	+	18.2754	8.70E-08	0.000747	tttctttttctttttttcccc
AT1G69010	bHLH	NcSABATH23	751	765	-	19.875	8.78E-08	0.002	CACGTGAGTATCACG
AT1G69010	bHLH	NcSABATH19	753	767	-	19.875	8.78E-08	0.002	CACGTGAGTATCACG
AT1G22640	MYB	NcSABATH7	40	50	-	17.4681	9.10E-08	0.00416	GGGTAGGTAGG
AT1G22640	MYB	NcSABATH7	283	293	-	17.4681	9.10E-08	0.00416	GGGTAGGTAGG
AT4G38910	BBR-BPC	NcSABATH16	1018	1047	+	-13.7722	9.49E-08	0.000562	cgagagagagagagagagagagatatttatta
AT5G62940	Dof	NcSABATH17	822	842	+	17.0156	9.76E-08	0.0022	aaaaaaagcaaaaagctaata
AT1G10120	bHLH	NcSABATH23	751	771	+	19.726	1.05E-07	0.00237	cgtgatactcacgtgccaata
AT1G10120	bHLH	NcSABATH19	753	773	+	19.726	1.05E-07	0.00237	cgtgatactcacgtgccaata
AT1G51700	Dof	NcSABATH3	1717	1737	-	16.6875	1.05E-07	0.00938	AAAAAAAAAAAGGAAAAGCAA
AT2G45660	MIKC_MADS	NcSABATH11	1962	1982	-	18.1594	1.05E-07	0.000747	CCTCTCTCTCTCTGTCTCTCT
AT2G45660	MIKC_MADS	NcSABATH11	1978	1998	-	18.1594	1.05E-07	0.000747	CGTCTCTCTCTCTCTCCCTCT
AT4G38910	BBR-BPC	NcSABATH16	1020	1049	+	-14.2532	1.06E-07	0.000587	agagagagagagagagagagatatttattatt
AT1G69570	Dof	NcSABATH9	1868	1894	+	18.6875	1.06E-07	0.00161	atttttctttttttttttccctttcgtt
AT1G69570	Dof	NcSABATH10	1868	1894	+	18.6875	1.06E-07	0.00161	atttttctttttttttttccctttcgtt
AT1G69570	Dof	NcSABATH18	1441	1467	-	18.6719	1.08E-07	0.00161	TTTTTTCTTTTTTTTTTCCTGGTTTTC
AT1G49480	AP2/ERF	NcSABATH12	249	263	+	18.3088	1.10E-07	0.00516	accaaaaaaaaaaaa
AT5G17430	AP2/ERF	NcSABATH11	1973	1992	+	18.7215	1.11E-07	0.00148	gagagagagggagagagaga
AT4G38000	Dof	NcSABATH3	1216	1243	-	18.25	1.12E-07	0.00492	CTTCTTTTTTTTCCTTTTTTTACTTTAAT
AT5G17430	AP2/ERF	NcSABATH10	1869	1888	-	18.7089	1.13E-07	0.00148	AGGGAAAAAAAAAAAGAAAAA
AT5G17430	AP2/ERF	NcSABATH9	1869	1888	-	18.7089	1.13E-07	0.00148	AGGGAAAAAAAAAAAGAAAAA
AT2G45660	MIKC_MADS	NcSABATH3	1720	1740	+	18.1014	1.15E-07	0.000747	cttttccttttttttttttt
AT5G17430	AP2/ERF	NcSABATH7	1330	1349	-	18.6835	1.17E-07	0.00148	GGGAAAAAAAGAAAAAGAAA
AT2G01570	GRAS	NcSABATH16	1019	1038	+	18.1286	1.19E-07	0.0048	gagagagagagagagagaga
AT2G45660	MIKC_MADS	NcSABATH11	1966	1986	-	18.058	1.23E-07	0.000747	TCTCCCTCTCTCTCTCTGTCT
AT1G29160	Dof	NcSABATH21	1777	1797	-	17.1094	1.25E-07	0.00553	AAAACAATAAAAAAGTGAAAG
AT1G25340	MYB	NcSABATH16	1173	1187	+	17.3529	1.25E-07	0.0114	aaaagaagttaggca
AT2G45660	MIKC_MADS	NcSABATH3	1229	1249	-	18.0435	1.26E-07	0.000747	TTTCTTCTTCTTTTTTTCCTT

AT4G38910	BBR-BPC	NcSABATH16	1010	1039	+	-15.4304	1.39E-07	0.000725	gttactatcgagagagagagagagagagagat
AT1G74650	MYB	NcSABATH7	31	45	+	18.4844	1.40E-07	0.00633	catccaccacctacc
AT3G26790	AP2/ERF	NcSABATH20	811	825	-	17.9531	1.43E-07	0.00259	ACATGCATGCATATA
AT3G55370	Dof	NcSABATH1	403	423	+	17.6875	1.44E-07	0.00203	ttttcttttctttcttttctt
AT3G12720	MYB	NcSABATH7	40	60	-	18.1094	1.44E-07	0.0131	GGTTGGAAGAGGGTAGGTAGG
AT3G10580	MYB_related	NcSABATH18	1794	1808	+	16.973	1.56E-07	0.0142	agtaaggataaggat
AT1G10120	bHLH	NcSABATH23	1118	1138	+	19.3014	1.60E-07	0.00242	tgtgagtatcacgtgaccgga
AT1G10120	bHLH	NcSABATH19	1118	1138	+	19.3014	1.60E-07	0.00242	tgtgagtatcacgtgaccgga
AT1G51140	bHLH	NcSABATH1	1872	1885	+	17.4219	1.73E-07	0.0158	acggccacttgcat
AT2G45660	MIKC_MADS	NcSABATH3	1224	1244	-	17.8261	1.76E-07	0.000975	TCTTCTTTTTTTCCTTTTTTT
AT5G02460	Dof	NcSABATH18	1455	1475	+	17.5781	1.79E-07	0.008	aaaaaagaaaaaaggtaagag
AT3G55370	Dof	NcSABATH9	1870	1890	+	17.5156	1.80E-07	0.00203	ttttcttttttttttcccttt
AT3G55370	Dof	NcSABATH10	1870	1890	+	17.5156	1.80E-07	0.00203	ttttcttttttttttcccttt
AT3G55370	Dof	NcSABATH16	271	291	+	17.5	1.84E-07	0.00203	ttttcttttttcatttttctt
AT2G45660	MIKC_MADS	NcSABATH7	1328	1348	+	17.7826	1.87E-07	0.000977	gttttctttttctttttttcc
AT2G01930	BBR-BPC	NcSABATH17	883	906	-	14.7656	2.00E-07	0.000975	AAGAGAGAGAGGATGAGGGAAATA
AT1G49480	AP2/ERF	NcSABATH8	1841	1855	-	17.9853	2.02E-07	0.00516	AAAAAAAAAAAAAAA
AT5G17430	AP2/ERF	NcSABATH8	1837	1856	-	18.2278	2.02E-07	0.00224	CAAAAAAAAAAAAAAAGAGA
AT2G45660	MIKC_MADS	NcSABATH7	1031	1051	-	17.7101	2.08E-07	0.00102	TTTCTTTTTCTTTGTCTCCTT
AT4G30980	bHLH	NcSABATH23	751	765	+	19.4302	2.09E-07	0.00952	cgtgatactcacgtg
AT4G30980	bHLH	NcSABATH19	753	767	+	19.4302	2.09E-07	0.00952	cgtgatactcacgtg
AT3G26790	AP2/ERF	NcSABATH4	1277	1291	-	17.7188	2.13E-07	0.00322	GCATGCATGCATGTA
AT1G79180	MYB	NcSABATH11	1880	1900	+	18.8594	2.16E-07	0.0196	accacctaccacctaccacct
AT2G01570	GRAS	NcSABATH11	1977	1996	+	17.6286	2.17E-07	0.0048	gagagggagagagagagaga
AT2G45660	MIKC_MADS	NcSABATH8	1837	1857	+	17.6667	2.21E-07	0.00103	tctctttttttttttttttgg
AT1G49480	AP2/ERF	NcSABATH2	1681	1695	-	17.9118	2.23E-07	0.00516	CACAAAAAAAAAAAA
AT5G42520	BBR-BPC	NcSABATH11	1955	1975	-	14.6027	2.32E-07	0.00114	CTCTCTGTCTCTCTCTCTGTG
AT5G17430	AP2/ERF	NcSABATH11	1979	1998	+	18.1013	2.34E-07	0.00231	gagggagagagagagagacg
AT3G45610	Dof	NcSABATH18	1454	1472	-	16.3906	2.45E-07	0.011	TTACCTTTTTTCTTTTTTT
AT3G28910	MYB	NcSABATH11	1885	1899	+	17.8594	2.46E-07	0.0224	ctaccacctaccacc
AT4G24020	Nin-like	NcSABATH12	1353	1367	+	16.0938	2.55E-07	0.0233	agttgacttttcggg
AT5G66940	Dof	NcSABATH23	1072	1100	+	17.4688	2.61E-07	0.0074	aaattttcccttatttcttttttttttt
AT1G64620	Dof	NcSABATH3	1720	1738	-	15.7812	2.63E-07	0.0138	AAAAAAAAAAAAGGAAAAG
AT2G01570	GRAS	NcSABATH11	1967	1986	+	17.4571	2.65E-07	0.0048	gacagagagagagagggaga
AT2G01570	GRAS	NcSABATH7	1028	1047	+	17.4429	2.69E-07	0.0048	tagaaggagacaaagaaaaa
AT4G38910	BBR-BPC	NcSABATH16	1014	1043	+	-18.481	2.69E-07	0.00133	ctatcgagagagagagagagagagatattt
AT3G13810	C2H2	NcSABATH7	1029	1049	-	17.4375	2.70E-07	0.0242	TCTTTTTCTTTGTCTCCTTCT
AT2G45660	MIKC_MADS	NcSABATH18	1448	1468	-	17.5072	2.76E-07	0.00122	CTTTTTTCTTTTTTTTTTCCT
AT1G49480	AP2/ERF	NcSABATH2	1680	1694	-	17.7059	2.90E-07	0.00516	ACAAAAAAAAAAAAA
AT3G47600	MYB	NcSABATH11	1883	1901	-	17.9219	2.92E-07	0.0132	CAGGTGGTAGGTGGTAGGT
AT3G55370	Dof	NcSABATH8	1836	1856	+	17.1094	3.02E-07	0.0024	gtctctttttttttttttttg
AT5G01380	Trihelix	NcSABATH14	607	623	-	17.6765	3.03E-07	0.0275	GACACGTGTGTAATATT
AT2G45660	MIKC_MADS	NcSABATH7	1029	1049	-	17.4348	3.05E-07	0.00128	TCTTTTTCTTTGTCTCCTTCT
AT3G55370	Dof	NcSABATH3	1722	1742	+	17.0938	3.08E-07	0.0024	tttcctttttttttttttaa
AT1G64620	Dof	NcSABATH18	1456	1474	+	15.7031	3.09E-07	0.0138	aaaaagaaaaaaggtaaga
AT3G55370	Dof	NcSABATH3	1716	1736	+	17.0781	3.14E-07	0.0024	attgcttttcctttttttttt
AT1G49480	AP2/ERF	NcSABATH8	1844	1858	-	17.6324	3.16E-07	0.00516	CCCAAAAAAAAAAAA
AT2G45660	MIKC_MADS	NcSABATH18	1454	1474	-	17.4058	3.17E-07	0.00128	TCTTACCTTTTTTCTTTTTTT
AT3G26790	AP2/ERF	NcSABATH4	1282	1296	+	17.4219	3.28E-07	0.00424	gcatgcatgcaaaca
AT5G17430	AP2/ERF	NcSABATH11	1977	1996	+	17.7975	3.30E-07	0.00293	gagagggagagagagagaga
AT1G19210	AP2/ERF	NcSABATH10	919	932	-	18.1719	3.38E-07	0.0154	ATGGACGGTGACAG
AT1G19210	AP2/ERF	NcSABATH9	919	932	-	18.1719	3.38E-07	0.0154	ATGGACGGTGACAG
AT3G12720	MYB	NcSABATH11	1880	1900	-	17.4844	3.40E-07	0.0155	AGGTGGTAGGTGGTAGGTGGT
AT5G42520	BBR-BPC	NcSABATH23	923	943	-	13.6301	3.45E-07	0.00161	TTCATTCCCTCTCTCTCTTAA
AT3G55370	Dof	NcSABATH23	1009	1029	+	16.9844	3.53E-07	0.0024	tttactttttttatttgtttt
AT3G55370	Dof	NcSABATH19	1011	1031	+	16.9844	3.53E-07	0.0024	tttactttttttatttgtttt
AT3G48430	C2H2	NcSABATH12	269	280	-	18.726	3.54E-07	0.0318	AAAAACAGAGGA
AT1G73360	HD-ZIP	NcSABATH9	666	676	-	16.6562	3.54E-07	0.0161	GCATTAAATGC
AT1G73360	HD-ZIP	NcSABATH10	667	677	-	16.6562	3.54E-07	0.0161	GCATTAAATGC
AT3G11280	MYB	NcSABATH18	1795	1808	-	15.9062	3.62E-07	0.0326	ATCCTTATCCTTAC
AT1G06180	MYB	NcSABATH18	343	357	+	17.7812	3.82E-07	0.0296	gaagtggttggtgac
AT3G06490	MYB	NcSABATH12	116	130	+	15.8594	3.87E-07	0.0354	ccggggaagttaggc
AT5G66940	Dof	NcSABATH11	1295	1323	+	17.125	3.87E-07	0.0074	atattttcttttttttctttagcgtcttt
AT3G12820	MYB	NcSABATH11	1880	1895	-	17.6562	3.95E-07	0.0293	GTAGGTGGTAGGTGGT
AT1G68320	MYB	NcSABATH16	1174	1188	+	16	4.01E-07	0.0366	aaagaagttaggcac
AT5G62470	MYB	NcSABATH11	1878	1895	-	17.6094	4.06E-07	0.00857	GTAGGTGGTAGGTGGTTG
AT5G12870	MYB	NcSABATH7	37	46	-	15.6058	4.08E-07	0.0207	AGGTAGGTGG

AT1G49480	AP2/ERF	NcSABATH2	1679	1693	-	17.3088	4.09E-07	0.00516	CAAAAAAAAAAAAAA
AT1G49480	AP2/ERF	NcSABATH8	1842	1856	-	17.3088	4.09E-07	0.00516	CAAAAAAAAAAAAAA
AT5G62470	MYB	NcSABATH11	1875	1892	-	17.5938	4.12E-07	0.00857	GGTGGTAGGTGGTTGGTT
AT5G66940	Dof	NcSABATH17	817	845	-	17.0625	4.16E-07	0.0074	TTTTATTAGCTTTTTGCTTTTTTTTTAAA
AT2G01930	BBR-BPC	NcSABATH16	1023	1046	+	13.0625	4.16E-07	0.00192	gagagagagagagagatatttatt
AT5G42520	BBR-BPC	NcSABATH17	888	908	+	13.1507	4.17E-07	0.00185	ccctcatcctctctctcttaa
AT4G21030	Dof	NcSABATH18	1458	1476	+	16.75	4.18E-07	0.0374	aaagaaaaaaggtaagagg
AT3G55370	Dof	NcSABATH18	1453	1473	-	16.8125	4.34E-07	0.00274	CTTACCTTTTTTCTTTTTTTT
AT4G38910	BBR-BPC	NcSABATH7	1971	2000	+	-20.7722	4.45E-07	0.00208	aaacacacggagaaagtgtaagagggagat
AT5G14340	MYB	NcSABATH7	40	52	+	16.3529	4.51E-07	0.0206	cctacctaccctc
AT5G14340	MYB	NcSABATH7	283	295	+	16.3529	4.51E-07	0.0206	cctacctaccctc
AT2G04880	WRKY	NcSABATH2	99	109	+	18.5814	4.53E-07	0.0414	ttgaccgagca
AT3G26790	AP2/ERF	NcSABATH16	433	447	+	17.2031	4.64E-07	0.00526	acatacatgcatata
AT5G62470	MYB	NcSABATH7	35	52	-	17.4531	4.71E-07	0.00857	GAGGGTAGGTAGGTGGTG
AT1G64620	Dof	NcSABATH17	824	842	+	15.4844	4.71E-07	0.0141	aaaaagcaaaaagctaata
AT5G42520	BBR-BPC	NcSABATH7	401	421	-	12.8219	4.75E-07	0.00201	CCTGCTTTCTCTCTATCTCTG
AT2G01930	BBR-BPC	NcSABATH13	553	576	-	12.6719	4.90E-07	0.00215	ATGAGAGGGAGGGAGAGTAACAGC
AT2G01570	GRAS	NcSABATH11	1975	1994	+	16.8714	5.06E-07	0.0075	gagagagggagagagagaga
AT2G22750	bHLH	NcSABATH23	747	763	-	18.0274	5.09E-07	0.0232	CGTGAGTATCACGTGAC
AT2G22750	bHLH	NcSABATH19	749	765	-	18.0274	5.09E-07	0.0232	CGTGAGTATCACGTGAC
AT1G09540	MYB	NcSABATH11	1880	1894	+	16.7812	5.17E-07	0.025	accacctaccaccta
AT5G02460	Dof	NcSABATH3	1226	1246	+	16.75	5.19E-07	0.00889	aaaaaggaaaaaaagaagaag
AT1G79180	MYB	NcSABATH18	344	364	-	17.8281	5.23E-07	0.0226	TTACATCGTCACCAACCACTT
AT5G17430	AP2/ERF	NcSABATH3	1230	1249	+	17.3544	5.29E-07	0.00397	aggaaaaaaagaagaagaaa
AT5G46590	NAC	NcSABATH4	1101	1116	+	17.6562	5.30E-07	0.0484	cttgaaaatgaagaaa
AT5G17430	AP2/ERF	NcSABATH18	1447	1466	+	17.3418	5.37E-07	0.00397	caggaaaaaaaaaagaaaaa
AT3G23240	AP2/ERF	NcSABATH23	1618	1645	-	17.75	5.42E-07	0.0246	TTGGTTTCGCCGCCATGGTTTCTGTTTC
AT3G23240	AP2/ERF	NcSABATH19	1618	1645	-	17.75	5.42E-07	0.0246	TTGGTTTCGCCGCCATGGTTTCTGTTTC
AT5G49330	MYB	NcSABATH7	41	50	-	16.3269	5.45E-07	0.0187	GGGTAGGTAG
AT5G49330	MYB	NcSABATH7	284	293	-	16.3269	5.45E-07	0.0187	GGGTAGGTAG
AT2G25000	WRKY	NcSABATH5	1230	1240	-	16.8617	5.47E-07	0.0501	CTGGTCAACGG
AT1G69010	bHLH	NcSABATH23	1118	1132	-	17.9531	5.78E-07	0.0088	CACGTGATACTCACA
AT1G69010	bHLH	NcSABATH19	1118	1132	-	17.9531	5.78E-07	0.0088	CACGTGATACTCACA
AT1G69570	Dof	NcSABATH23	1007	1033	+	17.0469	5.80E-07	0.00621	tttttactttttttatttgtttttgag
AT1G69570	Dof	NcSABATH19	1009	1035	+	17.0469	5.80E-07	0.00621	tttttactttttttatttgtttttgag
AT5G02460	Dof	NcSABATH1	407	427	-	16.6562	5.80E-07	0.00889	TGAAAAGAAAAGAAAGAAAAG
AT1G72050	C2H2	NcSABATH7	1737	1755	-	17.1092	5.86E-07	0.0524	CCACTACCTCCTCTTTCAC
AT1G49480	AP2/ERF	NcSABATH8	1843	1857	-	17.0294	5.92E-07	0.0055	CCAAAAAAAAAAAAA
AT2G45660	MIKC_MADS	NcSABATH3	1223	1243	-	16.913	5.93E-07	0.00229	CTTCTTTTTTTCCTTTTTTTA
AT3G14180	Trihelix	NcSABATH13	35	45	+	17.8906	6.01E-07	0.055	ccgccggaaaa
AT5G02460	Dof	NcSABATH9	1872	1892	-	16.625	6.02E-07	0.00889	CGAAAGGGAAAAAAAAAAGAA
AT5G02460	Dof	NcSABATH10	1872	1892	-	16.625	6.02E-07	0.00889	CGAAAGGGAAAAAAAAAAGAA
AT5G17430	AP2/ERF	NcSABATH13	744	763	+	17.2025	6.18E-07	0.00422	cataaagaaagaaaaagagg
AT1G69570	Dof	NcSABATH7	1327	1353	+	16.9688	6.26E-07	0.00621	tgttttctttttcttttttttccccctaa
AT3G26790	AP2/ERF	NcSABATH4	1281	1295	-	16.9688	6.36E-07	0.00641	GTTTGCATGCATGCA
AT3G12820	MYB	NcSABATH7	40	55	-	17.2344	6.43E-07	0.0293	GAAGAGGGTAGGTAGG
AT5G08130	bHLH	NcSABATH23	749	762	+	17.75	6.44E-07	0.0146	cacgtgatactcac
AT5G08130	bHLH	NcSABATH19	751	764	+	17.75	6.44E-07	0.0146	cacgtgatactcac
AT5G08130	bHLH	NcSABATH19	1119	1132	-	17.75	6.44E-07	0.0146	CACGTGATACTCAC
AT5G08130	bHLH	NcSABATH23	1119	1132	-	17.75	6.44E-07	0.0146	CACGTGATACTCAC
AT2G01570	GRAS	NcSABATH11	1981	2000	+	16.6286	6.54E-07	0.0075	gggagagagagagagacgag
AT2G01570	GRAS	NcSABATH11	1963	1982	+	16.6	6.73E-07	0.0075	gagagacagagagagagagg
AT4G38000	Dof	NcSABATH7	1318	1345	+	16.6094	6.74E-07	0.0184	aatttctagtgttttctttttcttttttt
AT5G12870	MYB	NcSABATH7	41	50	-	15.3365	6.79E-07	0.0207	GGGTAGGTAG
AT5G12870	MYB	NcSABATH7	284	293	-	15.3365	6.79E-07	0.0207	GGGTAGGTAG
AT3G01970	WRKY	NcSABATH12	240	251	-	17.2812	6.80E-07	0.0622	GGTTGACTTTTT
AT2G45660	MIKC_MADS	NcSABATH13	745	765	-	16.7971	6.81E-07	0.00242	CTCCTCTTTTTCTTTCTTTAT
AT2G45660	MIKC_MADS	NcSABATH18	1447	1467	-	16.7971	6.81E-07	0.00242	TTTTTTCTTTTTTTTTTCCTG
AT2G01930	BBR-BPC	NcSABATH11	1951	1974	+	11.8438	6.90E-07	0.00288	caagcacagagagagagacagaga
AT5G02460	Dof	NcSABATH17	823	843	+	16.5	6.98E-07	0.00889	aaaaaagcaaaaagctaataa
AT1G09540	MYB	NcSABATH7	36	50	+	16.5312	7.01E-07	0.025	accacctacctaccc
AT5G63260	C3H	NcSABATH5	1811	1821	+	13.8281	7.01E-07	0.0641	gaaaaagtgaa
AT3G08500	MYB	NcSABATH18	346	356	+	16.3906	7.05E-07	0.064	gtggttggtga
AT1G49480	AP2/ERF	NcSABATH22	579	593	+	16.8382	7.06E-07	0.0055	aacaaaaaaaaaaga
AT5G52170	HD-ZIP	NcSABATH9	666	676	+	15.8406	7.08E-07	0.0322	gcatttaatgc
AT5G52170	HD-ZIP	NcSABATH10	667	677	+	15.8406	7.08E-07	0.0322	gcatttaatgc
AT3G23250	MYB	NcSABATH11	1877	1897	+	16.7344	7.13E-07	0.0352	ccaaccacctaccacctacca

AT5G45300	BES1	NcSABATH14	21	35	+	17.9375	7.16E-07	0.0654	ccacacgtgtctcaa
AT1G08810	MYB	NcSABATH18	340	358	-	17.0312	7.22E-07	0.0656	CGTCACCAACCACTTCTCA
AT1G69570	Dof	NcSABATH18	1449	1475	-	16.8125	7.27E-07	0.00649	CTCTTACCTTTTTTCTTTTTTTTTTCC
AT3G12720	MYB	NcSABATH22	1106	1126	-	16.875	7.30E-07	0.0221	GTTGGAAAAGAGGTAGGTGCA
AT1G49480	AP2/ERF	NcSABATH2	1678	1692	-	16.8235	7.48E-07	0.0055	AAAAAAAAAAAAAAG
AT1G49480	AP2/ERF	NcSABATH3	1726	1740	-	16.8235	7.48E-07	0.0055	AAAAAAAAAAAAAAG
AT1G49480	AP2/ERF	NcSABATH8	1840	1854	-	16.8235	7.48E-07	0.0055	AAAAAAAAAAAAAAG
AT1G51700	Dof	NcSABATH3	1220	1240	+	15.4688	7.53E-07	0.018	aagtaaaaaaaggaaaaaaag
AT5G14960	E2F/DP	NcSABATH6	187	206	-	15.5938	7.60E-07	0.0688	AGTTGATTATGGAGGGAAAA
AT2G01930	BBR-BPC	NcSABATH12	1457	1480	+	11.5938	7.64E-07	0.00305	gagtgagagagaagtattgcaaga
AT5G17430	AP2/ERF	NcSABATH3	1720	1739	-	16.9873	7.65E-07	0.00425	AAAAAAAAAAAAAAGGAAAAG
AT3G45610	Dof	NcSABATH3	1722	1740	+	15.6406	7.65E-07	0.0229	tttccttttttttttttt
AT1G51700	Dof	NcSABATH17	825	845	+	15.4531	7.71E-07	0.018	aaaagcaaaaagctaataaaa
AT3G23250	MYB	NcSABATH11	1870	1890	+	16.6406	7.82E-07	0.0352	cttaaaaccaaccacctacca
AT5G17430	AP2/ERF	NcSABATH2	1675	1694	-	16.962	7.83E-07	0.00425	ACAAAAAAAAAAAAAAAGAAG
AT5G17430	AP2/ERF	NcSABATH18	1450	1469	+	16.962	7.83E-07	0.00425	gaaaaaaaaaagaaaaaagg
AT1G51700	Dof	NcSABATH3	1229	1249	+	15.4219	8.07E-07	0.018	aaggaaaaaaagaagaagaaa
AT5G01380	Trihelix	NcSABATH14	614	630	+	16.9412	8.13E-07	0.0369	cacacgtgtctcaagtt
AT5G17430	AP2/ERF	NcSABATH7	1045	1064	+	16.9241	8.14E-07	0.00425	aaagaaataaatgagagaaa
AT5G49330	MYB	NcSABATH11	1881	1890	-	16.1827	8.16E-07	0.0187	TGGTAGGTGG
AT5G49330	MYB	NcSABATH11	1888	1897	-	16.1827	8.16E-07	0.0187	TGGTAGGTGG
AT5G22570	WRKY	NcSABATH5	1230	1239	+	16.9658	8.16E-07	0.0748	ccgttgacca
AT2G01930	BBR-BPC	NcSABATH16	1013	1036	+	11.4219	8.20E-07	0.00313	actatcgagagagagagagagaga
AT2G45660	MIKC_MADS	NcSABATH10	1870	1890	+	16.6377	8.20E-07	0.00269	ttttcttttttttttcccttt
AT2G45660	MIKC_MADS	NcSABATH9	1870	1890	+	16.6377	8.20E-07	0.00269	ttttcttttttttttcccttt
AT1G09540	MYB	NcSABATH11	1887	1901	+	16.3906	8.27E-07	0.025	accacctaccacctg
AT4G30980	bHLH	NcSABATH23	760	774	-	17.3023	8.36E-07	0.0191	GGTTATTGGCACGTG
AT4G30980	bHLH	NcSABATH19	762	776	-	17.3023	8.36E-07	0.0191	GGTTATTGGCACGTG
AT4G38000	Dof	NcSABATH3	1226	1253	-	16.3906	8.40E-07	0.0184	ATGTTTTCTTCTTCTTTTTTTCCTTTTT
AT2G18380	GATA	NcSABATH5	431	443	+	15.4531	8.47E-07	0.0771	tggatctgattgg
AT2G01570	GRAS	NcSABATH11	1965	1984	+	16.3714	8.50E-07	0.00842	gagacagagagagagaggga
AT1G06180	MYB	NcSABATH11	1880	1894	-	17.0938	8.60E-07	0.0296	TAGGTGGTAGGTGGT
AT2G45660	MIKC_MADS	NcSABATH1	401	421	+	16.5942	8.62E-07	0.00273	ggttttcttttcttcttttc
AT4G14770	CPP	NcSABATH18	193	207	+	16.8382	8.66E-07	0.0781	ttttaaaatttaaat
AT5G42520	BBR-BPC	NcSABATH16	1025	1045	-	11.274	8.67E-07	0.00341	ATAAATATCTCTCTCTCTCTC
AT5G26170	WRKY	NcSABATH12	239	251	-	16.8594	8.70E-07	0.0796	GGTTGACTTTTTG
AT3G22780	CPP	NcSABATH18	192	206	+	16.411	8.85E-07	0.0797	gttttaaaatttaaa
AT5G42520	BBR-BPC	NcSABATH17	886	906	+	11.2192	8.86E-07	0.00341	ttccctcatcctctctctctt
AT1G20980	SBP	NcSABATH7	581	591	+	15.6875	8.90E-07	0.0814	tccgtacactt
AT3G61250	MYB	NcSABATH11	1880	1894	-	16.7812	8.99E-07	0.0297	TAGGTGGTAGGTGGT
AT5G22990	C2H2	NcSABATH12	589	603	+	16.0781	9.14E-07	0.0835	tcgacttcgagttcg
AT2G01930	BBR-BPC	NcSABATH7	1026	1049	+	11.0938	9.36E-07	0.00342	tatagaaggagacaaagaaaaaga
AT3G47600	MYB	NcSABATH17	366	384	-	16.8125	9.48E-07	0.0287	GTAAGGGTAGTTGGGATAT
AT5G46350	WRKY	NcSABATH12	239	253	+	17.4062	9.55E-07	0.0865	caaaaagtcaaccaa
AT3G45150	TCP	NcSABATH15	1527	1537	+	17.6962	9.73E-07	0.0889	atcgggtccac
AT3G61250	MYB	NcSABATH7	40	54	-	16.7031	9.76E-07	0.0297	AAGAGGGTAGGTAGG
AT3G61250	MYB	NcSABATH7	283	297	-	16.7031	9.76E-07	0.0297	AAGAGGGTAGGTAGG
AT5G08130	bHLH	NcSABATH17	1524	1537	-	17.3438	9.78E-07	0.0177	CACGTGCCATTTCC
AT3G46070	C2H2	NcSABATH12	1454	1468	-	15.8082	9.78E-07	0.0887	TCTCTCTCACTCCAA
AT5G42520	BBR-BPC	NcSABATH13	554	574	+	10.9452	9.82E-07	0.00363	ctgttactctccctccctctc
AT1G06180	MYB	NcSABATH11	1887	1901	-	16.9531	9.86E-07	0.0296	CAGGTGGTAGGTGGT
AT1G79180	MYB	NcSABATH14	1334	1354	+	17.0156	9.93E-07	0.0226	ctaggctctcaacaaccccac
AT1G79180	MYB	NcSABATH11	1873	1893	+	17.0156	9.93E-07	0.0226	aaaaccaaccacctaccacct
