# Supplementary material for: Genome-wide identification and expression pattern analysis of the SABATH gene family in Neolamarckia cadamba
Source: For Res (Fayettev). 2023 May 29;3:13. doi: 10.48130/FR-2023-0013 (PMC11524262; doi:10.48130/FR-2023-0013)
Supplement: Supplementary file 1 — Supplementary data to this article can be found online. [file FR-2023-0013-S1.zip › 10.48130_FR-2023-0013-Suppl-TableS6.pdf]

Table S6. K_a/K_s analysis of duplication pairs of *NcSABATH* genes family in *N. cadamba*

Duplicated gene pairs	Non synonymous (K_a)	Synonymous (K_s)	K_a/K_s	Purifying selection	Subgroup	Duplicated type
NcSABATH4 & *NcSABATH5*	0.458166	1.282878	0.357139	yes	III	tandem
NcSABATH9 & *NcSABATH10*	0	0.008197	0	yes	I	tandem
NcSABATH4 & *NcSABATH19*	0.386885	2.077998	0.186182	yes	III	segmental
NcSABATH6 & *NcSABATH18*	0.25699	0.689902	0.372501	yes	III	segmental
NcSABATH3 & *NcSABATH12*	0.064768	0.347119	0.186589	yes	I	segmental
NcSABATH7 & *NcSABATH22*	0.048999	0.495425	0.098904	yes	III	segmental
NcSABATH9 & *NcSABATH16*	0.092278	0.488866	0.188759	yes	I	segmental
NcSABATH8 & *NcSABATH21*	0.037685	0.415785	0.090635	yes	II	segmental
NcSABATH13 & *NcSABATH17*	0.458895	4.766299	0.096279	yes	III	segmental
NcSABATH6 & *NcSABATH13*	0.360843	NaN	NaN		III	segmental
NcSABATH3 & *NcSABATH15*	0.512941	NaN	NaN		I	segmental
NcSABATH12 & *NcSABATH15*	0.501423	NaN	NaN		I	segmental
